# CDK8 remodels the tumor microenvironment and promotes resistance to KRAS^G12D^ inhibitors and daraxonrasib in PDAC

**DOI:** 10.1038/s44318-026-00854-5

**Published:** 2026-07-11

**Authors:** Kathleen M McAndrews, Krishnan K Mahadevan, Bingrui Li, Anaïs M Comptdaer, Amari M Sockwell, Sami J Morse, Patience J Kelly, Sarah I Patel, Michelle L Kirtley, Barbara A Moreno Diaz, Hengyu Lyu, David H Peng, Sima Khazaei, Aislyn Schalck, Xunian Zhou, Hikaru Sugimoto, Aaron A Bickert, Lakshmi Kavitha Sthanam, Meagan R Taylor, Shreyasee V Kumbhar, Kent A Arian, Yasaman Barekatain, Francesca Paradiso, Paola A Guerrero, Vincent Bernard, Navid Sobhani, Alondra N Camacho-Acevedo, Kiara E Bornes, Phuong Thao Tran, Anirban Maitra, Timothy P Heffernan, Raghu Kalluri

**Affiliations:** 1 Department of Cancer Biology, Metastasis Research Center, University of Texas MD Anderson Cancer Center, Houston, TX USA; 2 TRACTION Platform, Therapeutics Discovery Division, The University of Texas MD Anderson Cancer Center, Houston, TX USA; 3 Department of Translational Molecular Pathology, Sheikh Ahmed Center for Pancreatic Cancer Research, The University of Texas MD Anderson Cancer Center, Houston, TX USA; 4 Department of Radiation Oncology, The University of Texas MD Anderson Cancer Center, Houston, TX USA; 5 Department of Pathology, The University of Texas MD Anderson Cancer Center, Houston, TX USA; 6 Department of Bioengineering, Rice University, Houston, TX USA; 7 Department of Molecular and Cellular Biology, Baylor College of Medicine, Houston, TX USA; 8 Department of Pathology, University of Texas Medical Branch, Galveston, TX USA

**Keywords:** Cancer, Immunology

## Abstract

Mutations in *KRAS* are a dominant driver of pancreatic ductal adenocarcinoma (PDAC), with about 50% of patients presenting with *KRAS*^*G12D*^ mutations. Small molecule inhibitors targeting KRAS^G12D^ suppress PDAC; however, the contribution of the tumor microenvironment (TME) to the sustained efficacy of KRAS^G12D^ inhibition and mechanisms of resistance to KRAS^G12D^ suppression remain to be elucidated. Here, integrated spatial transcriptomics, single-cell RNA sequencing, and CODEX-based spatial proteomics analyses of PDAC mouse models uncover that while KRAS^G12D^ inhibition by MRTX1133 initially increases CD11c^+^ cells and T cell infiltration proximal to cancer cells, long-term treatment results in reversal of the immune responses leading to resistance promoted by multiprotein mediator complex associated kinase CDK8. CDK8 imparts this resistance via induction of CXCL2 chemokine secretion, inhibition of FAS expression, and remodeling of the TME to promote immune evasion. Targeting CDK8 by itself or in combination with αCTLA-4 immunotherapy overcomes resistance to KRAS^G12D^ inhibition. We also provide evidence of CDK8 upregulation in PDX tumors resistant to inhibitors selective for RAS(ON) and RAS^G12D^(ON): daraxonrasib and zoldonrasib, respectively, highlighting a common KRAS vulnerability node for TME resistance.

## Synopsis

Targeting of oncogenic *KRAS* mutations suppresses cancer growth, however relapse arises by unclear mechanisms. This study identifies mediator complex kinase CDK8 as a driver of resistance towards KRAS^G12D^ inhibition in pancreatic ductal adenocarcinoma (PDAC), promoting stromal remodeling and immunosuppression.

- Priming with oncogenic *KRAS* inhibitors and subsequent combination treatment with checkpoint immunotherapy significantly delays or prevents resistance.
- Long-term inhibition of oncogenic KRAS alone induces eventual therapy resistance in PDAC mouse models.
- CDK8 is upregulated in PDX tumors resistant to inhibitors selective for RAS(ON) and RASG12D(ON) protein states, Daraxonrasib and Zoldonrasib, respectively.
- CDK8 mediates KRAS inhibition resistance via reprogramming the tumor microenvironment and deficiency in immunological memory response.
- CDK8 confers resistance by re-initiating suppression of FAS expression and inducing secretion of the chemokine CXCL2.
- Targeting CDK8 overcomes resistance to KRAS^G12D^ inhibition.
- While tumors resistant to oncogenic KRAS inhibition also lose the ability to respond to anti-CTLA-4 immunotherapy, CDK8 inhibition in resistant tumors re-primes PDAC to anti-CTLA-4 immunotherapy efficacy.

## Abstract (web summary)

Mediator complex kinase CDK8 is a novel common vulnerability for stromal resistance towards oncogenic RAS inhibition in pancreatic cancer.

## Graphical abstract

**Figure:**
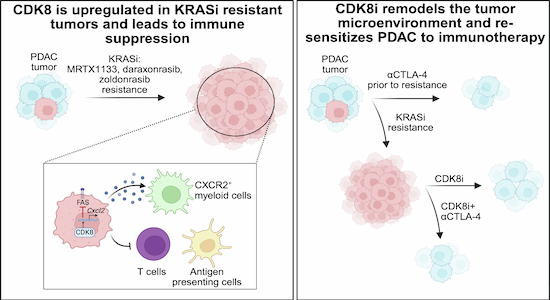

**Figure:**
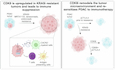

## Introduction

Pancreatic ductal adenocarcinoma is characterized by tissue-specific genetic events, including frequent mutations in *KRAS*. Specifically, *KRAS*^*G12D*^ mutations occur in approximately 40% of patients (Raphael et al, 2017) and genetically engineered mouse models (GEMMs) have revealed that oncogenic KRAS (KRAS*) is critical for PDAC initiation and maintenance (Aguirre et al, 2003; Collins et al, 2012; Hingorani et al, 2003; Mahadevan et al, 2023a; Ying et al, 2012), suggesting that targeting KRAS* may provide therapeutic benefits. KRAS* promotes the expression of pERK and reprograms cancer cell metabolism to promote glucose uptake and glycolysis, which is thought to be critical for supporting epithelial proliferation (Collins et al, 2012; Ying et al, 2012). In addition to its role in promoting cancer cell growth and other cancer cell intrinsic signaling pathways, KRAS* has also been implicated in remodeling of the tumor microenvironment (TME). KRAS* induces desmoplasia in part through the activation of the Hedgehog signaling pathway in epithelial cells (Collins et al, 2012) and by promoting the expression of inflammatory genes (Velez-Delgado et al, 2022). KRAS* promotes the epigenetic silencing of FAS, preventing CD8^+^ T cell-mediated induction of cancer cell apoptosis (Mahadevan et al, 2023a; Zhang et al, 2017) suggesting that KRAS* generates an immunosuppressive microenvironment. The recent development of KRAS* small molecule inhibitors has enabled therapeutic targeting of KRAS*, and such inhibitors impact cancer cell intrinsic signaling as well as reprogram the TME (Kemp et al, 2023; Mahadevan et al, 2023b). MRTX1133 was developed as a small molecule non-covalent inhibitor of KRAS^G12D^ that displays selective binding to the GDP-bound inactive form of KRAS^G12D^ (Hallin et al, 2022). MRTX1133 has been shown to inhibit proliferation and downstream KRAS signaling, ascertained by measuring the surrogate readouts of KRAS activity pERK and pS6, in several KRAS^G12D^ mutant PDAC cell lines and animal models (Hallin et al, 2022; Kemp et al, 2023; Liu et al, 2025; Mahadevan et al, 2023b). In addition to the impact of MRTX1133 on cancer cell autonomous signaling and proliferation, previous studies have implicated a role for the immune TME in facilitating responses to MRTX1133 and provided a rationale for combining MRTX1133 with immune checkpoint blockade (ICB) (Kemp et al, 2023; Liu et al, 2025; Mahadevan et al, 2023b).

While KRAS* small molecule inhibitors have demonstrated effective tumor control in xenograft, syngeneic orthotopic models, and spontaneous mouse models, prolonged KRAS* inhibition ultimately leads to tumor relapse (Hallin et al, 2022; Kemp et al, 2023; Mahadevan et al, 2023b), similar to what has been reported with genetic KRAS* suppression and inhibition of the KRAS* and receptor tyrosine kinase (RTK) signaling pathways with small molecule inhibitors (Holohan et al, 2013; Kapoor et al, 2014; Viale et al, 2014). Earlier studies suggested that PDAC cancer cells that are resistant to genetic KRAS* suppression are metabolically dependent on oxidative phosphorylation and in some contexts display amplification of *Yap*, which acts to promote KRAS*-independent growth (Kapoor et al, 2014; Viale et al, 2014). Moreover, *Hdac5* was identified as promoting KRAS*-independent growth through increased CCL2 in cancer cells, which acts to promote the infiltration of CCR2^+^ macrophages (Hou et al, 2020). Several potential mechanisms of KRAS* small molecule inhibitor resistance have been proposed in the context of resistance to small molecule inhibition of KRAS^G12C^, including mutations in the drug binding pocket of kinases, increased signaling through alternative pathways to promote growth, and dysregulation of the cell cycle (Amodio et al, 2020; Awad et al, 2021; Hallin et al, 2020); however, there is emerging evidence that subtypes of *KRAS* mutations may have distinct functional outcomes. KRAS* allele specific progression of PanIN lesions occurs in GEMMs, suggesting that common and divergent mechanisms of KRAS* inhibition may exist (Zafra et al, 2020). Moreover, KRAS^G12C^ and KRAS^G12D^ have different intrinsic and GAP-stimulated GTP hydrolysis rates (Hunter et al, 2015), which may impact small molecule inhibitor binding to the inactive and active states of KRAS* and overall KRAS* inhibition efficacy.

Mechanism(s) of resistance to small molecule inhibition of KRAS^G12D^ remain to be unraveled. Here, we identify CDK8 as promoting KRAS* inhibition resistance in PDAC through its suppression of FAS expression and induction of CXCL2, which act to promote immunosuppression. Combined inhibition of KRAS* with CDK8 or the CXCL2 receptor CXCR2 inhibits tumor growth and prolongs survival, indicating a role for the immune microenvironment in shaping resistance to a small molecule inhibitor of KRAS*.

## Results

### Long term KRAS* inhibition leads to therapy resistance

**Figure 1:**
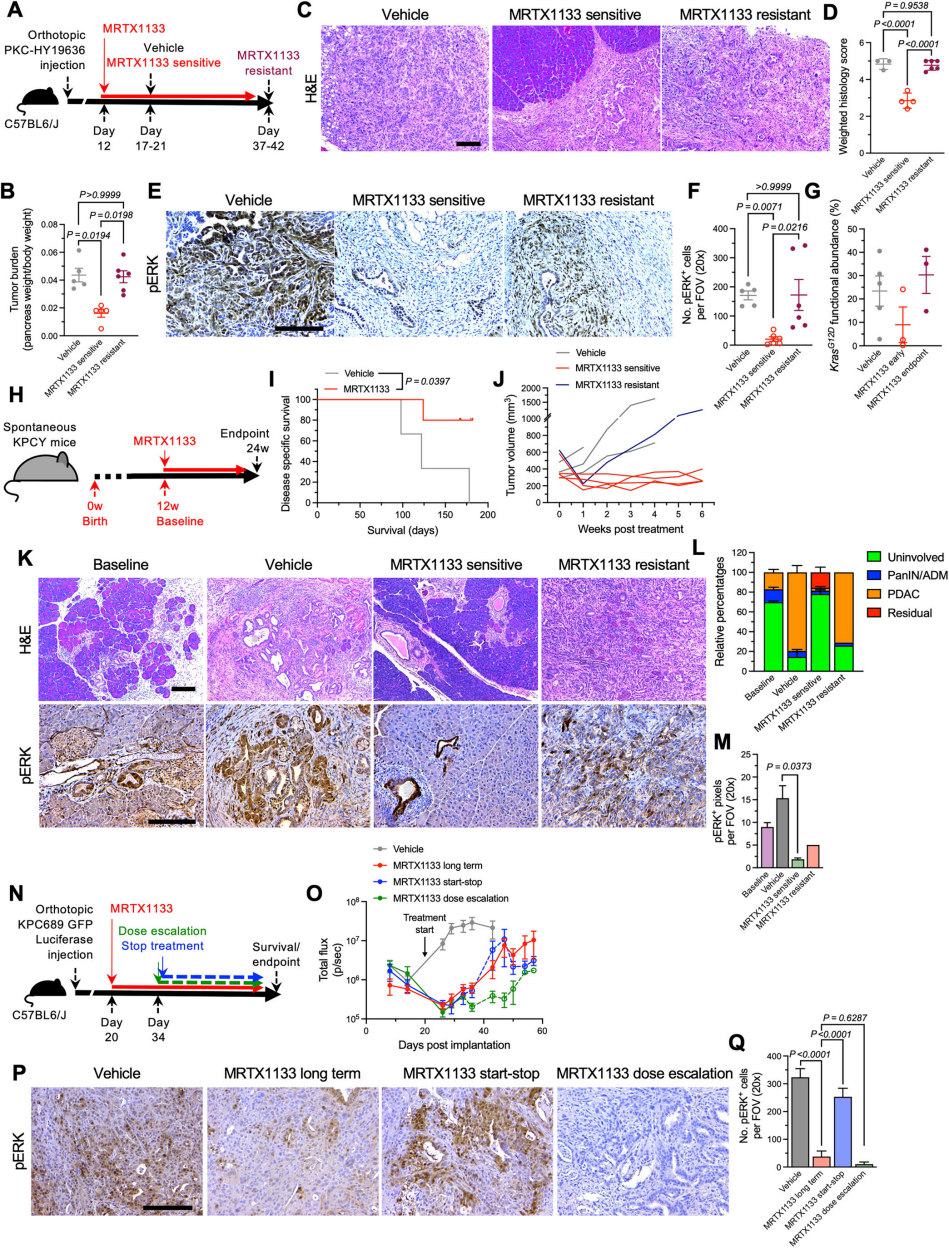
Murine PDAC models demonstrate resistance to KRAS^G12D^ inhibition. (**A**) Schematic of orthotopic PKC-HY19636 tumors treated with MRTX1133. (**B**) Tumor burden of PKC-HY19636 tumors. Vehicle, *n* = 5; MRTX1133 sensitive, *n* = 5; MRTX1133 resistant, *n* = 6. (**C**, **D**) Representative H&E images (**C**) and quantification (**D**) of orthotopic PKC-HY19636 tumors. Vehicle, *n* = 3; MRTX1133 sensitive, *n* = 4; MRTX1133 resistant, *n* = 6. (**E**, **F**) Representative pERK staining (**E**) and quantification (**F**) of orthotopic PKC-HY19636 tumors. Vehicle, *n* = 5; MRTX1133 sensitive, *n* = 6; MRTX1133 resistant, *n* = 6. (**G**) ddPCR for *Kras*^*G12D*^ functional abundance in PKC-HY19636 orthotopic tumors. Vehicle, *n* = 5; MRTX1133 sensitive, *n* = 3; MRTX1133 resistant, *n* = 3. (**H**) Schematic of spontaneous KPCY GEMM tumors treated with MRTX1133. (**I**) Survival curve of KPCY GEMM mice (days post-birth). Vehicle, *n* = 3; MRTX1133, *n* = 5. (**J**) Tumor volume of KPCY GEMMs measured by MRI. Vehicle, *n* = 3; MRTX1133 sensitive, *n* = 4; MRTX1133 resistant, *n* = 1. (**K**–**M**) Representative H&E and pERK images (**K**), histological assessment (**L**), and pERK quantification (**M**). Baseline, *n* = 2; Vehicle, *n* = 3; MRTX1133 sensitive, *n* = 4; MRTX1133 resistant, *n* = 1. Scale bars, 100 μm. (**N**) Schematic of orthotopic KPC689 tumors treated with MRTX1133. (**O**) Bioluminescence of orthotopic KPC689 tumors in C57BL6/J mice over time. Treatment was initiated at day 20 (Vehicle: *n* = 8, MRTX1133 long term: *n* = 9, MRTX1133 start-stop: *n* = 8, MRTX1133 dose escalation: *n* = 9). Dashed lines denote where treatment was stopped for MRTX1133 start-stop and dosage increased for MRTX1133 dose escalation. (**P**, **Q**) Representative pERK images (**P**) quantification (**Q**) of orthotopic KPC689 tumors. Vehicle: *n* = 4, MRTX1133 long term: *n* = 5, MRTX1133 start-stop: *n* = 3, MRTX1133 dose escalation: *n* = 6. Kruskal–Wallis with Dunn’s multiple comparisons test performed in (**B**, **F**). One-way ANOVA with Tukey’s multiple comparisons test performed in (**D**). Log-rank test performed in (**I**). Unpaired two-tailed *t* test with Welch’s correction performed in (**M**). One-way ANOVA with Dunnett’s multiple comparisons test performed in (**Q**). Scale bars, 100 μm. Data are presented as mean ± s.e.m. Exact *P* values reported.

**Figure EV1:**
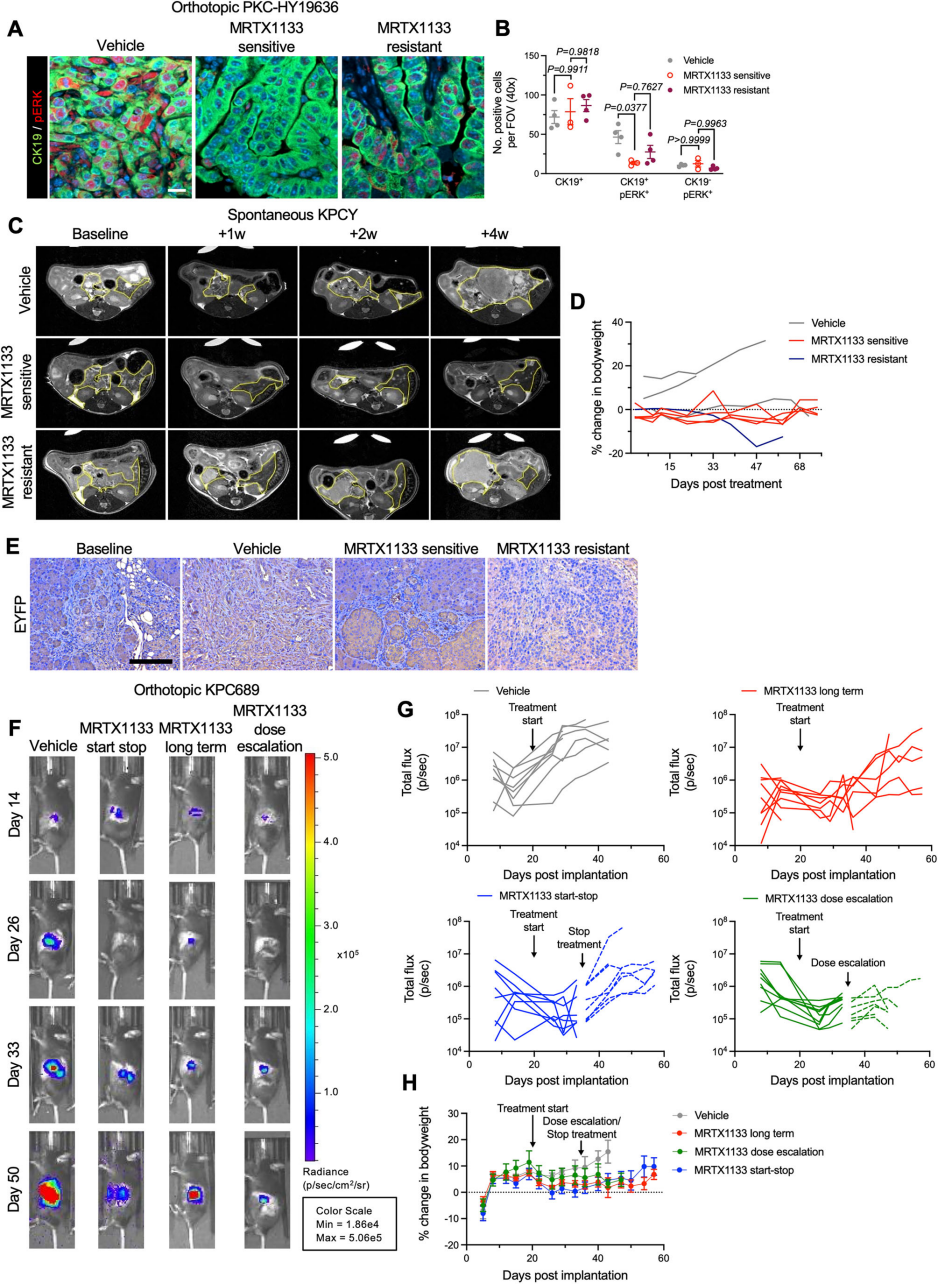
Anti-PDAC responses of spontaneous and orthotopic KPC models to MRTX1133. (**A**, **B**) Representative immunostaining images (**A**) and quantification of CK19^+^, CK19^+^pERK^+^, and CK19^-^pERK^+^ cells in orthotopic PKC-HY19636 tumors (**B**). Vehicle, *n* = 4; MRTX1133 sensitive, *n* = 3; MRTX1133 resistant, *n *= 4. Scale bar, 20 μm. (**C**) MRI of spontaneous KPCY models. Yellow lines demarcate tumor regions. (**D**) Bodyweight of KPCY mice during treatment. Vehicle, *n* = 3; MRTX1133 sensitive, *n* = 4; MRTX1133 resistant, *n* = 1. (**E**) Representative images of EYFP lineage tracer stains of spontaneous KPCY tumors. Scale bar, 100 μm. (**F**, **G**) Representative bioluminescence images (**F**) and individual bioluminescence curves (**G**) of KPC689 tumors. Vehicle: *n* = 8, MRTX1133 long term: *n* = 9, MRTX1133 start-stop: *n* = 8, MRTX1133 dose escalation: *n* = 9. (**H**) Bodyweight of mice bearing orthotopic KPC689 tumors post tumor-implantation. Vehicle: *n* = 8, MRTX1133 long term: *n* = 9, MRTX1133 start-stop: *n* = 8, MRTX1133 dose escalation: *n* = 9. Data are presented as mean ± s.e.m. except in (**D**, **G**) where individual mice are plotted. One-way ANOVA with Sidak’s multiple comparisons test performed in (**B**). Exact *P* values reported.

To evaluate the impact of long-term KRAS* inhibition on PDAC progression and resistance, multiple mouse models of PDAC were treated with MRTX1133. *Trp53*^*F/+*^*; LSL-Kras*^*G12D/+*^*; p48-Cre* (PKC-HY19636) cells were orthotopically implanted in the pancreata of syngeneic C57BL/6 mice and mice treated with the non-covalent small molecule KRAS* inhibitor MRTX1133 (Fig. 1A). Mice treated with MRTX1133 (30 mg/kg BID i.p.) demonstrated significantly reduced tumor burden at one-week post-treatment initiation (MRTX1133 sensitive); however, mice with continued MRTX1133 treatment (MRTX1133 resistant, approximately 2–3 weeks of treatment) ultimately relapsed and displayed similar tumor burden to control (vehicle) treated mice (Fig. 1B). Histological assessment further confirmed tumor relapse in MRTX1133 resistant mice (Fig. 1C,D). Reduced pERK^+^ cells were observed in MRTX1133 treated tumors at early timepoints, indicative of suppression of KRAS signaling, whereas pERK^+^ cells in MRTX1133 resistant tumors were heterogeneous, with some tumors having high numbers and others having lower numbers of pERK^+^ cells (Fig. 1E,F). Costaining for CK19 and pERK revealed specific downregulation of pERK in CK19^+^ cancer cells in MRTX1133 sensitive tumors and heterogeneous responses in MRTX1133 resistant tumors (Fig. EV1A,B), similar to IHC results (Fig. 1E,F). Moreover, CK19^-^pERK^+^ cells were not changed in MRTX1133 treated mice (Fig. EV1A,B), supporting specific targeting of mutant KRAS^G12D^ as previously reported (Hallin et al, 2022; Kemp et al, 2023). The *Kras*^*G12D*^ mutation was detected in vehicle, MRTX1133 sensitive, and MRTX1133 resistant tumors (Fig. 1G), indicating that resistance is associated with outgrowth of *Kras*^*G12D*^ mutant cells.

In the context of spontaneously occurring *LSL-Trp53*^*R172H/+*^; *LSL-Kras*^*G12D/+*^*; Pdx1-Cre; LSL-EYFP* (KPCY) tumors, increased survival and tumor growth control was observed in response to MRTX1133, albeit in a limited number of mice (30 mg/kg BID i.p., Fig. 1H,I). Like orthotopic models, tumor regrowth following MRTX1133 treatment was observed at 2–5 weeks post-treatment initiation (MRTX1133 resistant, Figs. 1J and EV1C) without impacting bodyweight during treatment (Fig. EV1D). Tumors that responded to MRTX1133 treatment (MRTX1133 sensitive) had increased uninvolved tissue relative to vehicle treated tumors as evaluated by H&E staining (Fig. 1K,L). EYFP, a lineage marker of acinar cells and cancer cells, immunostaining was performed and evaluated in conjunction with H&E stains to identify the presence of cancer cells derived from the *Pdx1*^+^ (acinar) cell lineage (Fig. EV1E). pERK was suppressed in MRTX1133 sensitive tumors with only marginal upregulation in the resistant tumor (Fig. 1K,M), suggesting suppression of KRAS^G12D^ in spontaneous PDAC models.

A second orthotopic model was employed to confirm emergence of resistance to KRAS* inhibition, wherein KPC689 GFP (green fluorescent protein)-luciferase orthotopic tumor bearing mice were treated with MRTX1133 and tumor kinetics were monitored with IVIS imaging (Fig. 1N). MRTX1133 treatment was associated with initial tumor regression followed by relapse within 14 days in the KPC689 GFP-luciferase model (Figs. 1O and EV1F,G). After 14 days of treatment, one group of mice was maintained on MRTX1133 treatment (MRTX1133 long term), one group of mice were enrolled in a higher dose of MRTX1133 (MRTX1133 dose escalation, 60 mg/kg BID i.p.) and treatment stopped on another group of mice (MRTX1133 start-stop, Fig. 1N). Bodyweight was similar across treatment groups following treatment enrollment (Fig. EV1H). Dose escalation initially controlled tumor growth but ultimately was associated with resistance to KRAS* inhibition and PDAC regrowth (Figs. 1N,O and  EV1F,G; Appendix Fig. S1A,B). Dose escalation was also associated with non-PDAC related death, suggesting that toxicity may exist at higher doses of MRTX1133 despite no overt changes in bodyweight (Fig. EV1H). Treatment cessation had no impact on tumor growth (Figs. 1O and EV1F,G; Appendix Fig. S1A,B). MRTX1133 long term and MRTX1133 dose escalation tumors both demonstrated reduced pERK^+^ cells, whereas pERK^+^ cells were increased in vehicle and MRTX1133 start and stop treatment tumors (Fig. 1P,Q). Emergence of resistance was further validated in another PDAC mouse model using the KPC-T cell line. This cell line was established after serial transplantation of a tumor from KPC mice (KPC-T) (Foley et al, 2015). KPC-T cells were orthotopically implanted into C57BL/6 mice and treated with MRTX1133 (Appendix Fig. S1C). MRTX1133 significantly increased the overall survival of mice and led to control of tumor growth at 1 week following treatment; however, prolonged treatment was associated with tumor reemergence (Appendix Fig. S1D–F). MRTX1133 sensitive tumors, 1 week following MRTX1133 treatment, showed reduced tumor burden by histological analysis and pERK suppression, whereas increased tumor burden and sustained suppression of pERK was observed in MRTX1133 resistant tumors (Appendix Fig. S1E–G), supporting the emergence of resistance to MRTX1133 in multiple models of PDAC.

### KRAS* inhibitor resistant cancer cells upregulate CDK8

**Figure 2:**
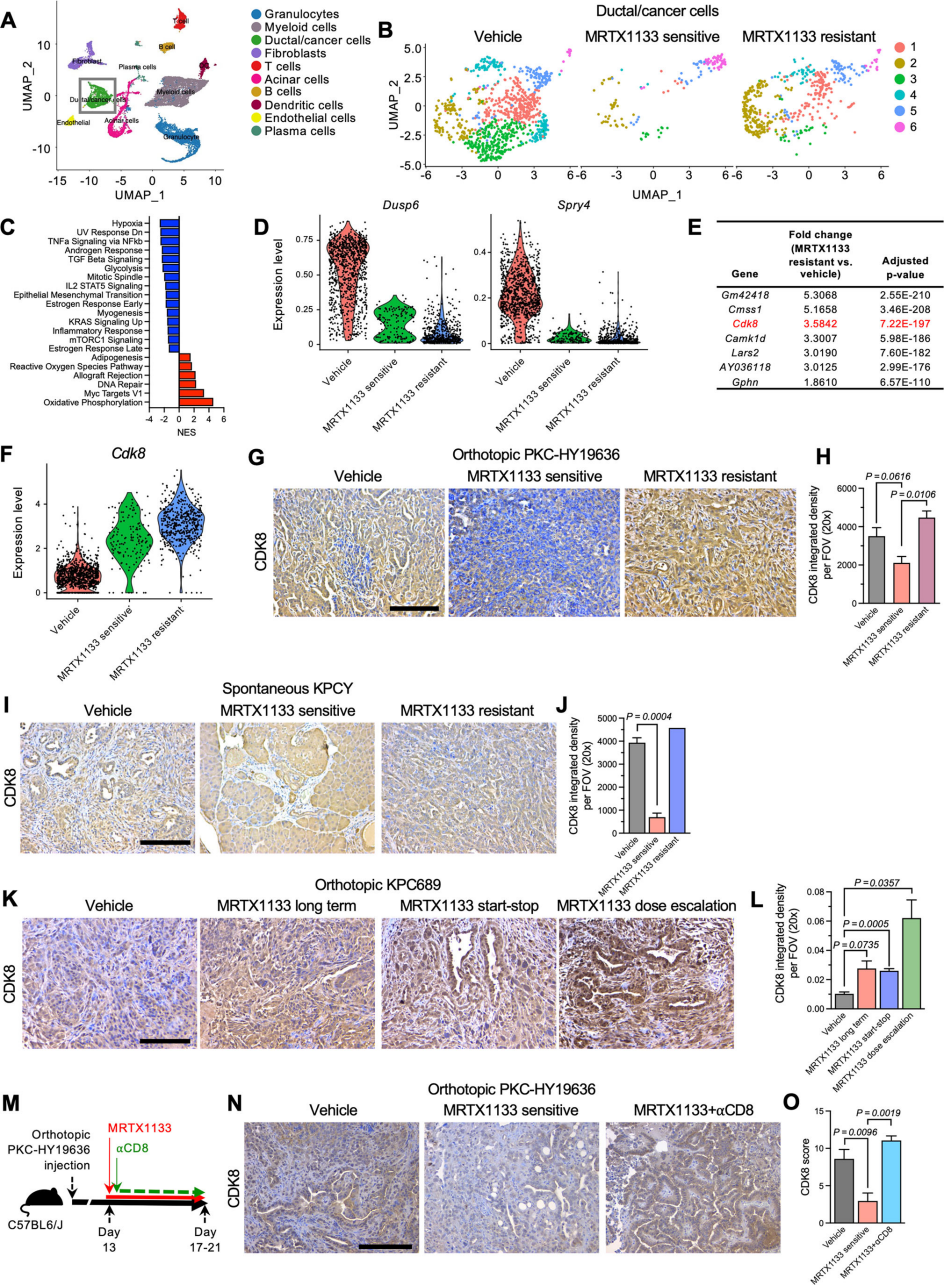
CDK8 is upregulated in MRTX1133 resistant cancer cells. (**A**) UMAP of orthotopic PKC-HY19636 tumors determined by scRNA-seq. Pancreata/tumors from 2-3 mice pooled per group. (**B**) UMAP of ductal/cancer cells of orthotopic PKC-HY19636 tumors determined by scRNA-seq. (**C**) Enriched pathways in ductal/cancer cells of MRTX1133 resistant tumors compared to vehicle orthotopic PKC-HY19636 tumors. (**D**) Violin plots of *Dusp6* and *Spry4* in ductal/cancer cells of orthotopic PKC-HY19636 tumors. (**E**) Significantly upregulated genes in ductal/cancer cells of MRTX1133 resistant tumors compared to vehicle orthotopic PKC-HY19636 tumors. (**F**) Violin plot of *Cdk8* in ductal/cancer cells of orthotopic PKC-HY19636 tumors. (**G**, **H**) Representative CDK8 staining (**G**) and quantification (**H**) of orthotopic PKC-HY19636 tumors. Vehicle, *n* = 6; MRTX1133 sensitive, *n* = 4; MRTX1133 resistant, *n* = 3. (**I**,** J**) Representative CDK8 staining (**I**) and quantification (**J**) of spontaneous KPCY GEMM tumors. Vehicle, *n* = 3; MRTX1133 sensitive, *n* = 3; MRTX1133 resistant, *n* = 1. (**K**, **L**) Representative CDK8 staining (**K**) and quantification (**L**) of orthotopic KPC689 tumors. Vehicle: *n* = 4, MRTX1133 long term: *n* = 5, MRTX1133 start-stop: *n* = 4, MRTX1133 dose escalation: *n* = 5. (**M**) Schematic representation of orthotopic PKC-HY19636 tumors treated with MRTX1133 for 5–9 days. (**N**, **O**) Representative CDK8 staining (**N**) and quantification (**O**) of orthotopic PKC-HY19636 tumors. Vehicle, *n* = 7 mice; MRTX1133 sensitive, *n* = 4 mice; MRTX1133 + αCD8, *n* = 4 mice. One-way ANOVA with Dunnett’s multiple comparisons test performed in (**H**, **O**). Unpaired two-tailed *t* test with Welch’s correction performed in (**J**). Brown–Forsythe and Welch ANOVA with Dunnett T3’s multiple comparisons test performed in (**L**). Scale bars, 100 μm. Data are presented as mean ± s.e.m. except for in (**D**, **F**) where it is presented as violin plots. Exact *P* values reported.

To evaluate the transcriptional changes resulting from MRTX1133 treatment, single cell RNA sequencing (scRNA-seq) was employed, and ductal/cancer cells specifically analyzed with 6 transcriptional clusters of cells identified (Fig. 2A,B; Appendix Fig. S2A–C). InferCNV was performed to identify cancer cells within the ductal/cancer cell cluster, with tumor-derived B cells and T and NK cells used as a reference (Appendix Fig. S3A). Cluster 6 specifically lacked overt deletions and amplifications, like the reference cells, suggesting that this cluster likely consists of untransformed ductal cells (Appendix Fig. S3A). In MRTX1133 sensitive, cluster 6 is the primary cluster represented, supporting effective elimination of cancer cells with treatment (Fig. 2B). Gene set enrichment analysis (GSEA) of MRTX1133 resistant tumors compared to vehicle tumors revealed downregulation of a number of pathways previously associated with KRAS*, including downregulation of hypoxia, glycolysis, KRAS signaling, and mTORC1 signaling, and upregulation of MYC targets and oxidative phosphorylation (Fig. 2C; Appendix Fig. S3B) (Dey et al, 2020; Viale et al, 2014; Ying et al, 2012). Further analysis of KRAS pathway related genes, *Dusp6* and *Spry4* revealed downregulation with MRTX1133 treatment that was maintained in resistant tumors (Fig. 2D), indicating effective suppression of KRAS* signaling. Previous studies have implicated a rebound of MAPK pathway activity, driven by enhanced wildtype KRAS signaling or receptor tyrosine kinase (RTK) signaling, in resistance to KRAS^G12C^ inhibitors (Amodio et al, 2020; Ryan et al, 2022); however, our data suggest that rebound of MAPK signaling does not occur in all contexts (Figs. 1F and 2C,D).

**Figure EV2:**
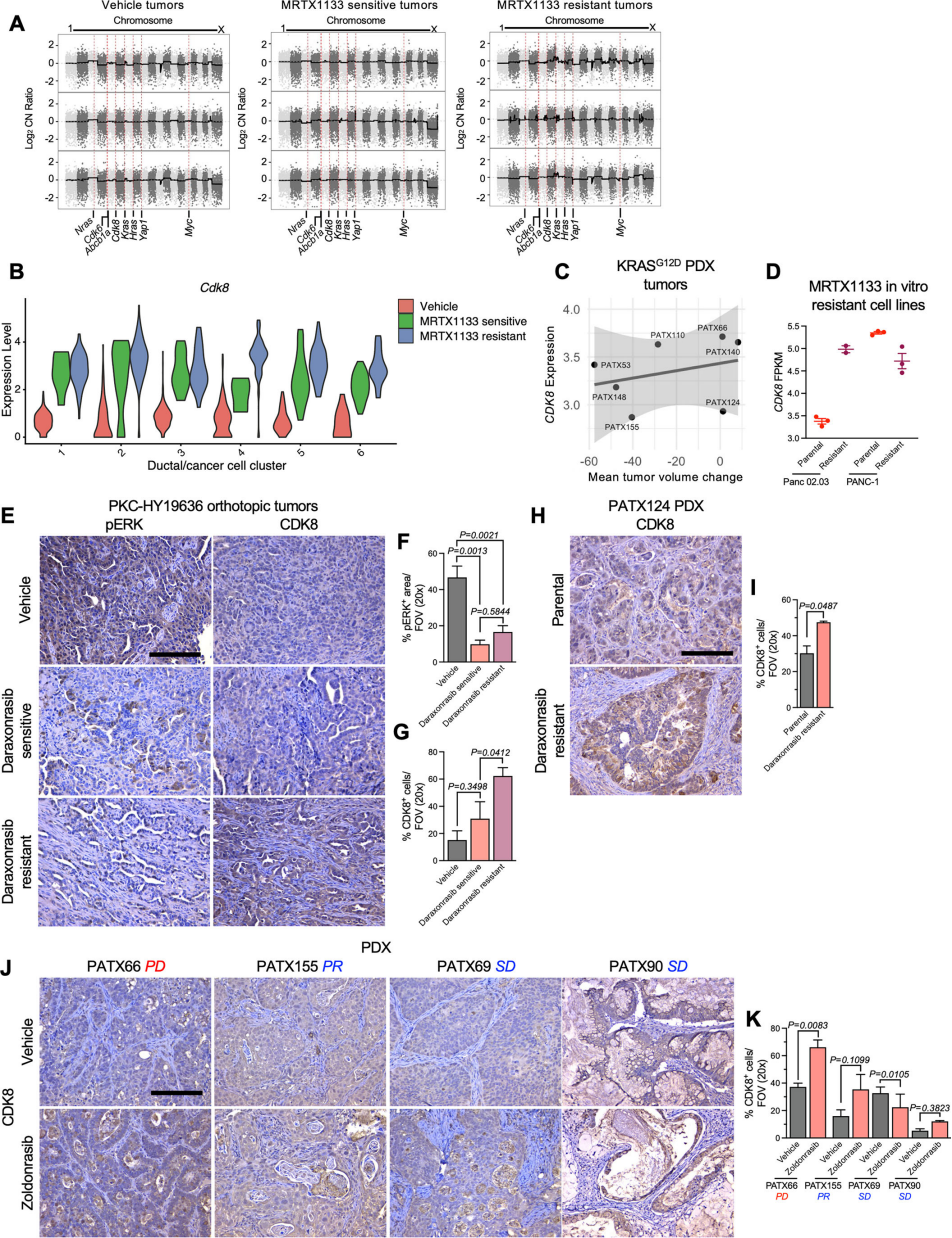
CDK8 is upregulated in PDAC resistant to MRTX1133, Daraxonrasib, and Zoldonrasib. (**A**) Genome-wide copy number plots of vehicle, MRTX1133 sensitive, and MRTX1133 resistant orthotopic PKC-HY19636 tumors with tail gDNA used as a reference. *n* = 3 tumors per group. (**B**) Violin plot of *Cdk8* expression in ductal/cancer cell clusters of vehicle, MRTX1133 sensitive, and MRTX1133 resistant orthotopic PKC-HY19636 tumors analyzed by scRNA-seq. (**C**) *CDK8* expression and mean tumor volume change of the indicated PDXs treated with MRTX1133. (**D**) *CDK8* expression in parental and MRTX1133 resistant cell lines. Data derived from GSE269985. (**E**–**G**) Representative images (**E**) and quantification of pERK (**F**) and CDK8 (**G**) immunostaining of orthotopic PKC-HY19636 tumors treated with daraxonrasib. Daraxonrasib treatment was started on day 12 post-orthotopic tumor implantation. Vehicle, *n* = 4; Daraxonrasib sensitive (10 days post-treatment start), *n* = 3; Daraxonrasib resistant (mice reached end point, day 22–32 post-orthotopic tumor implantation), *n* = 5. (**H**, **I**) Representative CDK8 immunostaining (**H**) and quantification (**I**) of orthotopic PATX124 parental and Daraxonrasib resistant PDXs. Parental, n = 4; Daraxonrasib resistant, *n* = 2. (**J**, **K**) Representative CDK8 immunostaining (**J**) and quantification (**K**) of PDXs treated with Zoldonrasib. PD progressive disease, PR partial response, SD stable disease. Responses reported are best responses. PATX66, *n* = 3 per group; PATX155 vehicle, *n* = 4; PATX155 Zoldonrasib, *n* = 2; PATX69 vehicle, *n* = 4; PATX69 Zoldonrasib, *n* = 2; PATX90, *n* = 3 per group. Scale bars, 100 μm. One-way ANOVA with Tukey’s multiple comparisons test performed in (**F**), one-way ANOVA with Dunnett’s multiple comparisons test performed in (**G**), unpaired two-tailed *t* test performed in (**I**, **K**). Exact *P* values reported.

Whole-exome sequencing (WES) was performed to further understand the genetic alterations associated with resistance to MRTX1133. MRTX1133 resistant tumors displayed heterogeneous copy number variation (CNV) patterns, with heterogeneous amplifications detected in *Cdk6*, *Abcb1a*, *Cdk8*, *Yap1*, and *Myc* (Fig. EV2A; Dataset EV1). In addition, two out of three tumors showed amplification of *Kras* (Fig. EV2A). In contrast to previous reports with KRAS^G12C^ inhibitors (Ryan et al, 2022; Zhao et al, 2021), development of *Nras* or *Hras* mutations or amplifications was not observed in resistant tumors (Fig. EV2A). Further analysis of cancer cells in PKC-HY19636 orthotopic tumors by scRNA-seq validated *Cdk8* as one of the top genes upregulated in MRTX1133 resistant cancer cells (Fig. 2E,F). Upregulation of *Cdk8* was present in all ductal/cancer cell clusters (Fig. EV2B), suggesting that it is not limited to a subset of cancer cells. Immunohistochemistry for CDK8 showed that CDK8 was suppressed in MRTX1133 sensitive tumors and upregulated in MRTX1133 resistant PKC-HY19636 and KPCY tumors (Fig. 2G–L). Interestingly, the MRTX1133 dose escalation group showed increased expression of CDK8 in orthotopic KPC689 tumors (Fig. 2K,L), suggesting that higher dosages of MRTX1133 may augment the selection for treatment resistant cells with high expression of CDK8.

*KRAS*^*G12D*^ mutant patient derived xenografts (PDXs) treated with MRTX1133 were analyzed for CDK8 expression at endpoint when resistance is established. At this timepoint, the association with CDK8 expression and diminished MRTX1133 efficacy was noted (Fig. EV2C). Analysis of previously reported RNA sequencing data from human PDAC cell lines resistant to MRTX1133 (Dilly et al, 2024) revealed that resistant Panc02.03 demonstrate an increase in CDK8 expression compared to parental cells, and similar expression to parental PANC-1, which are intrinsically resistant to MRTX1133 (Fig. EV2D) (Dilly et al, 2024). To validate whether CDK8 upregulation occurs in other types of RAS inhibition which are clinically relevant in PDAC, we focused specifically the RAS-MULTI(ON) inhibitor RMC6236 (Daraxonrasib) which was evaluated as a single agent in a phase 3 clinical trial and the RAS(ON) G12D-selective inhibitor RMC9805 (Zoldonrasib) which is being evaluated in a phase 1/1b clinical trial. Similar to what others have reported (Wasko et al, 2024), we found that long-term treatment with Daraxonrasib was associated with an emergence of resistance (Mahadevan et al, 2025). Daraxonrasib sensitive and resistant tumors have suppressed pERK compared to vehicle treated mice (Fig. EV2E,F), which may reflect its ability to suppress both amplified wildtype RAS signaling and mutant RAS signaling (Wasko et al, 2024). In addition, Daraxonrasib resistant tumors had increased CDK8^+^ cells compared to sensitive tumors (Fig. EV2E,G). We further evaluated a human KRAS^G12D^ PDX that developed resistance to Daraxonrasib (PATX124) and drug treatment stopped upon establishment of resistance. CDK8^+^ cells were increased in Daraxonrasib resistant tumors compared to parental tumors (Fig. EV2H,I). Human KRAS^G12D^ PDXs showed a diverse distribution of best responses to Zoldonrasib: partial response (PR), stable disease (SD), and progressive disease (PD). PATX66, which displayed resistance to Zoldonrasib and PD, had elevated CDK8^+^ cells with Zoldonrasib treatment compared to PDXs that had SD (Fig. EV2J,K). PATX155 had PR to Zoldonrasib and a trend towards increased CDK8, albeit not statistically significant (*P* = 0.1099, Fig. EV2J,K).

**Figure EV3:**
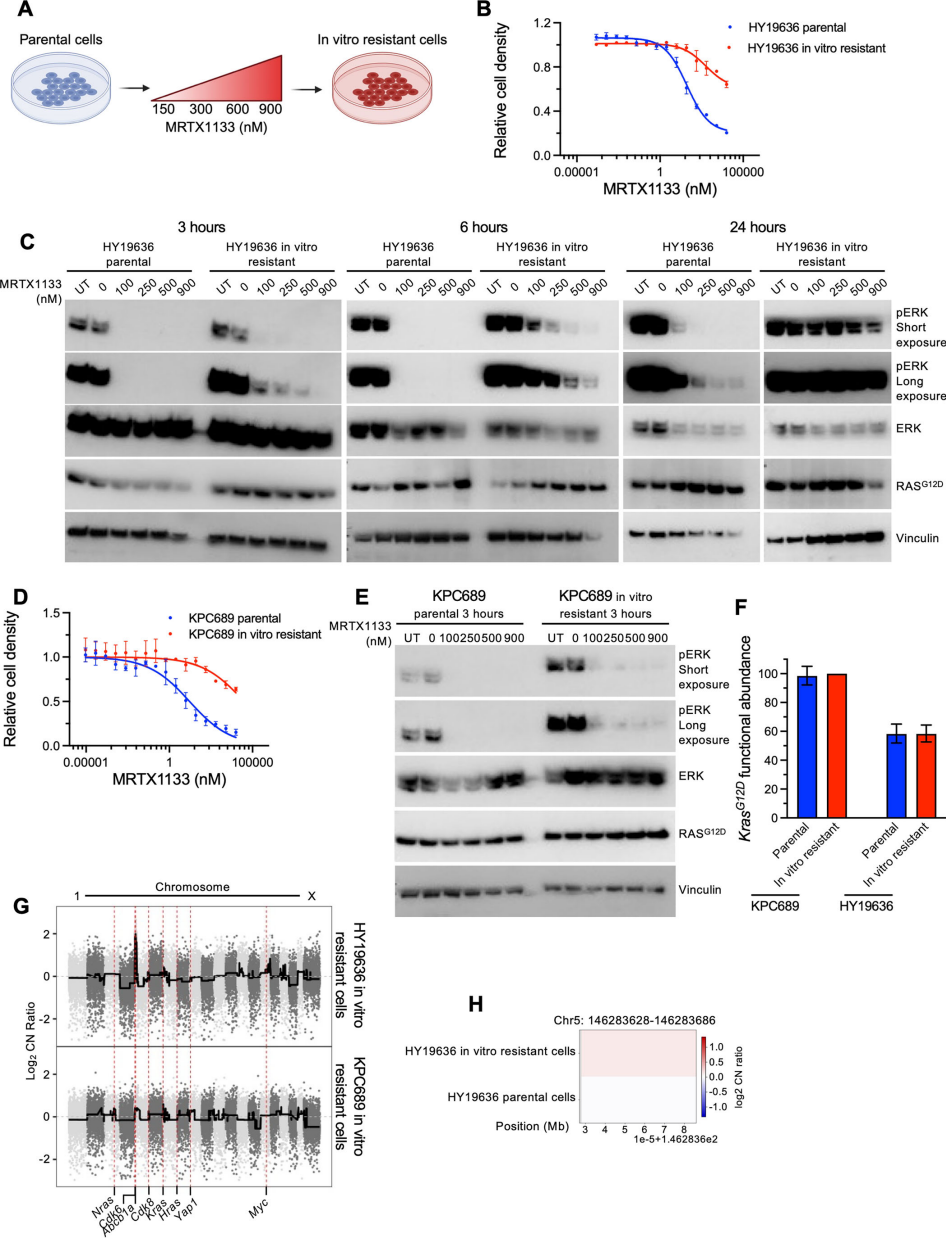
Characterization of in vitro MRTX1133 resistant cells. (**A**) Schematic of establishment of in vitro resistant cell lines, which were generated by exposing cells to increasing concentrations of MRTX1133 over the course of 2–3 months. (**B**) Relative cell density of HY19636 parental and HY19636 in vitro resistant cells treated with the indicated concentrations of MRTX1133 for 3 days. (**C**) Representative western blots of pERK, ERK, RAS^G12D^, and vinculin of HY19636 parental and HY19636 in vitro resistant cells treated with the indicated concentrations of MRTX1133 for 3 h, 6 h, or 24 h. (**D**) Relative cell density of KPC689 parental and KPC689 in vitro resistant cells treated with the indicated concentrations of MRTX1133 for 3 days. (**E**) Representative western blots of pERK, ERK, RAS^G12D^, and vinculin of KPC689 parental and KPC689 in vitro resistant cells treated with the indicated concentrations of MRTX1133 for 3 h. (**F**) ddPCR for *Kras*^*G12D*^ functional abundance in KPC689 and HY19636 parental and in vitro resistant cells. (**G**) Genome-wide copy number plots of HY19636 and KPC689 in vitro resistant with parental cell lines used as a reference. (**H**) WES data of the *Cdk8* gene of HY19636 parental and HY19636 in vitro resistant cells. Data are presented as mean ± s.d. Source data are available online for this figure.

To further evaluate resistance to MRTX1133 treatment, an in vivo resistant PKC-HY19636 cell line was generated. The in vivo resistant cell line demonstrated diminished suppression of pERK in response to MRTX1133 compared to parental cells (~2.5-fold increase in pERK IC50, Appendix Fig. S4A–D). Proliferation of PKC-HY19636 in vivo resistant cells in response to MRTX1133 was also increased compared to PKC-HY19636 parental (~1.6-fold increase in proliferation IC50, Appendix Fig. S4E,F), further supporting resistance to MRTX1133. In addition, in vitro resistant cell lines (HY19636 and KPC689 in vitro resistant) were established by exposing cells to increasing concentrations of MRTX1133 (Fig. EV3A). Similar to the in vivo resistant line, the in vitro resistant cells showed reduced sensitivity to MRTX1133 and similar levels of RAS^G12D^ expression compared to parental cells (Fig. EV3B–E). The *Kras*^*G12D*^ mutation was detected in parental and in vitro resistant KPC689 and PKC-HY19636 cell lines (Fig. EV3F). Whole-exome sequencing of in vitro resistant and parental cells revealed amplification of *Cdk8* in resistant cells (Fig. EV3G,H), which may contribute to the observed increased transcriptional expression of *Cdk8*. Collectively, our data demonstrates that resistance to KRAS* inhibition is a ubiquitous phenomenon and is associated with upregulation of CDK8 across multiple PDAC models.

In order to evaluate the impact of MRTX1133 in a preventative context, we treated *LSL-Kras*^*G12D/+*^*; Pdx1-Cre* (KC) mice, which develop spontaneous PanIN precursor lesions but typically do not progress to PDAC until 12-15 months, with MRTX1133 for up to 18 weeks (Hingorani et al, 2003). MRTX1133 treatment reduced PanIN lesions, as quantified by histological evaluation and CK19 staining, and pERK abundance (Appendix Fig. S5A–E). In contrast to orthotopic PKC-HY19636 tumors and spontaneous KPC GEMMs, KC mice do not present with resistance even upon long-term MRTX1133 treatment. CDK8 is not elevated in the pancreata of MRTX1133 treated mice (Appendix Figs. S5B and S5F) and a reduced spatial association of CD4^+^ and CD8^+^ memory/stem-like T cells with *Cdk8* high ductal/cancer cells was observed (Appendix Fig. S5G,H), indicating a potential role for the TME in preventing the emergence of precursor lesions in KC mice.

**Figure EV4:**
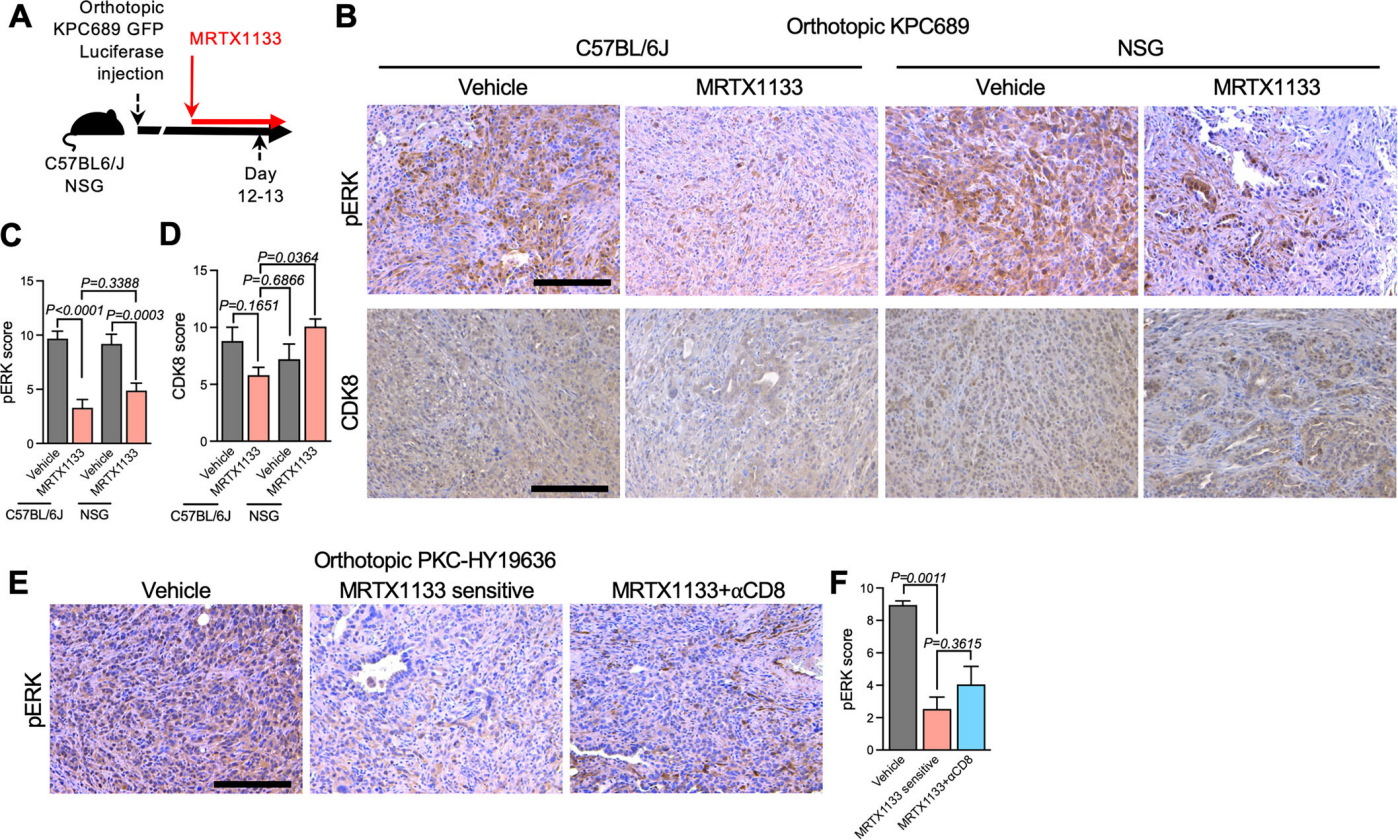
T cells control the emergence of CDK8^+^ resistant cancer cells. (**A**) Schematic of orthotopic KPC689 GFP-luciferase tumors in C57BL6/J and NSG mice treated with MRTX1133. (**B**) Representative pERK and CDK8 staining at 12–13 days post-treatment. (**C**, **D**) Quantification of pERK (**C**) and CDK8 (**D**) staining. pERK: C57BL6/J MRTX1133 and NSG vehicle, *n* = 4 mice; *n* = 5 mice per group for all other groups. CDK8: C57BL6/J MRTX1133, *n* = 4 mice; *n* = 5 mice per group for all other groups. (**E**, **F**) Representative pERK staining (**E**) and quantification (**F**) of orthotopic PKC-HY19636 tumors at 12–13 days post-treatment. Vehicle, *n* = 4; MRTX1133 sensitive, *n* = 3; MRTX1133 + αCD8, *n* = 4. Scale bars, 100 μm. One-way ANOVA with Dunnett’s multiple comparisons test performed in (**C**, **D**, **F**). Exact *P* values reported.

We next determined the contribution of immune cells to the emergence of CDK8^+^ resistant cancer cells. KPC689 cells were implanted into immunocompetent C57BL/6 J mice and immunodeficient NSG mice and treated with MRTX1133 for 12–13 days (Fig. EV4A). KPC689 tumors treated with MRTX1133 demonstrated reduced pERK in immunocompetent and immunodeficient mice (Fig. EV4B,C), indicating effective inhibition of KRAS* signaling in both contexts. In contrast, KPC689 tumors in NSG mice had increased CDK8 expression compared to C57BL/6 J mice (Fig. EV4B,D). NSG mice are deficient in functional T cells, B cells, and NK cells (Shultz et al, 2005); thus, we employed antibody-mediated depletion to specifically evaluate the functional contribution of CD8^+^ T cells in restraining the expansion of resistant cells in orthotopic PKC-HY19636 tumors (Fig. 2M). Like NSG mice, MRTX1133 treatment of CD8^+^ T cell depleted tumors showed similar downregulation of pERK compared to CD8^+^ T cell intact tumors (Fig. EV4E,F), and increased CDK8 in CD8^+^ depleted tumors (Fig. 2N,O). Together, these data indicate that CD8^+^ T cells are critical for preventing the emergence of CDK8-expressing resistant cancer cells.

### Resistance to KRAS* inhibition is associated with reprogramming of the tumor microenvironment and lack of immunological memory response

To understand how CDK8 upregulation could alter the tumor microenvironment and potentially promote the progression of resistant tumors, spatial transcriptomics analysis and CODEX multiplex imaging of PKC-HY19636 tumors were performed. Spatial transcriptomics further revealed the distribution of stromal cells relative to ductal/cancer cells in the context of early response to MRTX1133 and MRTX1133 resistance (Appendix Figs. S6A,B and S2A–C). MRTX1133 sensitive tumors displayed reduced spatial association of ductal/cancer cells with acinar cells, endothelial cells, and Th17 cells (Appendix Fig. S6A,B). In contrast, increased association of ductal/cancer cells with memory/stem-like CD8 T cells, Th2 cells, and Tregs was observed in MRTX1133 resistant tumors (Appendix Fig. S6A,B), suggesting that resistance is associated with altered interactions between cancer cells and remodel the TME to promote immune evasion.

**Figure 3:**
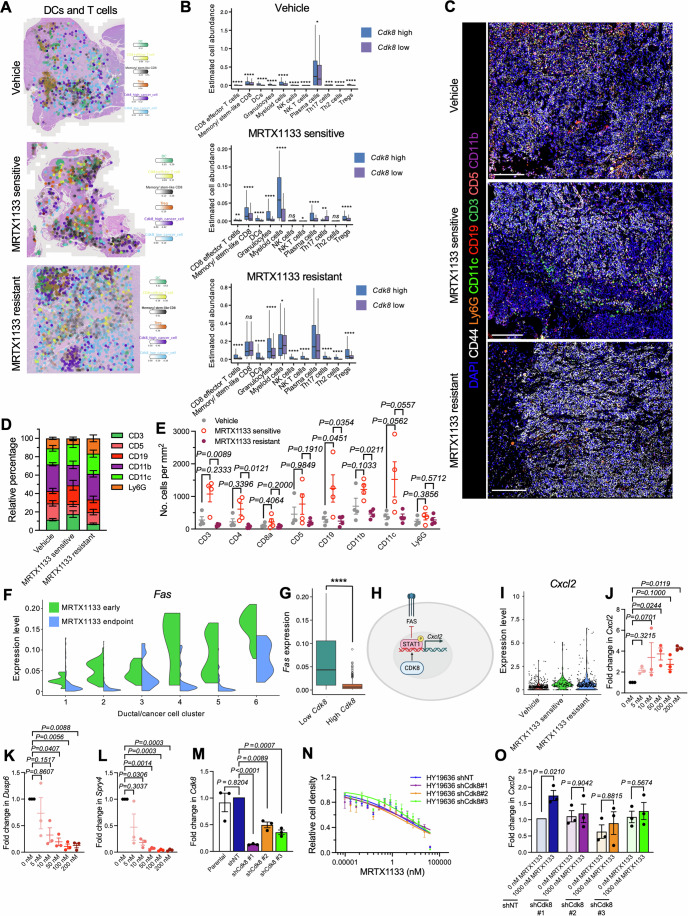
MRTX1133 resistance alters the tumor microenvironment and leads to downregulation of FAS and upregulation of CDK8 and CXCL2. (**A**, **B**) Spatial transcriptomics representative images (**A**) and quantification (**B**) of orthotopic PKC-HY19636 tumors. Spots were stratified by *Cdk8* high and low areas. Vehicle, *n* = 3; MRTX1133 sensitive, *n* = 3; MRTX1133 resistant, *n* = 3. Exact adjusted *P* values: Vehicle: CD8 effector T cells, 2.28E-17; memory/stem-like CD8, 4.58E-11; DCs, 6.00E-25; granulocytes, 4.97E-27; myeloid cells, 7.56E-09; NK cells, 2.75E-20; NK T cells, 1.24E-10; plasma cells, 3.76E-02; Th17 cells, 3.03E-04; Th2 cells, 5.98E-16; Tregs, 2.51E-05. MRTX1133 sensitive: CD8 effector T cells, 3.21E-03; memory/stem-like CD8, 5.45E-07; DCs, 1.13E-09; granulocytes, 1.52E-15; myeloid cells, 2.13E-18; NK cells, 1.85E-01; NK T cells, 2.53E-02; plasma cells, 3.15E-06; Th17 cells, 8.21E-03; Th2 cells, 5.24E-01; Tregs, 3.15E-06. MRTX1133 resistant: CD8 effector T cells, 3.54E-17; memory/stem-like CD8, 2.53E-01; DCs, 3.33E-32; granulocytes, 2.53E-10; myeloid cells, 3.15E-02; NK cells, 1.84E-24; NK T cells, 2.09E-13; plasma cells, 4.13E-04; Th17 cells, 6.24E-10; Th2 cells, 1.57E-30; Tregs, 3.27E-17. (**C**–**E**) Representative CODEX images (**C**), quantification of relative percentages of immune cell types (**D**), and number of cells per tissue area (**E**) of PKC-HY19636 orthotopic tumors. Vehicle, *n* = 4; MRTX1133 sensitive, *n* = 4; MRTX1133 resistant, *n* = 4. (**F**) *Fas* expression stratified by *Cdk8* expression level in ductal/cancer cells of MRTX1133 sensitive (early) and resistant (endpoint) orthotopic PKC-HY19636 tumors evaluated by scRNA-seq. (**G**) *Fas* expression stratified by *Cdk8* expression level in ductal/cancer cells of MRTX1133 sensitive and resistant orthotopic PKC-HY19636 tumors evaluated by scRNA-seq. The center line indicates the median, the lower and upper bounds of the box indicate the 25th and 75th percentiles, respectively, and the whiskers indicate the most extreme values within 1.5 × interquartile range. For the high *Cdk8* group, *n* = 323, minimum = 0.000536, Q1 = 0.00301, median = 0.00631, Q3 = 0.0122, maximum = 0.0874, lower whisker = 0.000536, and upper whisker = 0.0261. For the low *Cdk8* group, *n* = 323, minimum = 0.000886, Q1 = 0.0103, median = 0.0433, Q3 = 0.106, maximum = 0.208, lower whisker = 0.000886, and upper whisker = 0.208. Exact *P* value: *P* = 7.01 × 10^−41^. (**H**) Schematic of CDK8 signaling. Created in BioRender. McAndrews K (2025) https://BioRender.com/1xttcgt. (**I**) Violin plot of *Cxcl2* expression in ductal/cancer cells of PKC-HY19636 tumors. (**J**) *Cxcl2* mRNA expression in KPC689 cells treated with varying concentrations of MRTX1133. *n* = 3 independent experiments. (**K**, **L**) *Dusp6* (**K**) and *Spry4* (**L**) mRNA expression in KPC689 cells treated with varying concentrations of MRTX1133 for 6 h. Data is presented relative to *Gapdh* C_T_ values and 0 nM MRTX1133. n = 3 independent experiments. (**M**) mRNA expression of *Cdk8* in parental, shNon-targeting (shNT), and shCdk8 HY19636 cells. Data are normalized to *18 s* C_T_ values and shNT. *n* = 3 independent experiments. (**N**) Relative cell density of HY19636 cells treated with the indicated concentrations of MRTX1133 for 3 days. (**O**) mRNA expression of *Cxcl2* following MRTX1133 treatment for 24 h. Data are normalized to *18 s* C_T_ values and shNT. *n* = 3 independent experiments. Data are presented as mean ± s.e.m. except for in (**B**, **F**, **G**, **I**) where it is presented as median and range in (**B**, **G**) and violin plots in (**F**, **I**). Two-sided Mann–Whitney test was performed in (**B**). Kruskal–Wallis test with Dunn’s multiple comparisons test performed for CD3, CD4, and CD5 in (**E**) and one-way ANOVA with Dunnett’s multiple comparisons test performed for CD8, CD19, CD11b, CD11c, and Ly6G in (**E**). Scale bars, 250 μm. Wilcoxon rank-sum test performed in (**G**). One-way ANOVA with Dunnett’s multiple comparison test performed in (**J**–**M**). Unpaired two-tailed *t* test performed in (**O**). Exact *P* values reported.

*Cdk8* high cancer cells in MRTX1133 sensitive and resistant tumors were spatially associated with immune infiltrates in the TME (Fig. 3A,B). Myeloid cells, dendritic cells (DCs), granulocytes, plasma cells, and Tregs displayed increased association with *Cdk8* high cancer cells compared to *Cdk8* low cancer cells (Fig. 3A,B), indicating that KRAS* inhibitor resistant cancer cells may remodel the local tumor microenvironment and promote immunosuppression. CODEX multiplex imaging of PKC-HY19636 tumors revealed a shift to increased relative and overall abundance of CD3^+^, CD11c^+^, and CD19^+^ cells in MRTX1133 sensitive tumors (Fig. 3C–E) compared to vehicle. A paucity of T cells, B cells and putative antigen-presenting cells (APCs) were observed in MRTX1133 resistant tumors potentially indicating a lack of robust immunological memory response to KRAS* inhibition (Fig. 3C–E). To further validate the reversal of T cell infiltrates, we examined CD8^+^ T cells in the PDAC of MRTX1133 sensitive and resistant spontaneous KPCY tumors (Appendix Fig. S6C,D). Consistent with our findings from the orthotopic PKC-HY19636 tumors, the KPCY tumors demonstrated paucity of CD8^+^ T cell infiltrates in MRTX1133 resistant tumors (Appendix Fig. S6C,D). We validated alterations in the TME using an alternative resistance model, wherein in vitro parental and MRTX1133 resistant KPC689 cell lines were implanted and treated with MRTX1133 (Appendix Fig. S7A). In vitro resistant KPC689 cells were established by exposing the parental line to increasing concentrations of MRTX1133 (Fig. EV3A). The resistant and parental orthotopic tumors were treated with MRTX1133 following establishment of baseline PDAC (Appendix Fig. S7A–F). Analysis of KPC689 resistant and parental tumors demonstrate an increased frequency of myeloid cells and exhausted CD8^+^ T cells, accompanied by a decrease in B cell, CD8^+^ effector T cell, CD4^+^ T cell, and CD8^+^ memory/stem-like T cell frequencies in the MRTX1133 resistant tumors (Appendix Fig. S7G–J), suggesting a lack of a CD8^+^ T cell memory response. Taken together, our data suggests that resistance to KRAS* inhibition is associated with an upregulation of CDK8 in cancer cells and a reversal of T and B cell infiltration from the MRTX1133 sensitive tumors suggesting that CDK8 and its downstream effectors could be potential mediators of MRTX1133 resistance.

### KRAS* inhibitor resistance leads to downregulation of FAS and upregulation of CDK8 and CXCL2

Next, we probed whether CDK8 upregulation abrogates KRAS* inhibitor-mediated cell death in the PDAC. Our previous work showed that FAS expression on cancer cells induced by KRAS* inhibition promotes the induction of apoptosis through its engagement with FASL on CD8^+^ T cells (Mahadevan et al, 2023a; Mahadevan et al, 2023b). Analysis of *Fas* expression in ductal/cancer cells of PKC-HY19636 tumors by scRNA-seq revealed reduced expression in MRTX1133 resistant cancer cells (Fig. 3F). In addition, stratifying ductal/cancer cells based on *Cdk8* expression revealed an inverse association between *Cdk8* expression and *Fas* expression (Fig. 3G; Appendix Fig. S8A), suggesting a potential mechanism by which resistant cancer cells evade immune recognition and clearance.

Previous studies have demonstrated that CDK8 can inhibit expression of the death receptor FAS (Efimova et al, 2009) and induce CXCL2 expression (Chen et al, 2017) (Fig. 3H). Increased *Cxcl2*, a downstream target of CDK8 (Chen et al, 2017), was observed in ductal/cancer cells of MRTX1133 resistant tumors (Fig. 3I). Granulocytes and myeloid cells exhibit high *Cxcl2* expression which is not altered in MRTX1133 resistant tumors (Appendix Fig. S8B). Increased *Cxcl2* expression is present in acinar cells and DCs of resistant tumors (Appendix Fig. S8B), which may in conjunction with cancer cell-derived CXCL2 contribute to altered immune infiltration. Analysis of other ligands that bind to CXCR2, the cognate receptor for CXCL2, revealed minimal expression of *Cxcl1*, *Cxcl3*, and *Cxcl5* (Appendix Fig. S8C), suggesting that CXCL2 is the primary ligand produced by MRTX1133 resistant cancer cells. In vitro treatment of KPC689 cells with MRTX1133 led to upregulation of *Cxcl2* in a dose-dependent manner (Fig. 3J). In contrast, the KRAS* downstream transcriptional targets *Dusp6* and *Spry4* were inhibited at 6 h post-treatment with MRTX1133 (Fig. 3K,L), demonstrating continued suppression of KRAS* signaling. Next, to determine the functional impact of CDK8 on *Cxcl2* expression, we generated PKC-HY19636 cells with stable knockdown of *Cdk8* (Fig. 3M). Genetic suppression of *Cdk8* in PKC-HY19636 cells did not impact the proliferative response to MRTX1133 (Fig. 3N); however, the MRTX1133 treatment associated increase in *Cxcl2* expression was abrogated in shCdk8 cells (Fig. 3O). Together, these data suggest that CDK8 does not play a significant role in cancer cell proliferation in response to MRTX1133, rather impacts cancer cell extrinsic signaling, and indicate that *Cxcl2* is a downstream target of CDK8.

CXCL2 binds to CXCR2 expressed on neutrophils and myeloid cells and inhibition of CXCR2 suppresses PDAC metastasis and increases T cell infiltration to sensitize PDAC to anti-PD-1 ICB (Steele et al, 2016). Myeloid subsets of PKC-HY19636 tumors were further analyzed by scRNA-seq, where 5 myeloid clusters were defined. Cells in cluster 1 express genes associated with myeloid derived suppressor cells (MDSCs; *Arg1*, *Vegfa*, *Thbs1*, and *Spp1*), cluster 2 express genes associated with ApoE and lipid metabolism (*Apoe*, *C1q*, *Cd63*, and *Cd81*), cluster 3 express antigen presentation genes (*H2-Eb1*, *H2-Aa*, *H2-Ab1*, and *Cd74*), cluster 4 express genes associated with alternative activation (*Mrc1*, *Cd163*, and *Folr2*), and cluster 5 express proliferation genes (*Top2a*, *Mki67*, and *Stmn1*) (Appendix Fig. S8D). MRTX1133 sensitive tumors demonstrate a decrease in relative abundance of cluster 1 and an increase in the relative abundance of cluster 2, cluster 3, and cluster 4 compared to vehicle and MRTX1133 resistant tumors (Appendix Fig. S8E). Previous studies have implicated MDSCs in generating an immunosuppressive microenvironment characterized by reduced T cell infiltration and increased T cell exhaustion (Gulhati et al, 2023; Steele et al, 2016). In addition, regulatory T cells can shape the expansion and differentiation of MDSCs in the context of inflammation (Lee et al, 2016), indicating that bidirectional signaling between T cells and MDSCs may occur. This suggests a shift from predominantly myeloid cells that promote antigen presentation and anti-tumor immunity to immunosuppressive myeloid cells as resistance to MRTX1133 emerges.

### CDK8 and CXCR2 inhibition overcome KRAS* inhibitor resistance

**Figure 4:**
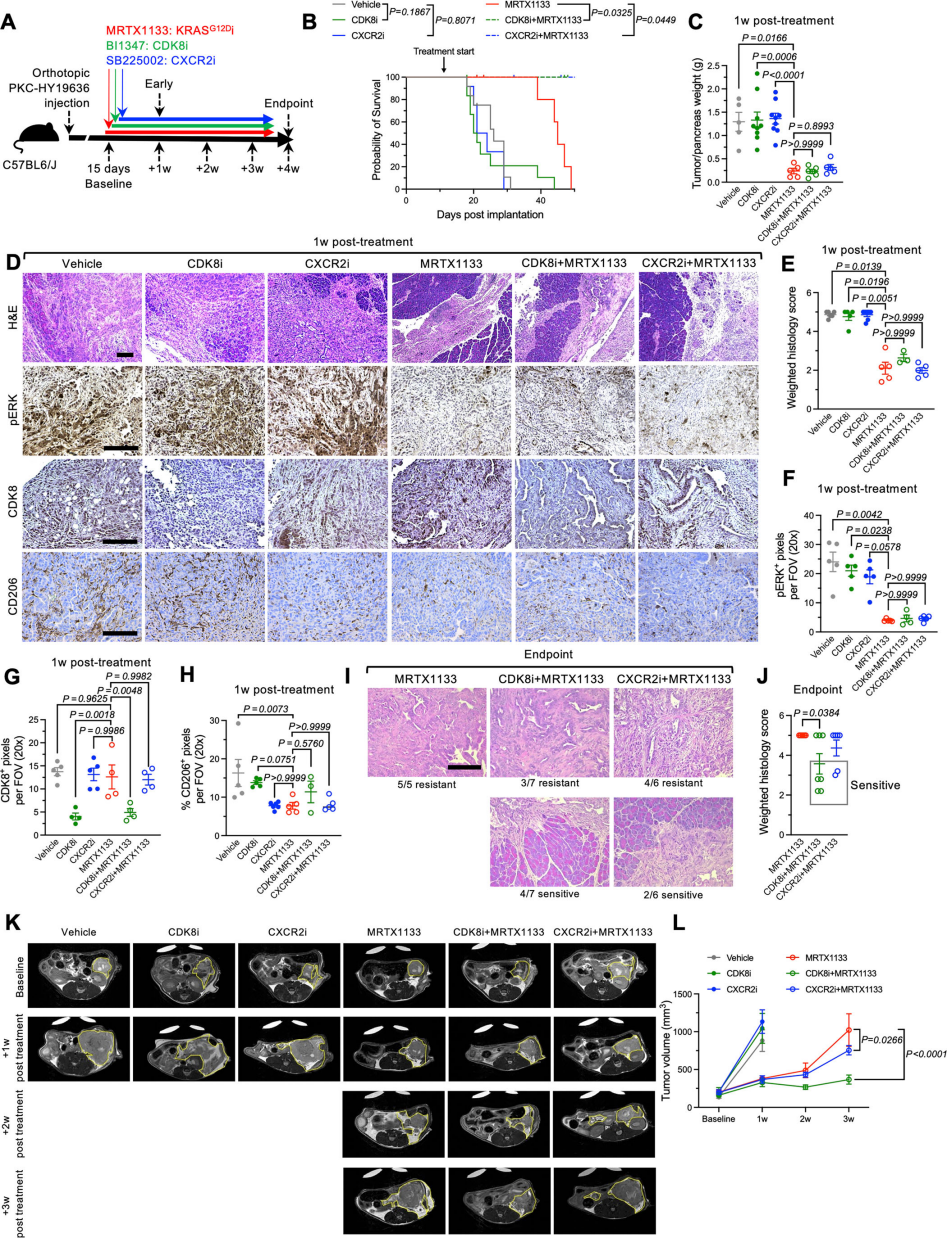
CDK8 and CXCR2 inhibition reverses MRTX1133 resistance. (**A**) Schematic of orthotopic PKC-HY19636 tumors treated with MRTX1133, CDK8i (BI1347), and CXCR2i (SB22502). (**B**) Survival curve of orthotopic PKC-HY19636 mice. Vehicle, *n* = 12; CDK8i, *n* = 12; CXCR2i, *n* = 12; MRTX1133, *n* = 11; MRTX1133 + CDK8i, *n* = 10; MRTX1133 + CXCR2i, *n* = 9. Survival experiment was terminated shortly after all the MRTX1133 treated mice reached endpoints. (**C**) Tumor/pancreas weights at 1-week post-treatment. Vehicle, *n* = 5; CDK8i, *n* = 9; CXCR2i, *n* = 9; MRTX1133, *n* = 5; MRTX1133 + CDK8i, *n* = 6; MRTX1133 + CXCR2i, *n* = 5. (**D**) Representative H&E, pERK, CDK8, and CD206 staining at 1-week post-treatment. (**E**) Weighted histology score. Vehicle, *n* = 6; CDK8i, *n* = 5; CXCR2i, *n* = 7; MRTX1133, *n* = 5; CDK8i + MRTX1133, *n* = 3; CXCR2i + MRTX1133, *n* = 5. (**F**–**H**) Quantification of pERK (**F**), CDK8 (**G**), and CD206 (**H**) staining. pERK: Vehicle, *n* = 5; CDK8i, *n* = 5; CXCR2i, *n* = 5; MRTX1133, *n* = 5; CDK8i + MRTX1133, *n *= 4; CXCR2i + MRTX1133, *n* = 5. CDK8: Vehicle, *n* = 5; CDK8i, *n* = 4; CXCR2i, *n *= 5; MRTX1133, *n* = 4; CDK8i + MRTX1133, *n* = 4; CXCR2i + MRTX1133, *n* = 4. CD206: Vehicle, *n* = 5; CDK8i, *n* = 5; CXCR2i, *n* = 6; MRTX1133, *n* = 5; CDK8i + MRTX1133, *n* = 3; CXCR2i + MRTX1133, *n* = 5. (**I**, **J**) Representative H&E (**I**) and weighted histology score (**J**) of murine pancreata at endpoint. MRTX1133, *n* = 5; CDK8i + MRTX1133, *n* = 7; CXCR2i + MRTX1133, *n* = 6. (**K**, **L**) Representative images (**K**) and quantified tumor volume measured by MRI (**L**). Yellow lines demarcate tumor regions. Vehicle, *n* = 6; CDK8i, *n* = 7; CXCR2i, *n* = 7; MRTX1133, *n* = 5; CDK8i + MRTX1133, *n* = 6; CXCR2i + MRTX1133, *n* = 7. Gehan–Breslow–Wilcoxon test performed in (**B**). Brown–Forsythe and Welch ANOVA with Dunnett’s T3 multiple comparisons test performed in (**C**). One-way ANOVA with Dunnett’s multiple comparisons test performed in (**G**, **H**). Kruskal–Wallis with Dunn’s multiple comparisons test performed in (**E**, **F**). Chi square test performed in (**J**). Two-way ANOVA with Dunnett’s multiple comparisons test performed in (**L**). Scale bars, 100 μm. Data are presented as mean ± s.e.m. Exact *P* values reported.

The observed upregulation of CDK8 and CXCL2 in KRAS* inhibitor resistant cancer cells suggested that they may be targeted to prevent resistance. Orthotopic PKC-HY19636 tumors were treated with a CDK8 inhibitor (CDK8i, BI1347) or an inhibitor of the receptor for CXCL2, CXCR2 (CXCR2i, SB225002), alone or in combination with MRTX1133 and had no impact on bodyweight during treatment (Fig. 4A; Appendix Fig. S9A). CDK8i or CXCR2i as single agents had no impact on overall survival; however, when combined with MRTX1133, a significant increase in survival was observed with both the inhibitors (Fig. 4B). At 1-week post-treatment, the weights of tumor/pancreas treated with MRTX1133 were similar to MRTX1133 combined with CDK8i or CXCR2i (Fig. 4C). Histological analysis further confirmed similar tumor burden with MRTX1133 single agent treatment compared to combination with CDK8i or CXCR2i, one-week post-treatment (Fig. 4D,E). MRTX1133 alone or in combination with CDK8i or CXCR2i was associated with reduced pERK abundance (Fig. 4D,F). In addition, decreased CDK8 was confirmed in tumors treated with CDK8i (Fig. 4D,G). CD206, a marker of myeloid derived suppressor cells (MDSCs) that express CXCR2, were significantly decreased in CXCR2i treated tumors (Fig. 4D,H). Moreover, MRTX1133 resistant tumors had increased CD206 abundance, which was abrogated with either CDK8i or CXCR2i (Appendix Fig. S9B,C). These results suggest that MRTX1133 in combination with CDK8i and CXCR2i resulted in similar tumor growth inhibition response initially and the significant survival increase associated with the combination therapy (Fig. 4B) could likely be due to prevention of resistance to MRTX1133.

To assess the impact of CDK8i and CXCR2i in preventing resistance to MRTX1133, we examined the histology of the tumors at endpoint (Fig. 4I,J). At endpoint, all the MRTX1133 treated mice (100%) developed resistance, whereas 4/7 (~ 57%) of MRTX1133 + CDK8i and 2/6 (33%) of the MRTX1133 + CXCR2i treated mice demonstrated tumor growth inhibition. MRI analysis revealed that MRTX1133 initially controlled tumor growth, but long-term treatment led to tumor relapse (Fig. 4K,L; Appendix Fig. S9D). In contrast, MRTX1133 combined with CDK8i or CXCR2i led to more durable responses, with reduced tumor burden (Fig. 4K,L; Appendix Fig. S9D). Our data demonstrates that upregulation of CDK8 functionally contributes to resistance to MRTX1133 and pharmacological targeting of CDK8 and its downstream target CXCL2 impedes the development of resistance to KRAS* inhibition in PDAC.

### Priming of KRAS* inhibitor resistant tumors with CDK8 inhibition sensitizes tumors to αCTLA-4 checkpoint blockade immunotherapy

**Figure 5:**
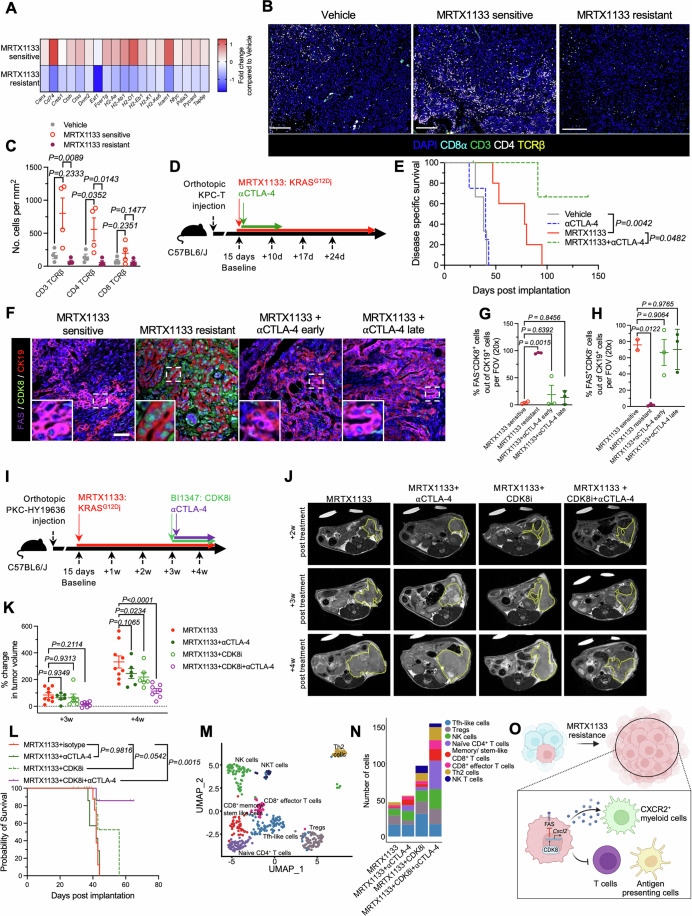
Resistance to MRTX1133 leads to loss of antigen presentation which can be reversed with inhibition of CDK8. (**A**) Expression of antigen presentation genes of ductal/cancer cells of PKC-HY19636 tumors compared to vehicle treated mice as determined by scRNA-seq analysis. (**B**, **C**) Representative CODEX images of CD3, CD8α, CD4, and TCRβ (**B**) and quantification of cell abundance per tissue area of PKC-HY19636 orthotopic tumors (**C**). Vehicle, *n* = 4; MRTX1133 sensitive, *n* = 4; MRTX1133 resistant, *n* = 4. Scale bars, 250 μm. (**D**) Schematic of orthotopic KPC-T tumors treated with MRTX1133 and αCTLA-4. (**E**) Survival curve of orthotopic KPC-T mice. Vehicle, *n* = 3; αCTLA-4, *n* = 4; MRTX1133, *n* = 5; MRTX1133 + αCTLA-4, *n* = 6. (**F**–**H**) Representative images (**F**) and quantification of FAS^-^CDK8^+^CK19^+^ (**G**) and FAS^+^CDK8^-^CK19^+^ (**H**) cells in orthotopic PKC-HY19636 tumors. MRTX1133 sensitive, *n* = 2; MRTX1133 resistant, *n* = 3; MRTX1133 + αCTLA-4 early, *n* = 3; MRTX1133 + αCTLA-4 late, *n* = 3. Scale bar, 50  μm. (**I**) Schematic of orthotopic PKC-HY19636 tumors treated with MRTX1133, CDK8i (BI1347), and αCTLA-4. (**J**, **K**) Representative images (**J**) and quantified tumor volume measured by MRI relative to tumor volume at 2 weeks following MRTX1133 treatment initiation (**K**). Yellow lines demarcate tumor regions. MRTX1133, *n* = 9; MRTX1133 + αCTLA-4, *n* = 6; MRTX1133 + CDK8i, *n* = 6; MRTX1133 + CDK8i + αCTLA-4, *n* = 7. (**L**) Survival curve of orthotopic PKC-HY19636 mice. MRTX1133, *n* = 10; MRTX1133 + αCTLA-4, *n* = 8; MRTX1133 + CDK8i, *n* = 10; MRTX1133 + CDK8i + αCTLA-4, *n* = 10. (**M**) UMAP of T and NK cells of orthotopic PKC-HY19636 tumors determined by scRNA-seq. Pancreata/tumors from three mice pooled per group. (**N**) Overall abundance of T and NK cell subsets in PKC-HY19636 determined by scRNA-seq. (**O**) Graphical abstract. Created in BioRender. McAndrews K (2025) https://BioRender.com/awmhuoy. Kruskal–Wallis with Dunn’s multiple comparisons test performed for CD3 TCRβ in (**C**), one-way ANOVA with Dunnett’s multiple comparisons test performed for CD4 TCRβ and CD8 TCRβ in (**C**), and log-rank test performed in (**E**, **L**). One-way ANOVA with Dunnett’s multiple comparisons test performed in (**G**, **H**). Two-way ANOVA with Dunnett’s multiple comparisons test performed in (**K**). *P < 0.05, **P < 0.01, ****P < 0.001, *ns*: not significant.

**Figure EV5:**
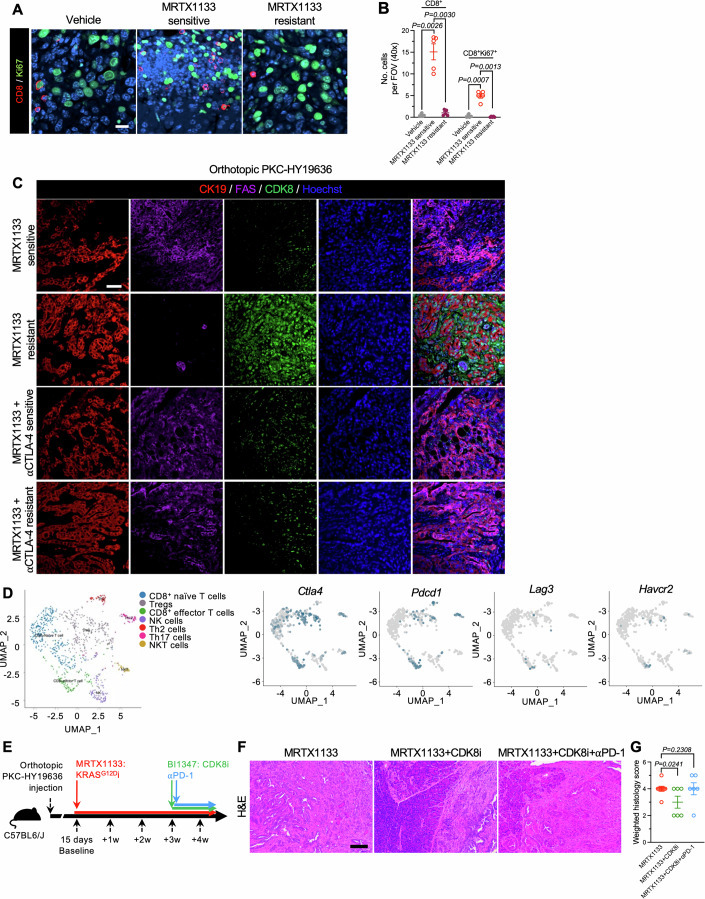
T cell responses in resistant tumors. (**A**, **B**) Representative images (**A**) and quantification of CD8^+^ and CD8^+^Ki67^+^ cells (**B**). Vehicle, *n* = 5, MRTX1133 sensitive, n = 5, MRTX1133 resistant, *n* = 5. Scale bar, 20 μm. (**C**) Single channel representative images of CK19 (red), FAS (purple), and CDK8 (green) stained orthotopic PKC-HY19636 tumors. Images from Fig. 5F are presented again here with each channel included individually. Scale bar, 50 μm. (**D**) UMAP of T cells of orthotopic PKC-HY19636 tumors and expression of checkpoint molecules determined by scRNA-seq. (**E**) Schematic of orthotopic PKC-HY19636 tumors treated with MRTX1133, CDK8i (BI1347), and αPD-1. (**F**, **G**) Representative H&E images (**F**) and quantification (**G**) of orthotopic PKC-HY19636 tumors. MRTX1133, *n* = 8; MRTX1133 + CDK8i, *n* = 6; MRTX1133 + CDK8i + αPD-1, *n* = 6. Scale bar, 100 μm. Brown–Forsythe and Welch ANOVA with Dunnett T3’s multiple comparisons test performed in (**B**). Chi-square test performed comparing responsive (score≤2) to non-responsive (score>2) in (**G**). Data are presented as mean ± s.e.m. Exact *P* values reported.

We next probed the contribution of the adaptive immune response in reversing resistance to KRAS* inhibition in combination with CDK8 inhibition and checkpoint immunotherapy. Analysis of antigen presentation related genes in the ductal/cancer cells of PKC-HY19636 tumors revealed increased expression in MRTX1133 sensitive tumors compared to vehicle; however, expression was downregulated or unchanged in MRTX1133 resistant tumors (Fig. 5A). CODEX multiplex imaging of PKC-HY19636 tumors revealed an increase in CD3^+^TCRβ^+^ and CD4^+^TCRβ^+^ cells in MRTX1133 sensitive tumors, which was reduced in MRTX1133 resistant tumors (Fig. 5B,C). Similarly, increased abundance of putative antigen presenting cells (APCs, CD11b^+^MHCII^+^ and CD11c^+^MHCII^+^) was observed in MRTX1133 sensitive tumors compared to MRTX1133 resistant tumors (Appendix Fig. S9E,F), indicating a loss of antigen presentation in the context of resistance. This finding suggested that resistant tumors would not be responsive to checkpoint blockade, and that CDK8 inhibition could potentially remodel the immune microenvironment and sensitize MRTX1133 resistant tumors to ICB. Our prior work with the orthotopic PKC-HY19636 model demonstrated that upfront combination of αCTLA-4 with the MRTX1133 therapy following establishment of advanced tumors resulted in robust tumor inhibition response (Mahadevan et al, 2023b). In a second model, MRTX1133 in upfront combination with αCTLA-4 demonstrated synergy and long-term survival in KPC-T mice (Fig. 5D,E; Appendix Fig. S9G,H). Our results establish that early CDK8 inhibition or αCTLA-4 ICB synergizes with KRAS* inhibition to impede development of resistance to MRTX1133 therapy. Further analysis of FAS expression on cancer cells and T cell proliferation during the development of MRTX1133 resistance revealed that FAS expressing cancer cells decreased in MRTX1133 resistant tumors (Fig. 5F–H). Upfront addition of αCTLA-4 resulted in a persistence of FAS^+^ CDK8^-^ cancer cells, whereas a lack of CD8^+^ T cell infiltration in the MRTX1133 resistant tumors likely resulted in an expansion of FAS^-^CDK8^+^ cancer cells (Figs. 5F–H and EV5A–C). Collectively, our results indicate that the addition of αCTLA-4 or CDK8i simultaneously with MRTX1133 prevents the development of resistance to KRAS* inhibition.

Next, to evaluate whether priming tumors with CDK8 inhibition could reverse resistance to KRAS* inhibition through eliciting T cell infiltration and sensitize tumors to ICB, PKC-HY19636 orthotopic tumor bearing mice were treated with MRTX1133 for 3 weeks to establish resistance (Fig. 5I). Treatment with αCTLA-4 and CDK8i was initiated at 3 weeks (Fig. 5I). Combination of MRTX1133 with αCTLA-4 at the time of development of resistance did not impede tumor growth likely due to the absence of T cell infiltrates in MRTX1133 resistant tumors (Figs. 5B,C,I–L and EV5A,B). Although CDK8i impeded development of resistance to MRT1133 (vide supra Fig. 4), addition of CDK8i in MRTX1133 resistant tumors resulted in a marginal improvement in survival (Fig. 5J–L). CDK8i in combination with αCTLA-4 resulted in robust inhibition of tumor growth with prolonged survival (Fig. 5J–L). Combination of CDK8i and αCTLA-4 reversed MRTX1133 resistance likely indicating that CDK8i primed the tumor microenvironment to ICB (Fig. 5M,N). Analysis of expression of immune checkpoint markers indicated that *Ctla4* is the primary immune checkpoint expressed in PKC-HY19636 tumors (Fig. EV5D). In contrast to the combination with αCTLA-4, CDK8i with αPD-1 did not significantly impact the emergence of MRTX1133 resistance (Fig. EV5E–G), potentially due to the presence of effector regulatory T cells that suppress CD8^+^ T cells (Mahadevan et al, 2025).

Further, scRNA seq analysis of the tumor infiltrating T and NK cell populations demonstrated an increase in the memory/stem-like CD8^+^ T cells, CD8^+^ effector T cells, naïve CD4^+^ T cells, and NK cells in the tumors of CDK8i and αCTLA4 combination treated mice (Fig. 5M,N; Appendix Fig. S10A). The myeloid compartment of tumors treated with MRTX1133 and CDK8i was analyzed by scRNA-seq and a decrease in the relative abundance of myeloid cells was observed (Appendix Fig. S10B,C). Further subclassification of myeloid cells revealed minor changes in the relative abundance of myeloid subpopulations (Appendix Fig. S10D,E), suggesting that CDK8 inhibition likely broadly targets myeloid cells.

Collectively, our data establishes that long-term treatment with a pharmacological inhibitor of KRAS*, MRTX1133, resulted in resistance leading to mice succumbing to PDAC. Upregulation of CDK8 in pancreatic cancer cells contributes to development of resistance to MRTX1133 therapy and inhibition of CDK8 and the downstream target CXCR2 impedes development of resistance. MRTX1133 treatment acutely increased the T cell infiltration in PDAC, rendering the tumors sensitive to αCTLA-4 ICB. However, lack of long-term immunological memory following MRTX1133 therapy resulted in paucity of T cells and APCs in resistant tumors (Fig. 5O). Although ICB did not reverse resistance to MRTX1133, combination of CDK8 inhibition rendered MRTX1133 resistant PDAC amenable to αCTLA-4 ICB.

## Discussion

Small molecule inhibitors of KRAS* have demonstrated efficacy in controlling PDAC in both preclinical models and in patients (Bekaii-Saab et al, 2023; Hallin et al, 2022; Hallin et al, 2020; Janes et al, 2018; Kemp et al, 2023; Lanman et al, 2020; Mahadevan et al, 2023b; Strickler et al, 2023); however, acquired resistance has been reported in the context of KRAS^G12C^ inhibition (Awad et al, 2021; Lv et al, 2023; Zhao et al, 2021). Previous studies have identified feedback activation of EGFR as promoting MRTX1133 resistance in pancreatic and colorectal cancer (Feng et al, 2023; Gulay et al, 2023) similar to what has been reported with KRAS^G12C^ inhibitors (Amodio et al, 2020); however, it is unclear whether resistance mechanisms unique to KRAS^G12D^ inhibition exist given the distinct impacts KRAS mutations have on pancreatic cancer initiation and progression (Zafra et al, 2020). Inherent differences in the non-covalent binding of KRAS^G12D^ inhibitors compared to covalent binding KRAS^G12C^ inhibitors may contribute to mechanisms of inhibition and emergence of resistance. KRAS^G12C^ inhibitors bind the GDP-bound inactive state (Hunter et al, 2015; Lito et al, 2016; Patricelli et al, 2016) whereas the KRAS^G12D^ inhibitor MRTX1133 is capable of binding both the inactive and active states (Hallin et al, 2022), raising the possibility that specificity of inhibitor binding may impact the mechanisms of resistance that emerge. In contrast, other small molecule inhibitors that bind to cyclophilin A to remodel its surface creating a complex with high affinity for GTP-bound active KRAS (RMC6236 (Daraxonrasib)/RMC7977 and RMC9805 (Zoldonrasib)) to inhibit downstream signaling have been developed (Wasko et al, 2024). Previous studies revealed that reactivation of RAS-MAPK signaling does not occur in the context of resistance to RMC7977, whereas *Myc* amplification is a conserved genetic event in PDAC tumors resistant to RMC7977 and MRTX1133 (Dilly et al, 2024; Wasko et al, 2024), supporting the existence of conserved and divergent resistance mechanisms across distinct types of KRAS inhibitors. Here, we identify CDK8 upregulation as a conserved response to the KRAS^G12D^ (OFF) inhibitor MRTX1133, the RAS(ON) inhibitor daraxonrasib, and the RAS^G12D^(ON) inhibitor zoldonrasib, suggesting it is agnostic to modality of RAS inhibition. While higher baseline CDK8 expression correlating with response may be indicative of its role as an inherent resistance driver, it is more likely suggestive of clonal selection of CDK8^+^ cells when KRAS^G12D^ targeting resistance is encountered resulting in overall increased CDK8 expression and functional dependency only in response to KRAS inhibitor resistance. Future studies will help clarify whether CDK8 is an inherent or acquired driver of resistance.

CRISPR screening identified *TP53*, *RB1*, and *KEAP1* as cancer cell intrinsic mediators of partial resistance to MRTX1133 (Hallin et al, 2022). Mutations in *KRAS*, *NRAS*, *MRAS*, *RET*, *MAP2K1*, *BRAF*, *NF1*, and *PTEN*, amplifications in *KRAS*^*G12C*^ and *MET*, and oncogenic fusions have also been described in tumors resistant to KRAS^G12C^ inhibitors (Awad et al, 2021; Zhao et al, 2021) and *Myc* amplification has been identified in the context of resistance to pan RAS inhibitors (Wasko et al, 2024). Moreover, feedback signaling through wildtype KRAS and RTKs can drive adaptive resistance to KRAS^G12C^ inhibition, although lineage specificity of such signaling has been reported (Amodio et al, 2020; Ryan et al, 2022). Amplifications in *Cdk6*, *Abcb1a*, *Kras*, *Yap1*, and *Myc* were identified in MRTX1133 resistant tumors in our study, and more in-depth analysis of the genomic landscape of resistant murine and patient tumors will provide critical insight into the contribution of genetic alterations to RAS inhibitor resistance and identify additional therapeutic vulnerabilities. Earlier studies have implicated YAP/TAZ, CDK4/6, and MYC as potential mediators of resistance to KRAS^G12D^ or MEK inhibition (Goodwin et al, 2023; Kapoor et al, 2014; Vaseva et al, 2018). Emergence of additional mutations has been reported in human tumors resistant to KRAS^G12C^ inhibition (Awad et al, 2021; Zhao et al, 2021) and murine KRAS^G12D^ and RAS(ON) inhibitor resistant tumors (Dilly et al, 2024; Wasko et al, 2024); however, whether such mutations functionally contribute to remodeling of the immune microenvironment is unknown. The emergence of new mutations in resistant cancer cells in combination with their suppression of CD8^+^ T cells may enable cancer cell escape from immune recognition. Indeed, ICB did not show therapeutic benefit in MRTX1133 resistant tumors, and additional priming with CDK8i was needed to sensitize MRTX1133 resistant tumors to ICB.

Here, we identify CDK8 as being amplified and upregulated in response to resistance to KRAS^G12D^ inhibitor, with a focus on its impact on the TME. CDK8 does not impact proliferative responses to MRTX1133 in murine PDAC models, rather promotes resistance in part through suppression of FAS expression and increased CXCL2 expression, which generates an immunosuppressive microenvironment. In the context of cancer, CDK8 has been shown to be an oncoprotein that promotes transcription (Donner et al, 2010; Firestein et al, 2008), suggesting that CDK8 may alter the expression of additional genes outside of *Fas* and *Cxcl2* to impact cancer cell intrinsic and extrinsic processes. Evaluation of such potential transcriptional changes induced by CDK8 may provide additional insights into the mechanisms by which it promotes resistance and will be important for future studies. It is likely that co-targeting of CXCL2/CXCR2 with CDK8i would be redundant as CXCL2 is downstream of CDK8 and redundancy in cytokine receptors (Mantovani, 1999) may explain the reduced therapeutic effectiveness of targeting CXCR2 compared to CDK8 that was observed. Based on our data, the combination of CDK8i with ICB is expected to provide more durable responses. In addition, the development of more specific CDK8 small molecule inhibitors with improved toxicity and pharmacokinetic profiles would enable clinical translation. Similar to MRTX1133, we provide evidence of CDK8 upregulation in tumors resistant to inhibitors selective for RAS(ON) and RAS^G12D^(ON): Daraxonrasib and Zoldonrasib, respectively. Future studies evaluating alternate KRAS* targeting strategies will clarify whether CDK8 upregulation is a conserved immune remodeling driven mechanism of resistance.

We demonstrate that MRTX1133 treatment initially increases the infiltration of TCRβ^+^ T cells and CD11c^+^ cells, which are lost with prolonged MRTX1133 treatment and the emergence of resistance. This suggests that there is a narrow window of opportunity to further stimulate CD8^+^ T cell mediated killing of cancer cells with ICB to effectively clear cancer cells and prevent the expansion of resistant cancer cells. Indeed, the combination of MRTX1133 with αCTLA-4 impedes the development of resistance and improves survival outcomes in mouse PDAC models (Mahadevan et al, 2023b). After CDK8-mediated resistance emerges, cancer cells likely evade immune recognition through reduced expression of FAS, which can directly engage with FASL on CD8^+^ T cells to induce cancer cell apoptosis, increased expression of CXCL2, which can signal to myeloid cells to indirectly promote suppression of T cells, and loss of antigen presentation. CXCR2 is expressed by myeloid-derived suppressor cells which can suppress T cell responses (Gabrilovich and Nagaraj, 2009; Katoh et al, 2013); thus, CXCR2 inhibition likely promotes CD8^+^ T cell activity through both FASL-dependent and independent mechanisms.

The development of small molecule inhibitors of KRAS^G12D^ has enabled targeting the dominant driver oncogene in PDAC. While KRAS^G12D^ inhibitors demonstrate control of PDAC growth, we report with long term treatment that resistance occurs. We identify CDK8 as promoting resistance through cancer cell-extrinsic signaling, inhibiting FAS expression and inducing CXCL2 secretion to reprogram the TME into an immunosuppressive state in murine models. Our studies provide a rationale for targeting CDK8 in resistant tumors and employing ICB prior to the emergence of resistance to KRAS^G12D^ small molecule inhibitors.

## Methods

**Table:** Reagents and tools table

Reagent/resource	Reference or source	Identifier or catalog number
**Experimental models**		
PKC-HY19636	Deng et al, 2021	
KPC689	Zheng et al, 2015	
KPC-T	Foley et al, 2015	
PKC-HY19636 in vivo resistant	This study	
PKC-HY19636 in vitro resistant	This study	
KPC689 in vitro resistant	This study	
C57BL/6 J mice	Jackson Laboratory	Cat# 000664
*LSL-Kras*^*G12D/+*^*; LSL-Trp53*^*R172H/+*^*; Pdx1-Cre; LSL-EYFP* (KPCY) mice	Hingorani et al, 2003, 2005; Srinivas et al, 2001	
Athymic nude mice	Charles River Laboratories	Crl:NU(NCr)-*Foxn1*^*nu*^
Patient derived xenografts	Carugo et al, 2016	
**Antibodies**		
InVivoMAb anti-mouse CTLA-4 (CD152)	BioXcell	Cat# BE0131
InVivoMAb anti-mouse PD-1 (CD279)	BioXcell	Cat# BE0273
InVivoMAb polyclonal Syrian hamster IgG	BioXcell	Cat# BE0087
InVivoMAb rat IgG2a isotype control, anti-trinitrophenol	BioXcell	Cat# BE0089
Phospho-p44/42 MAPK (Erk1/2) (Thr202/Tyr204) (20G11) Rabbit Monoclonal Antibody	Cell Signaling	Cat# 4376
Mouse MMR/CD206 Goat Monoclonal Antibody	R&D Systems	Cat# AF2535
Anti-GFP Rabbit Polyclonal Antibody	Abcam	Cat# ab290
CDK8 Rabbit Polyclonal Antibody	Proteintech	Cat# 22067-1-AP
Anti-Cytokeratin 19 Rabbit Monoclonal Antibody [EP1580Y]	Abcam	Cat# ab52625
Goat Anti-Rabbit IgG Antibody (H + L), Biotinylated	Vector Biolaboratories	Cat# BA-1000
Biotin-SP (long spacer) AffiniPure® F(ab’)₂ Fragment Donkey Anti-Goat IgG (H + L)	Jackson ImmunoResearch	Cat# 705-066-147
CD8 alpha (D4W2Z) Rabbit Monoclonal Antibody	Cell Signaling	Cat# 98941
Ki-67 Rabbit Monoclonal Antibody	Thermo Fisher Scientific	Cat# RM-9106-S
4+ Biotinylated Goat Anti-Rabbit IgG	Biocare Medical	Cat# GM601
Anti-Hu/Mu CD44(AKYP0003)-BX005 – Atto 550	Akoya Biosciences	Cat# 4250002
Anti-Mu CD3(AKYP0035)-BX021 – Alexa Fluor™ 647	Akoya Biosciences	Cat# 4550109
Anti-Mu CD4(AKYP0041)-BX026 – Atto 550	Akoya Biosciences	Cat# 4250016
Anti-Mu CD5(AKYP0016)-BX017 – Atto 550	Akoya Biosciences	Cat# 4250007
Anti-Mu CD8a(AKYP0044)-BX029 – Atto 550	Akoya Biosciences	Cat# 4250017
Anti-Mu CD11b(AKYP0040)-BX025 – Alexa Fluor™ 488	Akoya Biosciences	Cat# 4150015
Anti-Mu CD11c(AKYP0045)-BX030 – Alexa Fluor™ 647	Akoya Biosciences	Cat# 4550108
Anti-Mu CD19(AKYP0033)-BX020 – Atto 550	Akoya Biosciences	Cat# 4250014
Anti-Mu Ly6g(AKYP0039)-BX024 – Alexa Fluor™ 647	Akoya Biosciences	Cat# 4550110
Anti-Mu TCRb(AKYP0011)-BX003 – Alexa Fluor™ 647	Akoya Biosciences	Cat# 4550101
Anti-Mu MHC II(AKYP0006)-BX014 – Atto 550	Akoya Biosciences	Cat# 4250003
Ras (G12D Mutant Specific) (D8H7) Rabbit Monoclonal Antibody	Cell Signaling Technology	Cat# 14429
Phospho-p44/42 MAPK (Erk1/2) (Thr202/Tyr204) (D13.14.4E) Rabbit Monoclonal Antibody	Cell Signaling Technology	Cat# 4370
p44/42 MAPK (Erk1/2) Rabbit Polyclonal Antibody	Cell Signaling Technology	Cat# 9102
Anti-Vinculin Rabbit Monoclonal Antibody [EPR8185]	Abcam	Cat# ab129002
Donkey Anti-Rabbit IgG H&L (HRP)	Abcam	Cat# ab16284
**Oligonucleotides and other sequence-based reagents**		
shCdk8_1	Sigma-Aldrich	Table 1
shCdk8_2	Sigma-Aldrich	Table 1
shCdk8_3	Sigma-Aldrich	Table 1
qPCR primers	This study	Table 4
*KRAS*^*G12D*^ TaqMan dPCR Liquid Biopsy Assay	Applied Biosystems	Cat# A44177, Hs000000051
**Chemicals, enzymes and other reagents**		
Collagenase IV	Gibco	Cat# 17104019
Dispase	Gibco	Cat# 17105041
MRTX1133	WuXi AppTech	
F-Luc-GFP Lentivirus	Capital Biosciences	Cat# VSL-0088P
pLKO.1-puro Non-Mammalian shRNA Control Transduction Particles	Sigma-Aldrich	Cat# SHC002VN
D-luciferin	Goldbio	Cat# LUCK-1G
Captisol	Selleck Chemicals	Cat# S4592
Citrate buffer	Teknova	Cat# S8450
BI1347	MedChem Express	Cat# HY-120350
SB225002	Selleck Chemicals	Cat# S7651
RMC6236 (daraxonrasib)	TargetMol	Cat#: T-74698; CAS#: 2765081-21-6
DMSO	MP Biomedicals	Cat# 0219605590
PEG-400	Nanocs	Cat# PEG-400
Solutol HS15	Selleck Chemicals	Cat# E1708
RMC9805	MedChem Express	Cat#: HY-156819, CAS#: 2922732-54-3
ABC reagent	Vector Laboratories	Cat# PK-6100
Rabbit on rodent HRP polymer	Biocare Medical	Cat# RMR622
Opal 520	Akoya Biosciences	Cat# FP1487001KT
Opal 570	Akoya Biosciences	Cat# FP1488001KT
Opal 690	Akoya Biosciences	Cat# FP1497001KT
Hoechst 33342	Invitrogen	Cat# H3570
VECTASHIELD Antifade Mounting Medium	Vector Laboratories	Cat# H-1000
DAB	Life Technologies	Cat# 750118
Fixable Viability Dye eFluor 780	eBioscience	Cat# 65-0865-14
cOmplete mini EDTA-free protease inhibitor cocktail	Roche	Cat# 11836170001
**Software**		
LAS version 4.4 software	Leica	
ImageJ	https://imagej.net/ij/ NIH	
QuPath v0.5.1	https://qupath.github.io/ (Bankhead et al, 2017)	
GSEA 4.2.2	https://www.gsea-msigdb.org/gsea/index.jsp (Mootha et al, 2003; Subramanian et al, 2005)	
Space Ranger 2.0.1	10x Genomics	
R Studio	https://posit.co/products/open-source/rstudio	
Compass for SW	Bio-Techne	
QuantStudio 3D Analysis Suite software	Applied Biosystems	
**Other**		
Illumina NovaSeq6000	Illumina	
10x Chromium Controller	10x Genomics	
Chromium Next GEM Single Cell 3’ Reagents Kits v3.1	10x Genomics	
Visium CytAssist Spatial Gene Expression for FFPE, Mouse Transcriptome kit	10x Genomics	
Illumina NextSeq500	Illumina	
Illumina NextSeq2000	Illumina	
RNeasy kit	Qiagen	Cat# 74104
High-Capacity cDNA Reverse Transcription kit	Applied Biosystems	Cat# 4368814
Power SYBR Green PCR Master Mix	Applied Biosystems	Cat# 4367659
Applied Biosystems QuantStudio 7 Flex Real-Time PCR System	Applied Biosystems	
Pierce™ BCA Protein Assay Kits	Thermo Scientific	Cat# 23227
Anti-rabbit detection module	Bio-Techne	Cat# DM-001
Wes instrument	Bio-Techne	
CellTiter-Glo® Luminescent Cell Viability Assay	Promega	Cat# G7570
DNeasy Blood and Tissue kit	Qiagen	Cat# 69504
QuantStudio 3D Digital PCR Master Mix v2	Applied Biosystems	Cat# A26358
QuantStudio 3D Digital PCR 20 K chips	Applied Biosystems	Cat# A26316
Twist Mouse Exome Panel	Twist Biosciences	Cat# 102035

### Cell culture

PKC-HY19636 were isolated from *Ptf1a-Cre; LSL-Kras*^*G12D/+*^*; Trp53*^*F/+*^ (PKC) tumors (Deng et al, 2021) and KPC689 (Zheng et al, 2015) and KPC-T (Foley et al, 2015) were isolated from *Pdx1-Cre; LSL-Kras*^*G12D/+*^*; LSL-Trp53*^*R172H/+*^ (KPC) tumors as previously described. PKC-HY19636 in vivo resistant cells were isolated from a male mouse with an orthotopic HY19636 tumor that developed resistance to MRTX1133 (treated for 39 days). The tumor was digested in 4 mg/mL collagenase IV (Gibco) and 4 mg/mL dispase (Gibco) in RPMI with 1% PSA for 50 min at 37 °C with shaking, followed by consecutive filtering through 70 μm and 40 μm filters. Cells were washed with growth media: RPMI (Corning) with 10% FBS (Avantor) and 1% penicillin–streptomycin with antimycotics (PSA, Corning), centrifuged at 500×*g* for 5 min, then seeded on collagen I coated plates (Sigma-Aldrich) in growth media. KPC689 and HY19636 in vitro resistant cells were generated by exposing KPC689 parental and HY19636 parental cells to increasing concentrations of MRTX1133 (WuXi AppTech) over the course of 2 to 3 months: 150 nM MRTX1133, followed by 300 nM MRTX1133, 600 nM MRTX1133, and 900 nM MRTX1133. In vitro resistant cells were then maintained in 900 nM MRTX1133 unless noted otherwise. KPC689 were transduced with a GFP-luciferase lentivirus (Capital Biosciences) followed by selection with and maintenance in 2 μg/mL puromycin (Sigma-Aldrich). HY19636 cells were transduced with non-targeting shRNA lentiviral particles (shNT, pLKO.1-puro Non-Mammalian shRNA Control Transduction Particles, Sigma-Aldrich SHC002VN) or shCdk8 lentiviral particles (Sigma-Aldrich SHCLNV, Table 1) with 8 μg/mL polybrene (Millipore) followed by selection with and maintenance in 8 μg/mL puromycin (Sigma-Aldrich).

**Table 1:** shCdk8 clones and target sequences.

shRNA	Clone ID	Target sequence
shCdk8_1	TRCN0000023107	CCTGATGAGAAAGGAGACAAA
shCdk8_2	TRCN0000023104	GCAATTGATATTTGGGCTATA
shCdk8_3	TRCN0000023105	GCAATTGATATTTGGGCTATA

Cells were cultured at 37 °C and 5% CO_2_ in the media listed in Table 2. All cell lines were routinely tested for mycoplasma and confirmed negative with LookOut Mycoplasma PCR Detection Kit (Sigma-Aldrich).

**Table 2:** Cell line origins and culture conditions.

Cell line	Origin	Culture media
HY19636	Deng et al, 2021	RPMI (Corning), 10% FBS, 1% penicillin–streptomycin (PS, Corning)
KPC689	Zheng et al, 2015	RPMI, 10% FBS, 1% PS
KPC-T	Foley et al, 2015	RPMI, 10% FBS, 1% PS
HY19636 in vivo resistant	This manuscript	RPMI, 10% FBS, 1% PSA
KPC689 in vitro resistant	This manuscript	RPMI, 10% FBS, 1% PS, 900 nM MRTX1133
HY19636 in vitro resistant	This manuscript	RPMI, 10% FBS, 1% PS, 900 nM MRTX1133

### Animal studies

Mice were housed at the MDACC animal facility and animals received LabDiet 5053 chow and sterile water ad libitum and were group housed with a 12-h-light/dark cycle at 21–23 °C and 40–60% humidity. Male and female 8- to 16-week-old C57BL6/J mice were purchased from Jackson Laboratory. In total, 5 × 10^5^ PKC-HY19636, KPC689 GFP-luciferase, KPC689 parental, KPC689 in vitro resistant, or KPC-T cells were orthotopically injected into the pancreas tail with a 27-gauge Hamilton syringe. PDX experiments were previously described (Mahadevan et al, 2023b). Spontaneous *LSL-Kras*^*G12D/+*^*; LSL-Trp53*^*R172H/+*^*; Pdx1-Cre; LSL-EYFP* (KPCY) mice (Hingorani et al, 2003; Hingorani et al, 2005; Srinivas et al, 2001) were generated, maintained on mixed background, and enrolled in treatment in a randomized manner at approximately 85 days when the presence of tumors was confirmed by MRI. *LSL-Kras*^*G12D/+*^*; Pdx1-Cre* (KC) were generated, maintained on mixed background, and enrolled in treatment in a randomized manner at approximately 49 days. For orthotopic experiments, treatment was initiated at the timepoints indicated in the figures. Imaging of KPC689 GFP-luciferase tumors was performed with IVIS (Xenogen Spectrum) following injection of D-luciferin (Goldbio) according to the manufacturer’s instructions. The tumor size of PKC-HY19636, KPC689 parental, KPC689 in vitro resistant, and KPC-T orthotopic tumors and KPC GEMM tumors was monitored with a 7 T Bruker MRI at the MDACC Small Animal Imaging Facility (SAIF). Treatment started at the indicated times post-implantation and consisted of vehicle, 10% Captisol (Selleck) in 50 mM citrate buffer pH 5.0 (Teknova), 30 mg/kg MRTX1133 (WuXi AppTech) in vehicle, or 60 mg/kg MRTX1133 in vehicle (dose escalation) injected BID i.p. BI1347 (CDK8i, 10 mg/kg, MedChem Express) was administered QD by oral gavage and SB225002 (CXCR2i, 10 mg/kg, Selleck Chemicals) was administered QD i.p. in 10% Captisol in 50 mM citrate buffer pH 5.0. Anti-CTLA-4 (BioXcell, BE0131), anti-PD-1 (BioXcell, BE0273) or the respective isotype (BioXcell, BE0087 for anti-CTLA-4; BioXcell, BE0089 for anti-PD-1) in PBS were administered three times per week i.p. once at a dose of 200 μg, followed by two doses of 100 μg. For RMC6236 (Daraxonrasib) orthotopic experiments, vehicle consisted of 10% DMSO (MP Biomedicals), 20% PEG-400 (Nanocs), 10% Solutol HS15 (Selleck Chem), and 60% milliQ water. RMC6236 in vehicle was administered at 25 mg/kg QD by oral gavage. CD8 depletion experiments were previously described (Mahadevan et al, 2023b). Control arms for inhibitor experiments were administered vehicle and/or isotype antibody. PDXs were derived as previously described (Carugo et al, 2016) and implanted subcutaneously into 11 weeks old athymic nude mice. Tumor volumes were captured by serial caliper measurements (twice weekly) and treatment was initiated when tumors reached 150–250 mm^3^. Tumor volume (TV) was calculated as TV = (D × d^2^/2), where “D” is the larger and “d” is the smaller superficial visible diameter of the tumor mass. All measurements were documented as mm^3^. Treatment groups included 6 mice per group. Tumors were implanted and allowed to reach approximately 150–250 mm^3^ and dosed with RMC9805 (zoldonrasib) at 100 mg/kg QDx7, 10 mL/kg by oral gavage. To generate PDXs resistant to RMC6236 (Daraxonrasib), tumors were implanted and allowed to reach approximately 400–500 mm^3^ and dosed with RMC6236 at 25 mg/kg QDx7 by oral gavage until tumors reached ~1000 mm^3^ in size. At the endpoint of the study, tumors were collected 4 h after the last dose of treatment and fixed in 10% neutral buffered formalin for 48 h and then processed and embedded in paraffin. FFPE tissues were sectioned at 5 µm for histology analyses. CDK8 immunostaining was performed on tumors at the following timepoints: PATX66, 23 days; PATX69, 46 days; PATX155, 42 days; PATX90, 39 days. All procedures were reviewed and approved by the MD Anderson Institutional Animal Care and Use Committee (protocols 00001251-RN03 and 00000884-RN04) and data reported according to ARRIVE guidelines.

### Histological analysis

In all, 5 μm-thick sections of formalin-fixed paraffin-embedded (FFPE) tissues were stained with hematoxylin and eosin (H&E) with the S&T Infinity Staining and Leica Autostainer XL. Representative pictures were acquired with a Leica DM1000 LED microscope equipped with a DFC295 microscope camera (Leica) with LAS version 4.4 software (Leica). Five fields of view at ×100 magnification per tissue were analyzed for histological scoring. For histological scoring of KPC689 GFP-luciferase and αPD-1 treated tumors, H&E stained slides were scanned with a Zeiss Axio Scan.Z1 and scoring performed based on the entire tissue. Orthotopic models will have either PDAC or uninvolved tissue and were scored based on the percentage of the tissue that consisted of PDAC (1: no PDAC, 2: 0–25% PDAC, 3: 26–50% PDAC, 4: 51–75% PDAC, 5: 76–100% PDAC). Tissues from GEMMs were quantified based on the relative percentage of tissue with each indicated histological phenotype.

### Immunohistochemistry

For IHC staining, 5 μm formalin-fixed FFPE tissues were processed as reported previously (Mahadevan et al, 2023b). Antigen retrieval was carried out by subjecting the sections to Tris-EDTA (TE) buffer (pH 9.0) for pERK, CD206, and GFP after deparaffinization and hydration. For CDK8 and CK19, antigen retrieval was performed with citrate buffer (pH 6.0). A hydrophobic barrier was then created around the tissue sections followed by the incubation of 3% H_2_O_2_ in PBS for 15 min to block endogenous peroxidase activity. The slides were blocked with 1.5% bovine serum albumin for 30–60 min prior to incubation with anti-pERK (Cell Signaling 4376S, 1:250), CDK8 (Proteintech 22067-1-AP, 1:100), GFP (Abcam ab290, 1:400), or CK19 (Abcam ab52625, 1:400) overnight at 4 °C. For CD206 staining, blocking was performed in 4% cold water fish skin gelatin (CWFG, Aurion 14719NT) for 1 h and staining with anti-CD206 (R&D Systems AF2535, 1:100) overnight at 4 °C. The slides were incubated with biotinylated anti-rabbit secondary antibody (Vector Laboratories BA-1000, 1:200-1:250) or biotinylated anti-goat secondary antibody (Jackson ImmunoResearch 705-066-147, 1:250) for 30 min in blocking buffer followed by incubation with ABC reagent (Vector Laboratories PK-6100) for 30 min. Subsequently, the slides were stained with DAB (Life Technologies), counterstained with hematoxylin, and cover slipped. Representative pictures were acquired with a Leica DM1000 LED microscope equipped with a DFC295 microscope camera with LAS version 4.4 software. At least three random fields for each tissue section were captured, and the staining per visual field (×200) was quantified with ImageJ (NIH, Bethesda, MD). Quantification of CDK8 stained tissues from daraxonrasib and zoldonrasib experiments was performed in QuPath v0.5.1 (Bankhead et al, 2017). Weighted IHC scoring was performed based on positive area (0: 0%, 1: 1–10%, 2: 11–50%, 3: 51–80%, 4: 81–100%) and intensity (0: negative, 1: weak, 2: moderate, 3: strong).

For the visualization of CK19, FAS, and CDK8 co-expression, tyramide signal amplification (TSA) multiplex staining was performed. In all, 5 μm FFPE tumor tissues were deparaffinized and hydrated, and antigen retrieval was performed in citrate buffer (pH 6.0) for 15 min at 98 °C in a microwave. Antigen retrieval for CK19 on PDX samples was performed in TE buffer. Subsequently, a hydrophobic barrier was then created around the tissue sections before blocking with 4% CWFG for 1 h at room temperature (RT), followed by the incubation with CK19 antibody (Abcam ab52625, 1:200) for 3 h at RT. Then the slides were incubated with rabbit on rodent HRP-Polymer (Biocare Medical) for 30 min at RT followed by staining with Opal 570 (Akoya Biosciences) for 10 min at RT. Then the slides were heated at 98 °C in citrate buffer (pH 6.0) for 15 min in a microwave again followed by the blocking with 4% CWFG for 1 h at RT, incubated with FAS antibody (Abcam ab271016, 1:100) for 3 h at RT, incubated with rabbit on rodent HRP-Polymer for 30 min at RT and Opal 690 (Akoya Biosciences) for 10 min at RT. Afterwards, the slides were heated at 98 °C in citrate buffer (pH 6.0) for 15 min in a microwave followed by the blocking with 4% CWFG for 1 h at RT, incubated with CDK8 (Proteintech 22067-1-AP, 1:100) for 3 h at RT, 4plus Biotinylated Goat Anti-Rabbit IgG (Biocare Medical) for 30 min and 4plus Streptavidin HRP Label (Biocare Medical) for 30 min at RT, stained with Opal 520 (Akoya Biosciences) for 10 min before the incubation with Hoechst 33342 (Invitrogen, 1:10,000) for 10 min and mounted with Vectashield (Vector Laboratories). A similar process was performed for CD8, Ki67, pERK, and CK19 staining, with antigen retrieval steps performed in TE buffer (pH 9.0) in a microwave at 98 °C for 15 min and blocking in 1% BSA for 10 min, followed by either anti-CD8 (Cell Signaling 98941S, 1:800), anti-Ki67 (Thermo Fisher Scientific RM-9106-S, 1:400), pERK (Cell Signaling 4376, 1:500), or CK19 (Abcam ab52625, 1:400) incubation for 1 h at room temperature or overnight at 4 °C. Tissues were incubated with BioCare Rabbit-on-Rodent HRP polymer for 10 min at room temperature, and Opal 570 (for CD8 and pERK, 1:100) or Opal 520 (for Ki67 and CK19, 1:100) for 10 min at room temperature. Images were captured with a Zeiss LSM800 confocal microscope with a ×20 objective and quantified by counting the number of positive cells in each visual field. For CK19 and pERK imaging, images were captured with 2× digital zoom (×40).

### PhenoCycler staining and analysis

Tissues were embedded in OCT and cryostat sectioned (5 μm) onto coverslips coated with 1× poly-L-lysine. For tissue retrieval, sample coverslips were placed over Drierite beads for 2 min before being immersed in acetone for 10 min and then placed in a humidity chamber for 2 min. Next, tissues were hydrated (two 2-min incubations in Hydration Buffer, Akoya Biosciences), fixed [10 min, 1.6% paraformaldehyde (PFA) in hydration buffer solution], and rinsed in Hydration Buffer prior to staining.

For staining, tissues were first incubated for 20–30 min in Staining Buffer (Akoya Biosciences) before adding the CODEX antibody cocktail [CODEX antibodies (see Table 3 for list of antibodies used) in CODEX Blocking Buffer, Akoya Biosciences] to coverslips and incubation in a humidity chamber at room temperature for 3 h. Following the 3-hour antibody incubation, tissues were washed (2 times for 2 min) in Staining Buffer (Akoya Biosciences) before a secondary post-stain fixation step (10 min, 1.6% PFA in Storage Buffer, Akoya Biosciences). Next, tissues were washed (3 times with 1× PBS), incubated in ice-cold methanol for 5 min, and washed (3 times with 1× PBS) prior to a final fixation step in a humidity chamber [20 min at room temperature with Fixative Reagent (Akoya Biosciences) in 1× PBS]. Lastly, samples were washed 3 times in 1× PBS and placed in Storage Buffer (Akoya Biosciences) at 4 °C.

**Table 3:** Antibodies used for PhenoCycler analysis.

Antibody	Catalog #	Clone	Dilution
CD44	4250002	IM7	1:375
CD3	4550109	17A2	1:200
CD4	4250016	RM4-5	1:200
CD5	4250007	53-7.3	1:200
CD8a	4250017	53-6.7	1:200
CD11b	4150015	M1/70	1:200
CD11c	4550108	N418	1:200
CD19	4250014	6D5	1:200
Ly6G	4550110	1A8	1:200
TCRβ	4550101	H57-597	1:200
MHCII	4250003	M5/114.15.2	1:200

Stained coverslips were run on the PhenoCycler System (Akoya Biosciences) to obtain stitched ×20 images (Keyence, BZ-X800) of barcoded reporters. Images were analyzed using QuPath (Bankhead et al, 2017). Classifiers for markers were trained by researchers blinded to sample group and applied to images to quantify individual staining as well as co-expression of markers. Raw counts were then normalized to tissue area (mm^2^).

### scRNA-seq sample preparation

Tissue digestion and scRNA-seq preparation was performed as previously described (Mahadevan et al, 2023b). Briefly, 2–3 pooled tumors per condition were minced with a razor and digested with 4 mg/mL collagenase IV and 4 mg/mL dispase in RPMI with 1% PSA for 50 min at 37 °C with shaking, followed by consecutive filtering through 70 μm and 40 μm filters. Samples were washed with RPMI with 10% FBS and 1% PSA, centrifuged at 500× *g* for 5 min, washed with FACS buffer (PBS with 2% FBS), centrifuged at 500× *g* for 5 min, and stained with Fixable Viability Dye eFluor™ 780 (eBioscience, 1:1000) on ice for 10 min. Samples were washed twice with washed with FACS buffer, centrifuged at 500× *g* for 5 min, and sorted with a BD FACSMelody. Cell counting was performed with Trypan Blue (Sigma-Aldrich) exclusion with a Countess 3 (Invitrogen). In total, 5000 to 10,000 cells were loaded into the 10x Chromium Controller to generate Single Cell 3’ Gene Expression dual indexed libraries (10x Genomics). The creation of Gel Bead-In-Emulsions (GEM) and subsequent cell barcoding, GEM-RT cleanup, cDNA amplification, and library assembly were performed with the Chromium Next GEM Single Cell 3’ Reagents Kits v3.1 (10x Genomics) according to manufacturer’s instructions. Paired-end, dual indexing sequencing was carried out using the Illumina NovaSeq6000.

### scRNA-seq data processing and analysis

scRNA-seq data processing and analysis was performed as previously described (Mahadevan et al, 2023b). Gene imputation was conducted by Markov affinity-based graph imputation of cells (MAGIC) (van Dijk et al, 2018). Seurat employs a global-scaling normalization method “LogNormalize” that normalizes the feature expression measurements for each cell by the total expression, multiplies this by a scale factor (10,000 by default), and log-transforms the result. To have a comparable number of cells for all samples included in the same comparison analysis, we downsampled the HY19636 MRTX1133 + CDK8i + αCTLA-4 sample by randomly selecting 1400 cells from the original dataset using the R function “sample”. We also downsampled the orthotopic KPC689 resistant MRTX1133 sample to 3300 cells to have a similar number of cells to KPC689 parental. GSEA was performed with GSEA 4.2.2 (Mootha et al, 2003; Subramanian et al, 2005) based on differentially expressed genes for each cluster and pathways with an FDR *q* value < 0.1 reported.

### Visium spatial transcriptomics analysis

H&E stained 5-μm-thick FFPE tissues were scanned with a Zeiss Axio Scan.Z1 followed by processing with the Visium CytAssist Spatial Gene Expression for FFPE, Mouse Transcriptome kit (10x Genomics) according to manufacturer’s instructions. Prepared libraries were sequenced with a NextSeq500 (Illumina), NextSeq2000 (Illumina), or NovaSeq6000 (Illumina) according to manufacturer’s recommendations.

Raw FASTQ files were mapped to the mm10 reference genome and spatially projected using Space Ranger 2.0.1 (10x Genomics). Cell2location (version 0.1.3) (Kleshchevnikov et al, 2022) was employed to create spatial maps of cell types by integrating Visium spatial data and scRNA-seq references of the matched samples. Cell2location decomposes multi-cell spatial transcriptomics data by estimating the abundance of reference cell types at specific spatial coordinates using a principled Bayesian model. Our analysis includes multiple steps. First, a high-quality reference of cell annotations was constructed using the scRNA-seq data of matched mouse samples and fed into cell2location as input. Second, cell2location performed gene selection and defined expression signatures for each cell type. Subsequently, the default negative binomial regression model in cell2location was utilized to estimate the reference cell-type signatures for determining cell-type locations. For the analysis of each Visium section, the default parameters were applied, with the exception of the following settings: ‘max_spochs’: 50,000; ‘N_cells_per_location’: 5; ‘detection_alpha’: 20. To visually represent the contribution of each cell type to each spot, the 5% percentile of the posterior distribution of mRNA counts was employed. The gene expressions of each cell type at each spot were also estimated using cell2location with the adopted approach from robust cell type decomposition (RCTD) (Cable et al, 2022). The estimated gene expressions of each cell type were exported and the spots stratified based on the mean *Cdk8* expressions in ductal/cancer cells by excluding the spots with zeros. The expression and cell proportion matrices were concatenated for each spot and the combined matrix input back to cell2location for visualization.

### RNA-seq analysis

Data derived from previously published RNA-seq data from Panc02.03 and PANC-1 parental and MRTX1133 resistant cells and PDXs treated with MRTX1133 (Dilly et al, 2024) was analyzed for *CDK8* expression. For resistant human cell lines, *CDK8* fragments per kilobase of transcript per million mapped fragments (FPKM) were downloaded from Gene Expression Omnibus (GSE269985) and plotted.

### In vitro MRTX1133 treatment

For in vitro experiments with MRTX1133 treatment, 5 × 10^5^–1 × 10^6^ cells were seeded in one well of a six-well plate. 24 h later, cells were treated with vehicle (DMSO) or the indicated concentrations of MRTX1133 in DMSO. RNA was collected at 6- or 24-h post-treatment according to the RNeasy kit protocol (Qiagen). cDNA synthesis was performed with the High-Capacity cDNA Reverse Transcription kit (Applied Biosystems) and qPCR performed with Power SYBR Green PCR Master Mix and the primers listed in Table 4 and analyzed with the Applied Biosystems QuantStudio 7 Flex Real-Time PCR System (Applied Biosystems). Gene expression is calculated using the 2^-ΔΔCT^ method with *Gadph* or *18 s* as a housekeeping gene and normalized to vehicle or shNT as indicated in the figure legends.

**Table 4:** qPCR primer sequences.

Gene	Forward primer sequence	Reverse primer sequence
*18 s*	5’-GTAACCCGTTGAACCCCATT-3’	5’-CCATCCAATCGGTAGTAGCG-3’
*Gapdh*	5’-AGGTCGGTGTGAACGGATTT-3’	5’-TGTAGACCATGTAGTTGAGGTCA-3’
*Dusp6*	5’-GTTCTACCTGGAAGGTGGCTT-3’	5’-TGTCCGAGGAAGAGTCCGAG-3’
*Spry4*	5’-TCGGGTTCGGGGATTTACAC-3’	5’-ACAGGCTTCTAGGGGTCTTTG-3’
*Cxcl2*	5’-CCCAGACAGAAGTCATAGCCAC-3’	5’-TGGTTCTTCCGTTGAGGGAC-3’
*Cdk8*	5’-AGAAAGGAGACAAAACCCAGCA-3’	5’-CAGCATGTGGATTGGAACGC-3’

Protein was collected at 3 h post-treatment in urea lysis buffer (8 M urea, 2.5% SDS) with cOmplete mini EDTA-free protease inhibitor cocktail (Roche) and quantified by Pierce BCA assay according to manufacturer’s instructions (Thermo Scientific). In all, 2 μg/μL of protein lysate was loaded and samples prepared according to the manufacturer’s instructions with the anti-rabbit detection module (Bio-Techne) and the antibodies listed in Table 5. Analysis was performed with the Wes instrument (Bio-Techne) and Compass for SW software (Bio-Techne). Quantification of pERK was performed in the Compass for SW software, where pERK peaks were normalized to vinculin peaks. Further normalization to the DMSO treated sample for each cell line was performed.

**Table 5:** Simple western antibodies.

Antibody	Manufacturer	Catalog #	Dilution
Ras, mutant G12D specific	Cell Signaling Technology	14429	1:10
Phospho-p44/42 MAPK (Erk1/2) (Thr202/Tyr204)	Cell Signaling Technology	4370	1:10
p44/42 MAPK (Erk1/2)	Cell Signaling Technology	9102	1:10
Vinculin	Abcam	ab129002	1:50

In other experiments, 5 × 10^5^–1 × 10^6^ cells were seeded per well of a six-well plate and 24 h later, treated with MRTX1133 for 3 h, 6 h, or 24 h. Cells were lysed in urea lysis buffer (8 M urea, 2.5% SDS) with cOmplete mini EDTA-free protease inhibitor cocktail and quantified by Pierce BCA assay according to manufacturer’s instructions. 20 μg of protein was loaded per sample onto Bolt 4–12% Bis-Tris Plus gels (Invitrogen) and transferred using the Trans-Blot Turbo RTA Mini PVDF Transfer Kit (Bio-Rad). Membranes were blocked in 5% BSA in TBS with 0.1% Tween-20 (TBS-T) and incubated in the primary antibodies listed in Table 6 overnight at 4 °C. Membranes were washed with TBS-T, followed by incubation in secondary antibody (Table 6) for 1 h at room temperature. Membranes were washed with TBS-T and developed with West-Q Pico ECL Solution (GenDepot). Following development of each antibody, membranes were stripped with 1.5% glycine (pH 2.2), 0.1% SDS, and 1% Tween-20, blocked, and incubated with primary and secondary antibody as described above. Membranes were first probed for RAS^G12D^, followed by pERK, ERK, and vinculin.

**Table 6:** Western blot antibodies.

Antibody	Manufacturer	Catalog #	Dilution
Phospho-p44/42 MAPK (Erk1/2) (Thr202/Tyr204)	Cell Signaling Technology	4370	1:1000
p44/42 MAPK (Erk1/2)	Cell Signaling Technology	9102	1:1000
Ras, mutant G12D specific	Cell Signaling Technology	14429	1:1000
Vinculin	Abcam	ab129002	1:1000
Anti-rabbit IgG, HRP	Abcam	ab16284	1:1000

For proliferation analysis, 5 × 10^4^ cells/mL were seeded in 50 μL of DMEM with 2.5% FBS, 1% PSA, 1 mM sodium pyruvate (Gibco) in 96-well white bottom plates (Corning). 24 h later, media was removed, and cells treated with varying concentrations of MRTX1133 in DMEM with 2.5% FBS, 1% PSA, 1 mM sodium pyruvate (50 μL total volume, with 8 μg/mL puromycin added for shCdk8 experiments). After 3 days, plates were equilibrated to room temperature for 30 min followed by addition of 50 μL of CellTiter-Glo Reagent according to manufacturer’s instructions (Promega). Luminescent signal was measured with a FLUOstar Omega plate reader (BMG LABTECH), the signal in media only wells subtracted, followed by normalization to wells treated with DMSO.

### Digital droplet PCR and whole exome sequencing

DNA was isolated from cells or snap frozen pancreas/tumor tissue using the DNeasy Blood and Tissue Kit (Qiagen) and quantified with the Qubit dsDNA HS Assay Kit (Invitrogen) according to the manufacturer’s instructions. 10 ng of genomic DNA (gDNA) was mixed with 1x QuantStudio 3D Digital PCR Master Mix v2 and 1× *KRAS*^*G12D*^ TaqMan dPCR Liquid Biopsy Assay (Applied Biosystems A44177, Hs000000051), loaded onto QuantStudio 3D Digital PCR 20 K chips (Applied Biosystems), and analyzed with the QuantStudio 3D Digital Real-Time PCR System (Applied Biosystems) and QuantStudio 3D Analysis Suite software (Applied Biosystems). A no template control was used to establish thresholds for the assay.

Mouse Exome panel (Twist Biosciences) was used for whole-exome sequencing according to manufacturer’s instructions. Samples were sequenced with the NovaSeq6000. For data analysis, quality control was conducted for the FASTQ files using FastQC (version 0.11.9, https://www.bioinformatics.babraham.ac.uk/projects/fastqc/) and adapter sequences were removed by Cutadapt (version 4.1) (Martin, 2011). The processed FASTQ files were then mapped to the reference genome (mm10) using bwa mem (version 0.7.17, arXiv:1303.3997v2). The PCR duplicate reads were marked by Picard (version 2.27.4, https://broadinstitute.github.io/picard) and base recalibration was performed using GATK Base Quality Score Recalibration (BQSR) (GATK 4.1.7.0) (Van der Auwera and O’Connor, 2020). Copy number variation analysis was performed by CNVkit (version 0.9.9) (Talevich et al, 2016) for each tumor and cell line sample using the matched tail gDNA sample and parental cell line as reference. The plots related to CNV analysis were generated by the R package ggplot2 (version 3.5.0).

### Quantification and statistical analysis

Graphical representation of the data and statistical tests were performed using GraphPad Prism 10 and reported in the respective figure legends. Sample size was not predetermined for experiments and no blinding was performed. Shapiro–Wilk test was used to assess the normality of distribution of samples. Two-sided unpaired *t* test (for samples with normal distribution and equal variances), unpaired *t* test with Welch’s correction (for normally distributed samples with unequal variances), or two-sided Mann–Whitney test (for samples with non-normal distribution) were performed for comparison of two samples. For comparison of more than two groups, one-way analysis of variance (ANOVA) with Tukey’s or Dunnett’s multiple comparisons test (for samples with normal distribution), Brown–Forsythe and Welch ANOVA with Dunnett’s T3 multiple comparisons test (for samples with unequal variances) and Kruskal–Wallis with Dunn’s multiple comparisons test (for samples with non-normal distribution) were used. Two-way ANOVA with Sidak’s multiple comparisons test was used. Log-rank test or Gehan–Breslow–Wilcoxon test were used to compare Kaplan–Meier survival curves. *P* values are reported as *P < 0.05, **P < 0.01, ***P < 0.001, ****P < 0.0001, *ns*: not significant. Exact *P* values are reported where indicated.

## Supplementary information

Dataset EV1

Peer Review File

Appendix

Figure EV3 Source Data

Appendix Figure S4 Source Data

Expanded View Figures

## Data Availability

scRNA-seq data are deposited at GEO (GSE269679) and spatial transcriptomics data are deposited at GEO (GSE269680). WES data was deposited at SRA (BioProject PRJNA1124144). Source data is available at BioStudies (https://www.ebi.ac.uk/biostudies/, accession number S-BIAD3647). Resources and reagents used here will be available upon reasonable request from RK with a material transfer agreement (MTA) when relevant. The source data of this paper are collected in the following database record: biostudies:S-SCDT-10_1038-S44318-026-00854-5.
