# Supplementary material for: CDK8 remodels the tumor microenvironment and promotes resistance to KRASG12D inhibitors and daraxonrasib in PDAC
Source: EMBO J. 2026 Jul 11;45(16):5727–58. doi: 10.1038/s44318-026-00854-5 (PMC13483915; doi:10.1038/s44318-026-00854-5)
Supplement: Supplementary file 1 [file 44318_2026_854_MOESM2_ESM.pdf]

THE EMBO JOURNAL

CDK8 remodels the tumor microenvironment and promotes resistance to KRASG12D inhibitors and daraxonrasib in PDAC

Kathleen McAndrews, Krishnan Mahadevan, Bingrui Li, Anais Comptdaer, Amari Sockwell, Sami Morse, Patience Kelly, Sarah Patel, Michelle Kirtley, Barbara Moreno Diaz, Hengyu Lyu, David Peng, Sima Khazaei, Aislyn Schalck, Xunian Zhou, Hikaru Sugimoto, Aaron Bickert, Lakshmi Kavitha Sthanam, Meagan Taylor, Shreyasee Kumbhar, Kent Arian, Yasaman Barekatain, Francesca Paradiso, Paola Guerrero, Vincent Bernard, Navid Sobhani, Alondra Camacho-Acevedo, Kiara Bornes, Phuong Thao Tran, Anirban Maitra, Timothy Heffernan, and Raghu Kalluri

Corresponding author: Raghu Kalluri (rkalluri@mdanderson.org)

Review Timeline:

Submission Date:	30th Mar 26
Editorial Decision:	4th May 26
Editor's Correspondence:	8th May 26
Revision Received:	23rd Jun 26
Accepted:	26th Jun 26

Editor: Daniel Klimmeck

Transaction Report:

Please note that the manuscript was previously reviewed at another journal. As EMBO Press has a transfer agreement (including the identities of the referees) with that journal, revision was invited based on the reports from that previous external review.

(Note: With the exception of the correction of typographical or spelling errors that could be a source of ambiguity, letters and reports are not edited. Depending on transfer agreements, referee reports obtained elsewhere may or may not be included in this compilation. Referee reports are anonymous unless the Referee chooses to sign their reports.)

Reviewer #3 (Remarks to the Author):

Summary

The additional work done using RAS inhibitors currently being clinically tested in additional PDX models is appreciated. The inability to analyze human samples due to the companies not allowing reporting is understandable. Addition of this limitation in the discussion, as well as modifying the title, is noted. However, questions remain.

Answer: Thank you for the acknowledging our revisions and that as of today, samples from patients with PDAC are not available from any clinical trials for analyses by independent research groups like ours. Therefore, PDXs are only option for any human correlates analyses, as done here. In this regard, the included analyses using RMC6236, which is currently the most advanced inhibitor in clinical testing with impressive response rates for a single agent targeted therapy, and RMC9805, a $KRAS^{G12D}$(ON) inhibitor currently in phase 3 trials. The reviewer commented that MRTX1133 is not relevant in human PDX experiments because clinical testing has been discontinued to due formulation and bioavailability issues; therefore, we removed that data in the previous revised version.

Major Points

1. Regarding expression of CDK8 levels in Reviewer Fig 1A, it would be striking to see if tumors that were more resistant to RAS inhibitor therapy all had higher levels at baseline or if baseline level is not predictive of response. This comparison would potentially strengthen translatability and the central premise of the manuscript. Across several PDXs, baseline CDK8 levels are higher in sensitive tumors compared to resistant ones. This directly contradicts with the proposed model in which CDK8 promotes resistance and is not adequately addressed. The data more appropriately position CDK8 as a correlatively enriched marker in some resistant contexts, not a validated driver.

Answer: We analyzed baseline CDK8 levels and the correlation is similar to the data at endpoint that is included in the manuscript (**Reviewer Fig. 1**). The reviewer raises the question regarding whether CDK8 is an inherent driver of resistance vs. an acquired driver of resistance, which was addressed in the previous version. While higher baseline CDK8 expression correlating with response may be indicative of its role as an inherent resistance driver, it is more suggestive of clonal selection of $CDK8^{+}$ cells when $KRAS^{G12D}$ targeting resistance is encountered resulting in

Figure for reviewers removed

overall increased CDK8 expression and functional dependency only in response to KRAS inhibitor treatment and ensuing resistance. Importantly, if that was not the case, then treatment of original PDAC tumors in mouse models with CDK8 inhibitors should have led to efficacy when oncogenic $KRAS^{G12D}$ mediated pathways are active and operational (**Reviewer Figure 2**). In addition, the role of CDK8 is not just relevant to cancer cell autonomous functions but also impacts the TME to induce therapy resistance. Treatment with a CDK8 inhibitor overcomes the negative impact on immune microenvironment and enables the efficacy of immunotherapy in $KRAS^{G12D}$ inhibitor resistant tumors (**Figure 5** in the revised manuscript).

Figure for reviewers removed

2. With respect to murine CDK8 levels in the orthotopic experiment (Reviewer Figs 1E-F), the authors need to include expression of CDK8 levels in the vehicle group, especially since they list vehicle pERK expression (Reviewer Fig 1D). Without this, it is not possible to discern is the increase in CDK8 a return to baseline or the increase is only in response to continued pressure from pan-RAS inhibition. The authors have not shown that CDK8 decreases early and increases with the emergence of resistance, as one of the reviewers explicitly recommended. The added timepoints appear selected but not temporally linked to the development of resistance.

Answer: We thank the reviewer for raising this point. We have now included analysis of vehicle treated mice and show that the abundance of CDK8^{+} cells is similar between vehicle and RMC6236 sensitive tumors which increases in RMC6236 resistant tumors (**Reviewer Fig. 3, Supplemental Fig. 5E-G**).

Figure for reviewers removed

3. Regarding PDX data, making comparisons regarding CDK8+ cells between samples (Reviewer Fig 1J) needs to be complimented by making comparisons between each of these and the respective vehicle group as done in Reviewer Fig 1H. This is necessary due to the discrepancy where PDXs PATX124 and PATX155 had similar levels of CDK8 expression after RAS inhibitor treatment (Reviewer Fig 1A) but then different responses to RAS inhibitor therapy (PATX124-Resistance and PATX155-Partial Response, Figs 1G-J). This potentially suggests differing levels at baseline, which could be determinative of response.

Answer: We revised the manuscript to include comparisons to vehicle and observe that CDK8 is not significantly increased in PATX155 following RMC9805 treatment (**Reviewer Fig. 4, Supplemental Fig. 5J-K**). This suggests that baseline levels of CDK8 may be indicative of responses; however, as detailed in our response to major point 1, CDK8 dependency is only encountered resistance to KRAS^{G12D} targeting occurs.

Figure for reviewers removed

4. The additional PDX data remain correlative and do not demonstrate that CDK8 is mechanistically required for resistance to KRAS inhibitors. No CDK8 perturbation studies (inhibition, knockdown, overexpression) were performed and no functional rescue experiments are provided. So how could they claim that CDK8 drives resistance if they didn't knock it down?

Answer: Knockdown and other perturbation studies in patient-derived models are difficult to pursue as these are tumor transplant models and not cell line-derived models. Our data suggests that CDK8 acts cancer cell-extrinsic driver in resistant tumors to remodel the immune microenvironment. PDXs require an immunodeficient background lacking T cells to grow in mice and such studies are not possible.

5. They merged data from MRTX1133, RMC6236, and RMC9805 without addressing major mechanistic differences between these agents. Resistance pathways differ widely across these inhibitors, yet the authors treat CDK8 as a unified node without justification.

Answer: We agree that MRTX1133, RMC6236, and RMC9805 have different mechanisms of action, but all act to suppress $KRAS^{G12D}$ signaling in this context. Despite such differences, CDK8 is a conserved mechanism of resistance across distinct modalities of RAS inhibition, which we view as a strength of our findings and provides rationale for future studies evaluating whether CDK8 upregulation is a conserved immune remodeling driven mechanism of resistance with alternate KRAS* targeting strategies. We revised the discussion to address this point.

6. Human relevance is still limited. Despite added PDX data, no human primary tissue, clinical biopsy, or large-scale transcriptomic correlation is included. Public datasets (TCGA, CPTAC) are not leveraged. The claim of improved human relevance remains overstated.

Answer: We appreciate that clinical biopsy data or large-scale transcriptomics would strengthen our hypotheses; however, TCGA, CPTAC, and other large-scale transcriptomics datasets are not relevant for our studies as they do not include patients treated with RAS inhibitors. Currently, there are no publicly available datasets from $KRAS^{G12D}$ PDAC patients that were treated with RAS inhibitors and the companies sponsoring current RAS inhibitor trials have not approved the publication of any patient data or correlative biopsy data (See answer to comment # 1). As a

result, we included data from cell lines and PDXs and have tempered conclusions related to human relevance in the revised manuscript.

Minor Points

1. Tumors resistant to RMC-6236 had sustained suppression of pERK expression (Reviewer Figs 1C-D). This is interesting since bypass signaling and return of pERK occurs in response to sustained RAS inhibition, as shown by the authors in Main Fig 1F where resistant tumors treated with MRTX1133 had pERK expression return to baseline in the same orthotopic model system (Main Fig 1F). Including tumor volumes/mass at time of tumor excision for all samples would be appreciated.

Answer: We believe that the lack of pERK rebound in RMC6236 treated tumors vs. MRTX1133 treated tumors is due to differences in the mechanism of action of each drug. RMC6236 binds to the active RAS-GTP complex, meaning that it can act to suppress both amplified wildtype RAS signaling and mutant RAS signaling. In contrast, MRTX1133 non-covalently binds to $KRAS^{G12D}$ with high specificity, which still allows for wildtype KRAS signaling which could drive pERK rebound. We revised the manuscript to address differences in the mechanisms of action of the drugs in response to this point and major point 5.

2. What are the treatment durations for each of the PDXs? Is the classification of PD, PR, SD made at the same time point post initiation of treatment or is this the best response observed?

Answer: We apologize for the insufficient experimental details provided for PDX experiments. The treatment duration for the PDXs varied depending on the growth kinetics of each PDX, and the classification reported is the best response observed. We updated the revised manuscript to include this information.

3. Uninterpretable supplementary data. Gene-level expression tables (Cdk8, Nras, Kras, Myc, Yap1, Abcb1a, etc.) show inconsistent changes and are not integrated into any mechanistic interpretation. Their relevance is unclear.

Answer: We appreciate the opportunity to clarify the supplementary data table. Supplementary Table 1 includes a list of copy number variations (CNVs) and their location in the genome identified in tumors and cell lines as opposed to gene expression. The data is a more detailed explanation of the plots included in Supplemental Fig. 5A and 7G-H. We analyzed 3 individual tumors per treatment group, and inconsistent changes reflect heterogeneity in the amplifications/deletions that are present. We revised the text to clarify these points.

Dear Raghu,

Thank you again for the submission of your amended manuscript (EMBOJ-2026-124254) to The EMBO Journal, as well as for your patience with our feedback from the arbitration process. We have carefully assessed your manuscript, and the point-by-point response provided to the referee concerns that were raised during peer-review at a different journal. In addition, and as mentioned before, we decided to send the revised version of your work back to seek original reviewer input with respect to technical robustness, conceptual advance and overall suitability of your work for publication in The EMBO Journal. We have now received the re-report by referee #2 which I enclose below. As you will see from the expert's comments, s/he is now in favour of the work and supportive of publication at The EMBO Journal, pending satisfactory minor revision.

We are thus pleased to inform you that we can offer to swiftly move forward towards acceptance of this work at The EMBO Journal, pending minor revision of the following remaining issues, which need to be adjusted in a re-submitted version.

Please consider the remaining points by the referee #2 carefully and adjust the data presentation and discussion of the results, where appropriate.

We also need you to take care of a number of minor issues related to formatting and data annotation, which I will share shortly in a separate message, together with additional changes and requests by our production team for Source Data provision.

Please submit a revised version of the manuscript using the link enclosed below, addressing the advisor's comments.

As you might remember from previous experience, every paper at the EMBO Journal now includes a 'Synopsis', displayed on the html and freely accessible to all readers. The synopsis includes a 'model' figure as well as 2-5 one-short-sentence bullet points that summarize the article. I would appreciate if you could provide this figure and the bullet points.

Please let me know any time should you have additional questions regarding above points.

Thank you again for giving us the chance to consider your manuscript for The EMBO Journal, I look forward to hearing from you and receiving your final revised version of the manuscript.

Best regards,

Daniel

Daniel Klimmeck PhD
Senior Editor
The EMBO Journal

Please remember: Digital image enhancement is acceptable practice, as long as it accurately represents the original data and conforms to community standards. If a figure has been subjected to significant electronic manipulation, this must be noted in the figure legend or in the 'Methods' section. The editors reserve the right to request original versions of figures and the original images that were used to assemble the figure.

Referee #2, arbitrating re-comments:

I served as a reviewer for this manuscript submitted to another venue previously.

In summary, I valued the scientific significance and translational potential of the study, but less enthusiasm on the causal role of CDK8 levels on the development of drug resistance, and its direct human relevance (difficulty in testing human PDAC samples).

For this review, I have made comments one-by-one on the authors' rebuttal letter as detailed below. I support the publication of this paper in EMBO, if the authors would address my minor critiques, and are willing to tune down the strong statement on causal role of CDK8 in drug resistance and human relevance.

Referee #2, arbitrating re-comments on Author rebuttal response:

Reviewer #3 (Remarks to the Author):

Summary: The additional work done using RAS inhibitors currently being clinically tested in additional PDX models is appreciated. The inability to analyze human samples due to the companies not allowing reporting is understandable. Addition of this limitation in the discussion, as well as modifying the title, is noted. However, questions remain.

Answer: Thank you for the acknowledging our revisions and that as of today, samples from patients with PDAC are not available from any clinical trials for analyses by independent research groups like ours. Therefore, PDXs are only option for any human correlates analyses, as done here. In this regard, the included analyses using RMC6236, which is currently the most advanced inhibitor in clinical testing with impressive response rates for a single agent targeted therapy, and RMC9805, a KRASG12D(ON) inhibitor currently in phase 3 trials. The reviewer commented that MRTX1133 is not relevant in human PDX experiments because clinical testing has been discontinued to due formulation and bioavailability issues; therefore, we removed that data in the previous revised version.

@Arbitrating comment Ref#2: Justified and agreed.

Major Points

1. Regarding expression of CDK8 levels in Reviewer Fig 1A, it would be striking to see if tumors that were more resistant to RAS inhibitor therapy all had higher levels at baseline or if baseline level is not predictive of response. This comparison would potentially strengthen translatability and the central premise of the manuscript. Across several PDXs, baseline CDK8 levels are higher in sensitive tumors compared to resistant ones. This directly contradicts with the proposed model in which CDK8 promotes resistance and is not adequately addressed. The data more appropriately position CDK8 as a correlatively enriched marker in some resistant contexts, not a validated driver.

Answer: We analyzed baseline CDK8 levels and the correlation is similar to the data at endpoint that is included in the manuscript (Reviewer Fig. 1). The reviewer raises the question regarding whether CDK8 is an inherent driver of resistance vs. an acquired driver of resistance, which was addressed in the previous version. While higher baseline CDK8 expression correlating with response may be indicative of its role as an inherent resistance driver, it is more suggestive of clonal selection of CDK8+ cells when KRASG12D targeting resistance is encountered resulting in overall increased CDK8 expression and functional dependency only in response to KRAS inhibitor treatment and ensuing resistance. Importantly, if that was not the case, then treatment of original PDAC tumors in mouse models with CDK8 inhibitors should have led to efficacy when oncogenic KRASG12D mediated pathways are active and operational (Reviewer Figure 2). In addition, the role of CDK8 is not just relevant to cancer cell autonomous functions but also impacts the TME to induce therapy resistance. Treatment with a CDK8 inhibitor overcomes the negative impact on immune microenvironment and enables the efficacy of immunotherapy in KRASG12D inhibitor resistant tumors (Figure 5 in the revised manuscript).

@Arbitrating comment Ref#2: The results largely support the conclusion. The point on "whether CDK8 is an inherent driver of resistance vs. an acquired driver of resistance" should be discussed in the Discussion section, as argued here.

2. With respect to murine CDK8 levels in the orthotopic experiment (Reviewer Figs 1E-F), the authors need to include expression of CDK8 levels in the vehicle group, especially since they list vehicle pERK expression (Reviewer Fig 1D). Without this, it is not possible to discern is the increase in CDK8 a return to baseline or the increase is only in response to continued pressure from pan-RAS inhibition. The authors have not shown that CDK8 decreases early and increases with the emergence of resistance, as one of the reviewers explicitly recommended. The added timepoints appear selected but not temporally linked to the development of resistance.

Answer: We thank the reviewer for raising this point. We have now included analysis of vehicle treated mice and show that the abundance of CDK8+ cells is similar between vehicle and RMC6236 sensitive tumors which increases in RMC6236 resistant tumors (Reviewer Fig. 3, Supplemental Fig. 5E-G).

@Arbitrating comment Ref#2: The point was fully addressed.

3. Regarding PDX data, making comparisons regarding CDK8+ cells between samples (Reviewer Fig 1J) needs to be complimented by making comparisons between each of these and the respective vehicle group as done in Reviewer Fig 1H. This is necessary due to the discrepancy where PDXs PATX124 and PATX155 had similar levels of CDK8 expression after RAS inhibitor treatment (Reviewer Fig 1A) but then different responses to RAS inhibitor therapy (PATX124-Resistance and PATX155-Partial Response, Figs 1G-J). This potentially suggests differing levels at baseline, which could be determinative of response.

Answer: We revised the manuscript to include comparisons to vehicle and observe that CDK8 is not significantly increased in PATX155 following RMC9805 treatment (Reviewer Fig. 4, Supplemental Fig. 5J-K). This suggests that baseline levels of CDK8 may be indicative of responses; however, as detailed in our response to major point 1, CDK8 dependency is only encountered resistance to KRASG12D targeting occurs.

@Arbitrating comment Ref#2: The argument is not well supported by the data. The authors claimed that "CDK8 is not significantly increased in PATX155 following RMC9805 treatment". However, the data showed two-fold increase on average in treatment group, as compared to vehicle control. The lack of statistically significant difference is largely due to sample variations. What is the sample size in each group?

4. The additional PDX data remain correlative and do not demonstrate that CDK8 is mechanistically required for resistance to KRAS inhibitors. No CDK8 perturbation studies (inhibition, knockdown, overexpression) were performed and no functional rescue experiments are provided. So how could they claim that CDK8 drives resistance if they didn't knock it down?

Answer: Knockdown and other perturbation studies in patient-derived models are difficult to pursue as these are tumor transplant models and not cell line-derived models. Our data suggests that CDK8 acts cancer cell-extrinsic driver in resistant tumors to remodel the immune microenvironment. PDXs require an immunodeficient background lacking T cells to grow in mice and such studies are not possible.

@Arbitrating comment Ref#2: I agree with both reviewer and the authors. If this critical experiment cannot be done, the authors should tone down their strong conclusion.

5. They merged data from MRTX1133, RMC6236, and RMC9805 without addressing major mechanistic differences between these agents. Resistance pathways differ widely across these inhibitors, yet the authors treat CDK8 as a unified node without justification.

Answer: We agree that MRTX1133, RMC6236, and RMC9805 have different mechanisms of action, but all act to suppress KRASG12D signaling in this context. Despite such differences, CDK8 is a conserved mechanism of resistance across distinct modalities of RAS inhibition, which we view as a strength of our findings and provides rationale for future studies evaluating whether CDK8 upregulation is a conserved immune remodeling driven mechanism of resistance with alternate KRAS* targeting strategies. We revised the discussion to address this point.

@Arbitrating comment Ref#2: I largely agree. This important issue needs to be raised and thoroughly discussed.

6. Human relevance is still limited. Despite added PDX data, no human primary tissue, clinical biopsy, or large-scale transcriptomic correlation is included. Public datasets (TCGA, CPTAC) are not leveraged. The claim of improved human relevance remains overstated.

Answer: We appreciate that clinical biopsy data or large-scale transcriptomics would strengthen our hypotheses; however, TCGA, CPTAC, and other large-scale transcriptomics datasets are not relevant for our studies as they do not include patients treated with RAS inhibitors. Currently, there are no publicly available datasets from KRASG12D PDAC patients that were treated with RAS inhibitors and the companies sponsoring current RAS inhibitor trials have not approved the publication of any patient data or correlative biopsy data (See answer to comment # 1). As a result, we included data from cell lines and PDXs and have tempered conclusions related to human relevance in the revised manuscript.

@Arbitrating comment Ref#2: Again, I agree with both the reviewer and the authors. Thus, the strong conclusion on human relevance should be tune down, given lack of strong supporting data.

Minor Points

1. Tumors resistant to RMC-6236 had sustained suppression of pERK expression (Reviewer Figs 1C-D). This is interesting since bypass signaling and return of pERK occurs in response to sustained RAS inhibition, as shown by the authors in Main Fig 1F where resistant tumors treated with MRTX1133 had pERK expression return to baseline in the same orthotopic model system (Main Fig 1F). Including tumor volumes/mass at time of tumor excision for all samples would be appreciated.

Answer: We believe that the lack of pERK rebound in RMC6236 treated tumors vs. MRTX1133 treated tumors is due to differences in the mechanism of action of each drug. RMC6236 binds to the active RAS-GTP complex, meaning that it can act to suppress both amplified wildtype RAS signaling and mutant RAS signaling. In contrast, MRTX1133 non-covalently binds to KRASG12D with high specificity, which still allows for wildtype KRAS signaling which could drive pERK rebound. We revised the manuscript to address differences in the mechanisms of action of the drugs in response to this point and major point 5.

@Arbitrating comment Ref#2: Agreed.

2. What are the treatment durations for each of the PDXs? Is the classification of PD, PR, SD made at the same time point post initiation of treatment or is this the best response observed?

Answer: We apologize for the insufficient experimental details provided for PDX experiments. The treatment duration for the PDXs varied depending on the growth kinetics of each PDX, and the classification reported is the best response observed. We updated the revised manuscript to include this information.

@Arbitrating comment Ref#2: Agreed.

3. Uninterpretable supplementary data. Gene-level expression tables (Cdk8, Nras, Kras, Myc, Yap1, Abcb1a, etc.) show inconsistent changes and are not integrated into any mechanistic interpretation. Their relevance is unclear.

Answer: We appreciate the opportunity to clarify the supplementary data table. Supplementary Table 1 includes a list of copy number variations (CNVs) and their location in the genome identified in tumors and cell lines as opposed to gene expression. The data is a more detailed explanation of the plots included in Supplemental Fig. 5A and 7G-H. We analyzed 3 individual tumors per treatment group, and inconsistent changes reflect heterogeneity in the amplifications/deletions that are present. We revised the text to clarify these points.

@Arbitrating comment Ref#2: OK

Dear Raghu,

Further to below, I attach the mentioned list of formatting changes required for your final resubmission.

Please let us know any time should you have additional questions related.

Best regards,

Daniel

Daniel Klimmeck PhD
Senior Editor
The EMBO Journal

******

Formatting changes required for the revised version of the manuscript:

>> Please provide the main manuscript text as .docx file.

>> Figures in separate files: Figures should be removed from the manuscript text and uploaded as individual, high resolution figure files. Legends should be placed after the References.

>> Author Contributions: Remove the author contributions information from the manuscript text. Note that CRediT has replaced the traditional author contributions section as of now because it offers a systematic machine-readable author contributions format that allows for more effective research assessment. and use the free text boxes beneath each contributing author's name to add specific details on the author's contribution.

More information is available in our guide to authors.
https://www.embopress.org/page/journal/14602075/authorguide

>> Provide a completed Author Checklist.

>> Adjust the title of the 'Declaration of Interests' section to 'Disclosure and Competing Interests Statement'.

>> Section order should be corrected as follows: title page with complete author information, abstract, keywords, introduction, results, discussion, methods, data availability section, acknowledgements, disclosure and competing interests statement, references, main figure legends, tables, expanded view figure legends.´.

>> Figure callouts: Please ensure that the figures and panels are called out in sequential order. In addition, currently, a callout for Supplemental Table 1 needs to be added.

>> References: please adjust reference format to EMBO Journal format, 10 authors et al.

>> Please recheck references for the bioRxiv entries Mahadevan et al 2025a, 2025b and update the citations if in the meantime published as regular article.

>> Please add a Reagents and Tools table to the Methods section, as a separate file using the existing template in the Guide

For Authors, listing key reagents, experimental models, software and relevant equipment.

Please compare also our Guide-to-Authors instructions:
https://www.embopress.org/page/journal/14602075/authorguide#structuredmethods

>> Funding: add the 'Sid W. Richardson Foundation' to the list of funders indicated in our online system.

>>Appendix file with ToC: Appendix file needs to be in PDF format; title page with "Appendix for + manuscript title" (author and affiliation list not necessary) and ToC with page numbers of listed items missing; nomenclature should be Appendix Figure Sx throughout the manuscript and Appendix PDF

>> Dataset EV legends: source file name, title, legend and manuscript callout all need to be updated to Dataset EV1 instead of Table S1, legend should be removed from Appendix ("Supplementary Information") and uploaded as a separate tab/sheet in the Excel file.

>> Please provide source data for the study as to the separate request e-mail. Source data should be uploaded as one (zipped) file per figure.

>> Data availability section: specify all databases and datsets used adding hyperlinks.
Remove referee tokens and make sure datasets are made publicly accessible.

>> Consider additional changes and comments from our production team as indicated below:

DATA CHECK: PASS: Please note that the specific URLs for GSE269679, GSE269680, PRJNA1124144 datasets are not provided in the data availability statement.

- Data citations: no comments
- Figure Legends (main + EV):
1. Please note that the exact p values are not provided in the legends of figures 1B, D, F, I, M, Q; 2H, J, L, Q; 3B, E, G, J, K, L, M, O; 4B, C, E, F, G, H, J, L; 5C, E, G, H, K, L
2. Please note that the box plot needs to be defined in terms of minima, maxima, centre, bounds of box and whiskers, and percentile in the legends of figure 3G

Further information is available in our Guide For Authors: https://link.springer.com/journal/44318/submission-guidelines

Please use the link below to submit your revision:

https://emboj.msubmit.net/cgi-bin/main.plex

Dear Raghu,

Thank you again for the submission of your amended manuscript (EMBOJ-2026-124254) to The EMBO Journal, as well as for your patience with our feedback from the arbitration process. We have carefully assessed your manuscript, and the point-by-point response provided to the referee concerns that were raised during peer-review at a different journal. In addition, and as mentioned before, we decided to send the revised version of your work back to seek original reviewer input with respect to technical robustness, conceptual advance and overall suitability of your work for publication in The EMBO Journal. We have now received the re-report by referee #2 which I enclose below. As you will see from the expert's comments, s/he is now in favour of the work and supportive of publication at The EMBO Journal, pending satisfactory minor revision.

We are thus pleased to inform you that we can offer to swiftly move forward towards acceptance of this work at The EMBO Journal, pending minor revision of the following remaining issues, which need to be adjusted in a re-submitted version.

Please consider the remaining points by the referee #2 carefully and adjust the data presentation and discussion of the results, where appropriate.

We also need you to take care of a number of minor issues related to formatting and data annotation, which I will share shortly in a separate message, together with additional changes and requests by our production team for Source Data provision.

Please submit a revised version of the manuscript using the link enclosed below, addressing the advisor's comments.

As you might remember from previous experience, every paper at the EMBO Journal now includes a 'Synopsis', displayed on the html and freely accessible to all readers. The synopsis includes a 'model' figure as well as 2-5 one-short-sentence bullet points that summarize the article. I would appreciate if you could provide this figure and the bullet points.

Please let me know any time should you have additional questions regarding above points.

Thank you again for giving us the chance to consider your manuscript for The EMBO Journal, I look forward to hearing from you and receiving your final revised version of the manuscript.

Best regards,

Daniel

Daniel Klimmeck PhD
Senior Editor
The EMBO Journal

Please remember: Digital image enhancement is acceptable practice, as long as it accurately represents the original data and conforms to community standards. If a figure has been subjected to significant electronic manipulation, this must be noted in the figure legend or in the 'Methods' section. The editors reserve the right to request original versions of figures and the original images that were used to assemble the figure.

Referee #2, arbitrating re-comments:

I served as a reviewer for this manuscript submitted to another venue previously.

In summary, I valued the scientific significance and translational potential of the study, but less enthusiasm on the causal role of CDK8 levels on the development of drug resistance, and its direct human relevance (difficulty in testing human PDAC samples).

For this review, I have made comments one-by-one on the authors' rebuttal letter as detailed below. I support the publication of this paper in EMBO, if the authors would address my minor critiques, and are willing to tune down the strong statement on causal role of CDK8 in drug resistance and human relevance.

Referee #2, arbitrating re-comments on Author rebuttal response:

Reviewer #3 (Remarks to the Author):

Summary: The additional work done using RAS inhibitors currently being clinically tested in additional PDX models is appreciated. The inability to analyze human samples due to the companies not allowing reporting is understandable. Addition of this limitation in the discussion, as well as modifying the title, is noted. However, questions remain.

Answer: Thank you for the acknowledging our revisions and that as of today, samples from patients with PDAC are not available from any clinical trials for analyses by independent research groups like ours. Therefore, PDXs are only option for any human correlates analyses, as done here. In this regard, the included analyses using RMC6236, which is currently the most advanced inhibitor in clinical testing with impressive response rates for a single agent targeted therapy, and RMC9805, a KRASG12D(ON) inhibitor currently in phase 3 trials. The reviewer commented that MRTX1133 is not relevant in human PDX experiments because clinical testing has been discontinued to due formulation and bioavailability issues; therefore, we removed that data in the previous revised version.

@Arbitrating comment Ref#2: Justified and agreed.

Major Points

1. Regarding expression of CDK8 levels in Reviewer Fig 1A, it would be striking to see if tumors that were more resistant to RAS inhibitor therapy all had higher levels at baseline or if baseline level is not predictive of response. This comparison would potentially strengthen translatability and the central premise of the manuscript. Across several PDXs, baseline CDK8 levels are higher in sensitive tumors compared to resistant ones. This directly contradicts with the proposed model in which CDK8 promotes resistance and is not adequately addressed. The data more appropriately position CDK8 as a correlatively enriched marker in some resistant contexts, not a validated driver.

Answer: We analyzed baseline CDK8 levels and the correlation is similar to the data at endpoint that is included in the manuscript (Reviewer Fig. 1). The reviewer raises the question regarding whether CDK8 is an inherent driver of resistance vs. an acquired driver of resistance, which was addressed in the previous version. While higher baseline CDK8 expression correlating with response may be indicative of its role as an inherent resistance driver, it is more suggestive of clonal selection of CDK8+ cells when KRASG12D targeting resistance is encountered resulting in overall increased CDK8 expression and functional dependency only in response to KRAS inhibitor treatment and ensuing resistance. Importantly, if that was not the case, then treatment of original PDAC tumors in mouse models with CDK8 inhibitors should have led to efficacy when oncogenic KRASG12D mediated pathways are active and operational (Reviewer Figure 2). In addition, the role of CDK8 is not just relevant to cancer cell autonomous functions but also impacts the TME to induce therapy resistance. Treatment with a CDK8 inhibitor overcomes the negative impact on immune microenvironment and enables the efficacy of immunotherapy in KRASG12D inhibitor resistant tumors (Figure 5 in the revised manuscript).

@Arbitrating comment Ref#2: The results largely support the conclusion. The point on "whether CDK8 is an inherent driver of resistance vs. an acquired driver of resistance" should be discussed in the Discussion section, as argued here.

2. With respect to murine CDK8 levels in the orthotopic experiment (Reviewer Figs 1E-F), the authors need to include expression of CDK8 levels in the vehicle group, especially since they list vehicle pERK expression (Reviewer Fig 1D). Without this, it is not possible to discern is the increase in CDK8 a return to baseline or the increase is only in response to continued pressure from pan-RAS inhibition. The authors have not shown that CDK8 decreases early and increases with the emergence of resistance, as one of the reviewers explicitly recommended. The added timepoints appear selected but not temporally linked to the development of resistance.

Answer: We thank the reviewer for raising this point. We have now included analysis of vehicle treated mice and show that the abundance of CDK8+ cells is similar between vehicle and RMC6236 sensitive tumors which increases in RMC6236 resistant tumors (Reviewer Fig. 3, Supplemental Fig. 5E-G).

@Arbitrating comment Ref#2: The point was fully addressed.

3. Regarding PDX data, making comparisons regarding CDK8+ cells between samples (Reviewer Fig 1J) needs to be complimented by making comparisons between each of these and the respective vehicle group as done in Reviewer Fig 1H. This is necessary due to the discrepancy where PDXs PATX124 and PATX155 had similar levels of CDK8 expression after RAS inhibitor treatment (Reviewer Fig 1A) but then different responses to RAS inhibitor therapy (PATX124-Resistance and PATX155-Partial Response, Figs 1G-J). This potentially suggests differing levels at baseline, which could be determinative of response.

Answer: We revised the manuscript to include comparisons to vehicle and observe that CDK8 is not significantly increased in PATX155 following RMC9805 treatment (Reviewer Fig. 4, Supplemental Fig. 5J-K). This suggests that baseline levels of CDK8 may be indicative of responses; however, as detailed in our response to major point 1, CDK8 dependency is only encountered resistance to KRASG12D targeting occurs.

@Arbitrating comment Ref#2: The argument is not well supported by the data. The authors claimed that "CDK8 is not significantly increased in PATX155 following RMC9805 treatment". However, the data showed two-fold increase on average in treatment group, as compared to vehicle control. The lack of statistically significant difference is largely due to sample variations. What is the sample size in each group?

4. The additional PDX data remain correlative and do not demonstrate that CDK8 is mechanistically required for resistance to KRAS inhibitors. No CDK8 perturbation studies (inhibition, knockdown, overexpression) were performed and no functional rescue experiments are provided. So how could they claim that CDK8 drives resistance if they didn't knock it down?

Answer: Knockdown and other perturbation studies in patient-derived models are difficult to pursue as these are tumor transplant models and not cell line-derived models. Our data suggests that CDK8 acts cancer cell-extrinsic driver in resistant tumors to remodel the immune microenvironment. PDXs require an immunodeficient background lacking T cells to grow in mice and such studies are not possible.

@Arbitrating comment Ref#2: I agree with both reviewer and the authors. If this critical experiment cannot be done, the authors should tone down their strong conclusion.

5. They merged data from MRTX1133, RMC6236, and RMC9805 without addressing major mechanistic differences between these agents. Resistance pathways differ widely across these inhibitors, yet the authors treat CDK8 as a unified node without justification.

Answer: We agree that MRTX1133, RMC6236, and RMC9805 have different mechanisms of action, but all act to suppress KRASG12D signaling in this context. Despite such differences, CDK8 is a conserved mechanism of resistance across distinct modalities of RAS inhibition, which we view as a strength of our findings and provides rationale for future studies evaluating whether CDK8 upregulation is a conserved immune remodeling driven mechanism of resistance with alternate KRAS* targeting strategies. We revised the discussion to address this point.

@Arbitrating comment Ref#2: I largely agree. This important issue needs to be raised and thoroughly discussed.

6. Human relevance is still limited. Despite added PDX data, no human primary tissue, clinical biopsy, or large-scale transcriptomic correlation is included. Public datasets (TCGA, CPTAC) are not leveraged. The claim of improved human relevance remains overstated.

Answer: We appreciate that clinical biopsy data or large-scale transcriptomics would strengthen our hypotheses; however, TCGA, CPTAC, and other large-scale transcriptomics datasets are not relevant for our studies as they do not include patients treated with RAS inhibitors. Currently, there are no publicly available datasets from KRASG12D PDAC patients that were treated with RAS inhibitors and the companies sponsoring current RAS inhibitor trials have not approved the publication of any patient data or correlative biopsy data (See answer to comment # 1). As a result, we included data from cell lines and PDXs and have tempered conclusions related to human relevance in the revised manuscript.

@Arbitrating comment Ref#2: Again, I agree with both the reviewer and the authors. Thus, the strong conclusion on human relevance should be tune down, given lack of strong supporting data.

Minor Points

1. Tumors resistant to RMC-6236 had sustained suppression of pERK expression (Reviewer Figs 1C-D). This is interesting since bypass signaling and return of pERK occurs in response to sustained RAS inhibition, as shown by the authors in Main Fig 1F where resistant tumors treated with MRTX1133 had pERK expression return to baseline in the same orthotopic model system (Main Fig 1F). Including tumor volumes/mass at time of tumor excision for all samples would be appreciated.

Answer: We believe that the lack of pERK rebound in RMC6236 treated tumors vs. MRTX1133 treated tumors is due to differences in the mechanism of action of each drug. RMC6236 binds to the active RAS-GTP complex, meaning that it can act to suppress both amplified wildtype RAS signaling and mutant RAS signaling. In contrast, MRTX1133 non-covalently binds to KRASG12D with high specificity, which still allows for wildtype KRAS signaling which could drive pERK rebound. We revised the manuscript to address differences in the mechanisms of action of the drugs in response to this point and major point 5.

@Arbitrating comment Ref#2: Agreed.

2. What are the treatment durations for each of the PDXs? Is the classification of PD, PR, SD made at the same time point post initiation of treatment or is this the best response observed?

Answer: We apologize for the insufficient experimental details provided for PDX experiments. The treatment duration for the PDXs varied depending on the growth kinetics of each PDX, and the classification reported is the best response observed. We updated the revised manuscript to include this information.

@Arbitrating comment Ref#2: Agreed.

3. Uninterpretable supplementary data. Gene-level expression tables (Cdk8, Nras, Kras, Myc, Yap1, Abcb1a, etc.) show inconsistent changes and are not integrated into any mechanistic interpretation. Their relevance is unclear.

Answer: We appreciate the opportunity to clarify the supplementary data table. Supplementary Table 1 includes a list of copy number variations (CNVs) and their location in the genome identified in tumors and cell lines as opposed to gene expression. The data is a more detailed explanation of the plots included in Supplemental Fig. 5A and 7G-H. We analyzed 3 individual tumors per treatment group, and inconsistent changes reflect heterogeneity in the amplifications/deletions that are present. We revised the text to clarify these points.

@Arbitrating comment Ref#2: OK

Dear Daniel: Thank you for providing us with the reassessment of our revised manuscript. We appreciate the opportunity to see this work published in the EMBO J. Please see our pointwise response below.

Referee #2, arbitrating re-comments:

I served as a reviewer for this manuscript submitted to another venue previously.

In summary, I valued the scientific significance and translational potential of the study, but less enthusiasm on the causal role of CDK8 levels on the development of drug resistance, and its direct human relevance (difficulty in testing human PDAC samples).

For this review, I have made comments one-by-one on the authors' rebuttal letter as detailed below. I support the publication of this paper in EMBO, if the authors would address my minor critiques, and are willing to tune down the strong statement on causal role of CDK8 in drug resistance and human relevance.

Answer: Thank you for the assessment of our work.

Referee #2, arbitrating re-comments on Author rebuttal response:

Reviewer #3 (Remarks to the Author):

Summary: The additional work done using RAS inhibitors currently being clinically tested in additional PDX models is appreciated. The inability to analyze human samples due to the companies not allowing reporting is understandable. Addition of this limitation in the discussion, as well as modifying the title, is noted. However, questions remain.

@Arbitrating comment Ref#2: Justified and agreed.

Major Points
1. Regarding expression of CDK8 levels in Reviewer Fig 1A, it would be striking to see if tumors that were more resistant to RAS inhibitor therapy all had higher levels at baseline or if baseline level is not predictive of response. This comparison would potentially strengthen translatability and the central premise of the manuscript. Across several PDXs, baseline CDK8 levels are higher in sensitive tumors compared to resistant ones. This directly contradicts with the proposed model in which CDK8 promotes resistance and is not adequately addressed. The data more appropriately position CDK8 as a correlatively enriched marker in some resistant contexts, not a validated driver.

@Arbitrating comment Ref#2: The results largely support the conclusion. The point on "whether CDK8 is an inherent driver of resistance vs. an acquired driver of resistance" should be discussed in the Discussion section, as argued here.

Answer: We revised the discussion section to include these points.

2. With respect to murine CDK8 levels in the orthotopic experiment (Reviewer Figs 1E-F), the authors need to include expression of CDK8 levels in the vehicle group, especially since they list

vehicle pERK expression (Reviewer Fig 1D). Without this, it is not possible to discern is the increase in CDK8 a return to baseline or the increase is only in response to continued pressure from pan-RAS inhibition. The authors have not shown that CDK8 decreases early and increases with the emergence of resistance, as one of the reviewers explicitly recommended. The added timepoints appear selected but not temporally linked to the development of resistance.

@Arbitrating comment Ref#2: The point was fully addressed.

3. Regarding PDX data, making comparisons regarding CDK8+ cells between samples (Reviewer Fig 1J) needs to be complimented by making comparisons between each of these and the respective vehicle group as done in Reviewer Fig 1H. This is necessary due to the discrepancy where PDXs PATX124 and PATX155 had similar levels of CDK8 expression after RAS inhibitor treatment (Reviewer Fig 1A) but then different responses to RAS inhibitor therapy (PATX124-Resistance and PATX155-Partial Response, Figs 1G-J). This potentially suggests differing levels at baseline, which could be determinative of response.

@Arbitrating comment Ref#2: The argument is not well supported by the data. The authors claimed that "CDK8 is not significantly increased in PATX155 following RMC9805 treatment". However, the data showed two-fold increase on average in treatment group, as compared to vehicle control. The lack of statistically significant difference is largely due to sample variations. What is the sample size in each group?

Answer: 4 tumors per condition were included in the analysis. We revised the text to reflect that PATX155, which has a PR to RMC9805, shows a trend towards increased CDK8, albeit not statistically significant (P=0.1099).

4. The additional PDX data remain correlative and do not demonstrate that CDK8 is mechanistically required for resistance to KRAS inhibitors. No CDK8 perturbation studies (inhibition, knockdown, overexpression) were performed and no functional rescue experiments are provided. So how could they claim that CDK8 drives resistance if they didn't knock it down?

@Arbitrating comment Ref#2: I agree with both reviewer and the authors. If this critical experiment cannot be done, the authors should tone down their strong conclusion.

Answer: We revised the text to temper conclusions about human relevance.

5. They merged data from MRTX1133, RMC6236, and RMC9805 without addressing major mechanistic differences between these agents. Resistance pathways differ widely across these inhibitors, yet the authors treat CDK8 as a unified node without justification.

Answer: We agree that MRTX1133, RMC6236, and RMC9805 have different mechanisms of action, but all act to suppress KRASG12D signaling in this context because the model is driven by oncogenic KrasG12D. Despite such differences, CDK8 is a conserved mechanism of resistance across distinct modalities of RAS inhibition, which we view as a strength of our findings and provides rationale for future studies evaluating whether CDK8 upregulation is a conserved immune remodeling driven mechanism of resistance with alternate KRAS* targeting strategies. Our conclusions are driven the collected data. We revised the discussion to address this point.

@Arbitrating comment Ref#2: I largely agree. This important issue needs to be raised and thoroughly discussed.

Answer: We revised the discussion to further discuss this point.

6. Human relevance is still limited. Despite added PDX data, no human primary tissue, clinical biopsy, or large-scale transcriptomic correlation is included. Public datasets (TCGA, CPTAC) are not leveraged. The claim of improved human relevance remains overstated.

@Arbitrating comment Ref#2: Again, I agree with both the reviewer and the authors. Thus, the strong conclusion on human relevance should be tune down, given lack of strong supporting data.

Answer: We revised the text to temper conclusions about human relevance.

Minor Points

1. Tumors resistant to RMC-6236 had sustained suppression of pERK expression (Reviewer Figs 1C-D). This is interesting since bypass signaling and return of pERK occurs in response to sustained RAS inhibition, as shown by the authors in Main Fig 1F where resistant tumors treated with MRTX1133 had pERK expression return to baseline in the same orthotopic model system (Main Fig 1F). Including tumor volumes/mass at time of tumor excision for all samples would be appreciated.

@Arbitrating comment Ref#2: Agreed.

2. What are the treatment durations for each of the PDXs? Is the classification of PD, PR, SD made at the same time point post initiation of treatment or is this the best response observed?

Answer: We apologize for the insufficient experimental details provided for PDX experiments. The treatment duration for the PDXs varied depending on the growth kinetics of each PDX, and the classification reported is the best response observed. We updated the revised manuscript to include this information.

@Arbitrating comment Ref#2: Agreed.

3. Uninterpretable supplementary data. Gene-level expression tables (Cdk8, Nras, Kras, Myc, Yap1, Abcb1a, etc.) show inconsistent changes and are not integrated into any mechanistic interpretation. Their relevance is unclear.

@Arbitrating comment Ref#2: OK

Thank you for your patience and the time.

Regards,

Raghu

Dear Raghu,

Thank you for submitting the revised version of your manuscript. I have now evaluated your amended manuscript and concluded that the remaining minor concerns have been sufficiently addressed.

I am thus pleased to inform you that your manuscript has been accepted for publication in the EMBO Journal.

Your manuscript will be processed for publication by EMBO Press. It will be copy edited and you will receive page proofs prior to publication. Please note that you will be contacted by Springer Nature Author Services to complete licensing and payment information.

Please note that it is The EMBO Journal policy for the transcript of the editorial process (containing referee reports and your response letter) to be published as an online supplement to each paper. If you do NOT want this, you will need to inform the Editorial Office via email immediately. More information is available here: https://www.embopress.org/transparent-process#Review_Process

You may qualify for financial assistance for your publication charges - either via a Springer Nature fully open access agreement or an EMBO initiative. Check your eligibility: https://www.embopress.org/page/journal/14602075/authorguide#chargesguide

Should you be planning a Press Release on your article, please notify the production team via this form in order to coordinate publication and release dates: https://forms.cloud.microsoft/e/eH4A8JVP6g

If you have any questions, please do not hesitate to contact the Editorial Office. Thank you for your contribution to The EMBO Journal.

Thank you again for this contribution to The EMBO Journal and congratulations on a successful publication! Please consider us again in the future for your most exciting work.

Best regards,

Daniel

Daniel Klimmeck, PhD
Senior Editor
The EMBO Journal
EMBO
Postfach 1022-40
Meyerhofstrasse 1
D-69117 Heidelberg
contact@embojournal.org

*****
Please note that it is The EMBO Journal policy for the transcript of the editorial process (containing referee reports and your response letters) to be published as an online supplement to each paper. If you should prefer removal of any referee-only figures

included in the point-by-point response(s), e.g. because they may still be used for future publication or because they have been reproduced from published work by others, please do let us know immediately via response email.
More information is available here: https://link.springer.com/partners/embo-press/editorial-policies#Peer%20review

** Click here to be directed to your login page: https://emboj.msubmit.net

EMBO Press Author Checklist

Corresponding Author Name: Raghu Kalluri	
Journal Submitted to: EMBO Journal	
Manuscript Number: EMBOJ-2026-124254	

USEFUL LINKS FOR COMPLETING THIS FORM
The EMBO Journal - Author Guidelines
EMBO Reports - Author Guidelines
Molecular Systems Biology - Author Guidelines
EMBO Molecular Medicine - Author Guidelines

Reporting Checklist for Life Science Articles (updated January
This checklist is adapted from Materials Design Analysis Reporting (MDAR) Checklist for Authors. MDAR establishes a minimum set of requirements in transparent reporting in the life sciences (see Statement of Task: 10.31222/osf.io/9sm4x). Please follow the journal's guidelines in preparing your
Please note that a copy of this checklist will be published alongside your article.

Abridged guidelines for figures
1. Data
The data shown in figures should satisfy the following conditions:
- the data were obtained and processed according to the field's best practice and are presented to reflect the results of the experiments in an accurate and unbiased manner.
- ideally, figure panels should include only measurements that are directly comparable to each other and obtained with the same assay.
- plots include clearly labeled error bars for independent experiments and sample sizes. Unless justified, error bars should not be shown for technical
- if n<5, the individual data points from each experiment should be plotted. Any statistical test employed should be justified.
- Source Data should be included to report the data underlying figures according to the guidelines set out in the authorship guidelines on Data

2. Captions
Each figure caption should contain the following information, for each panel where they are relevant:
- a specification of the experimental system investigated (eg cell line, species name).
- the assay(s) and method(s) used to carry out the reported observations and measurements.
- an explicit mention of the biological and chemical entity(ies) that are being measured.
- an explicit mention of the biological and chemical entity(ies) that are altered/varied/perturbed in a controlled manner.
- the exact sample size (n) for each experimental group/condition, given as a number, not a range;
- a description of the sample collection allowing the reader to understand whether the samples represent technical or biological replicates (including how many animals, litters, cultures, etc.).
- a statement of how many times the experiment shown was independently replicated in the laboratory.
- definitions of statistical methods and measures:
  - common tests, such as t-test (please specify whether paired vs. unpaired), simple χ2 tests, Wilcoxon and Mann-Whitney tests, can be unambiguously identified by name only, but more complex techniques should be described in the methods section;
  - are tests one-sided or two-sided?
  - are there adjustments for multiple comparisons?
  - exact statistical test results, e.g., P values = x but not P values < x;
  - definition of 'center values' as median or average;
  - definition of error bars as s.d. or s.e.m.

Please complete ALL of the questions below.
Select "Not Applicable" only when the requested information is not relevant for your study.

Materials

Newly Created Materials	Information included in the manuscript?	In which section is the information available? (Reagents and Tools Table, Materials and Methods, Figures, Data Availability Section)
New materials and reagents need to be available; do any restrictions apply?	Yes	Data Availability Section

Antibodies	Information included in the manuscript?	In which section is the information available? (Reagents and Tools Table, Materials and Methods, Figures, Data Availability Section)
For **antibodies** provide the following information:<br>- Commercial antibodies: RRID (if possible) or supplier name, catalogue number and or/clone number<br>- Non-commercial: RRID or citation	Yes	Reagents and Tools Table

DNA and RNA sequences	Information included in the manuscript?	In which section is the information available? (Reagents and Tools Table, Materials and Methods, Figures, Data Availability Section)
**Short novel DNA or RNA including primers, probes**: provide the sequences.	Yes	Reagents and Tools Table

Cell materials	Information included in the manuscript?	In which section is the information available? (Reagents and Tools Table, Materials and Methods, Figures, Data Availability Section)
**Cell lines:** Provide species information, strain. Provide accession number in repository **OR** supplier name, catalog number, clone number, and/**OR** RRID.	Yes	Reagents and Tools Table
**Primary cultures:** Provide species, strain, sex of origin, genetic modification status.	Yes	Reagents and Tools Table, Materials and Methods
Report if the cell lines were recently **authenticated** (e.g., by STR profiling) and tested for mycoplasma contamination.	Yes	Materials and Methods

Experimental animals	Information included in the manuscript?	In which section is the information available? (Reagents and Tools Table, Materials and Methods, Figures, Data Availability Section)
**Laboratory animals or Model organisms:** Provide species, strain, sex, age, genetic modification status. Provide accession number in repository **OR** supplier name, catalog number, clone number, **OR** RRID.	Yes	Reagents and Tools Table, Materials and Methods
**Animal observed in or captured from the field:** Provide species, sex, and age where possible.	Not Applicable	
Please detail **housing and husbandry conditions**.	Yes	Materials and Methods

Plants and microbes	Information included in the manuscript?	In which section is the information available? (Reagents and Tools Table, Materials and Methods, Figures, Data Availability Section)
**Plants:** provide species and strain, ecotype and cultivar where relevant, unique accession number if available, and source (including location for collected wild specimens).	Not Applicable	
**Microbes:** provide species and strain, unique accession number if available, and source.	Not Applicable	

Human research participants	Information included in the manuscript?	In which section is the information available? (Reagents and Tools Table, Materials and Methods, Figures, Data Availability Section)
If collected and within the bounds of privacy constraints report on age, sex and gender or ethnicity for all study participants.	Not Applicable	

Core facilities	Information included in the manuscript?	In which section is the information available? (Reagents and Tools Table, Materials and Methods, Figures, Data Availability Section)
If your work benefited from core facilities, was their service mentioned in the acknowledgments section?	Yes	Acknowledgements

Design

Study protocol	Information included in the manuscript?	In which section is the information available? (Reagents and Tools Table, Materials and Methods, Figures, Data Availability Section)
If study protocol has been **pre-registered, provide DOI in the manuscript**. For clinical trials, provide the trial registration number **OR** cite DOI.	Not Applicable	
Report the **clinical trial registration number** (at ClinicalTrials.gov or equivalent), where applicable.	Not Applicable	

Laboratory protocol	Information included in the manuscript?	In which section is the information available? (Reagents and Tools Table, Materials and Methods, Figures, Data Availability Section)
Provide DOI OR other citation details if **external detailed step-by-step protocols** are available.	Not Applicable	

Experimental study design and statistics	Information included in the manuscript?	In which section is the information available? (Reagents and Tools Table, Materials and Methods, Figures, Data Availability Section)
Include a statement about **sample size** estimate even if no statistical methods were used.	Yes	Materials and Methods
Were any steps taken to minimize the effects of subjective bias when allocating animals/samples to treatment (e.g. **randomization procedure**)? If yes, have they been described?	Yes	Materials and Methods
Include a statement about **blinding** even if no blinding was done.	Yes	Materials and Methods
Describe **inclusion/exclusion criteria** if samples or animals were excluded from the analysis. Were the criteria pre-established?<br><br>If sample or data points were omitted from analysis, report if this was due to attrition or intentional exclusion and provide justification.	Not Applicable	
For every figure, are **statistical tests** justified as appropriate? Do the data meet the assumptions of the tests (e.g., normal distribution)? Describe any methods used to assess it. Is there an estimate of variation within each group of data? Is the variance similar between the groups that are being statistically compared?	Yes	Materials and Methods

Sample definition and in-laboratory replication	Information included in the manuscript?	In which section is the information available? (Reagents and Tools Table, Materials and Methods, Figures, Data Availability Section)
In the figure legends: state number of times the experiment was **replicated** in laboratory.	Yes	Figure Legends
In the figure legends: define whether data describe **technical or biological replicates**.	Yes	Figure Legends

Ethics

Ethics	Information included in the manuscript?	In which section is the information available? (Reagents and Tools Table, Materials and Methods, Figures, Data Availability Section)
Studies involving **human participants**: State details of **authority granting ethics approval** (IRB or equivalent committee(s), provide reference number for approval.	Not Applicable	
Studies involving **human participants**: Include a statement confirming that **informed consent** was obtained from all subjects and that the experiments conformed to the principles set out in the WMA Declaration of Helsinki and the Department of Health and Human Services Belmont Report.	Not Applicable	
Studies involving **human participants:** For publication of **patient photos**, include a statement confirming that consent to publish was obtained.	Not Applicable	
Studies involving experimental **animals**: State details of **authority granting ethics approval** (IRB or equivalent committee(s), provide reference number for approval. Include a statement of compliance with ethical regulations.	Yes	Materials and Methods
Studies involving **specimen and field samples:** State if relevant **permits** obtained, provide details of authority approving study; if none were required, explain why.	Not Applicable	

Dual Use Research of Concern (DURC)	Information included in the manuscript?	In which section is the information available? (Reagents and Tools Table, Materials and Methods, Figures, Data Availability Section)
Could your study fall under dual use research restrictions? Please check biosecurity documents and list of **select agents and toxins** (CDC): https://www.selectagents.gov/sat/list.htm	Not Applicable	
If you used a select agent, is the security level of the lab appropriate and reported in the manuscript?	Not Applicable	
If a study is subject to dual use research of concern regulations, is the name of the **authority granting approval and reference number** for the regulatory approval provided in the manuscript?	Not Applicable	

Reporting

The MDAR framework recommends adoption of discipline-specific guidelines, established and endorsed through community initiatives. Journals have their own policy about requiring specific guidelines and recommendations to complement MDAR.

Adherence to community standards	Information included in the manuscript?	In which section is the information available? (Reagents and Tools Table, Materials and Methods, Figures, Data Availability Section)
State if relevant guidelines or checklists (e.g., **ICMJE, MIBBI, ARRIVE, PRISMA**) have been followed or provided.	Yes	Materials and Methods
For **tumor marker prognostic studies,** we recommend that you follow the **REMARK** reporting guidelines (see link list at top right). See author guidelines, under 'Reporting Guidelines'. Please confirm you have followed these guidelines.	Not Applicable	
For **phase II and III randomized controlled trials**, please refer to the **CONSORT** flow diagram (see link list at top right) and submit the CONSORT checklist (see link list at top right) with your submission. See author guidelines, under 'Reporting Guidelines'. Please confirm you have submitted this list.	Not Applicable	

Data Availability

Data availability	Information included in the manuscript?	In which section is the information available? (Reagents and Tools Table, Materials and Methods, Figures, Data Availability Section)
Have **primary datasets** been deposited according to the journal's guidelines (see 'Data Deposition' section) and the respective accession numbers provided in the Data Availability Section?	Yes	Data Availability
Were **human clinical and genomic datasets** deposited in a public access-controlled repository in accordance to ethical obligations to the patients and to the applicable consent agreement?	Not Applicable	
Are **computational models** that are central and integral to a study available without restrictions in a machine-readable form? Were the relevant accession numbers or links provided?	Not Applicable	
If publicly available data were reused, provide the respective **data citations in the reference list**.	Yes	Materials and Methods
