# Supplementary material for: CDK8 remodels the tumor microenvironment and promotes resistance to KRASG12D inhibitors and daraxonrasib in PDAC
Source: EMBO J. 2026 Jul 11;45(16):5727–58. doi: 10.1038/s44318-026-00854-5 (PMC13483915; doi:10.1038/s44318-026-00854-5)
Supplement: Supplementary file 2 [file 44318_2026_854_MOESM3_ESM.pdf]

Appendix

CDK8 remodels tumor microenvironment to resist $KRAS^{G12D}$ inhibitors and daraxonrasib in PDAC

Kathleen M. McAndrews[1], Krishnan K. Mahadevan[1], Bingrui Li[1], Anaïs M. Comptdaer[1], Amari M. Sockwell[1], Sami J. Morse[1], Patience J. Kelly[1], Sarah I. Patel[1], Michelle L. Kirtley[1], Barbara A. Moreno Diaz[1], Hengyu Lyu[2], David H. Peng[2], Sima Khazaei[2], Aislyn Schalck[2], Xunian Zhou[1], Hikaru Sugimoto[1], Aaron A. Bickert[1], Lakshmi Kavitha Sthanam[1], Meagan R. Taylor[1], Shreyasee V. Kumbhar[1], Kent A. Arian[1], Yasaman Barekatain[1], Francesca Paradiso[3], Paola A. Guerrero[3], Vincent Bernard[4], Navid Sobhani[1], Alondra N. Camacho-Acevedo[1], Kiara E. Bornes[1], Phuong Thao Tran[1], Anirban Maitra[3,5], Timothy P. Heffernan[2] and Raghu Kalluri[1,6,7,8,*]

[1]Department of Cancer Biology, Metastasis Research Center, University of Texas MD Anderson Cancer Center, Houston, TX 77030

[2]TRACTION Platform, Therapeutics Discovery Division, The University of Texas MD Anderson Cancer Center, Houston, TX 77030

[3]Department of Translational Molecular Pathology, Sheikh Ahmed Center for Pancreatic Cancer Research, The University of Texas MD Anderson Cancer Center, Houston, TX 77030

[4]Department of Radiation Oncology, The University of Texas MD Anderson Cancer Center, Houston, TX 77030

[5]Department of Pathology, The University of Texas MD Anderson Cancer Center, Houston, TX 77030

[6]Department of Bioengineering, Rice University, Houston, TX 77005

[7]Department of Molecular and Cellular Biology, Baylor College of Medicine, Houston, TX 77030

[8]Department of Pathology, University of Texas Medical Branch, Galveston, TX 77555

Appendix Table of Contents

Appendix Figures S1-15

Appendix Figure S1

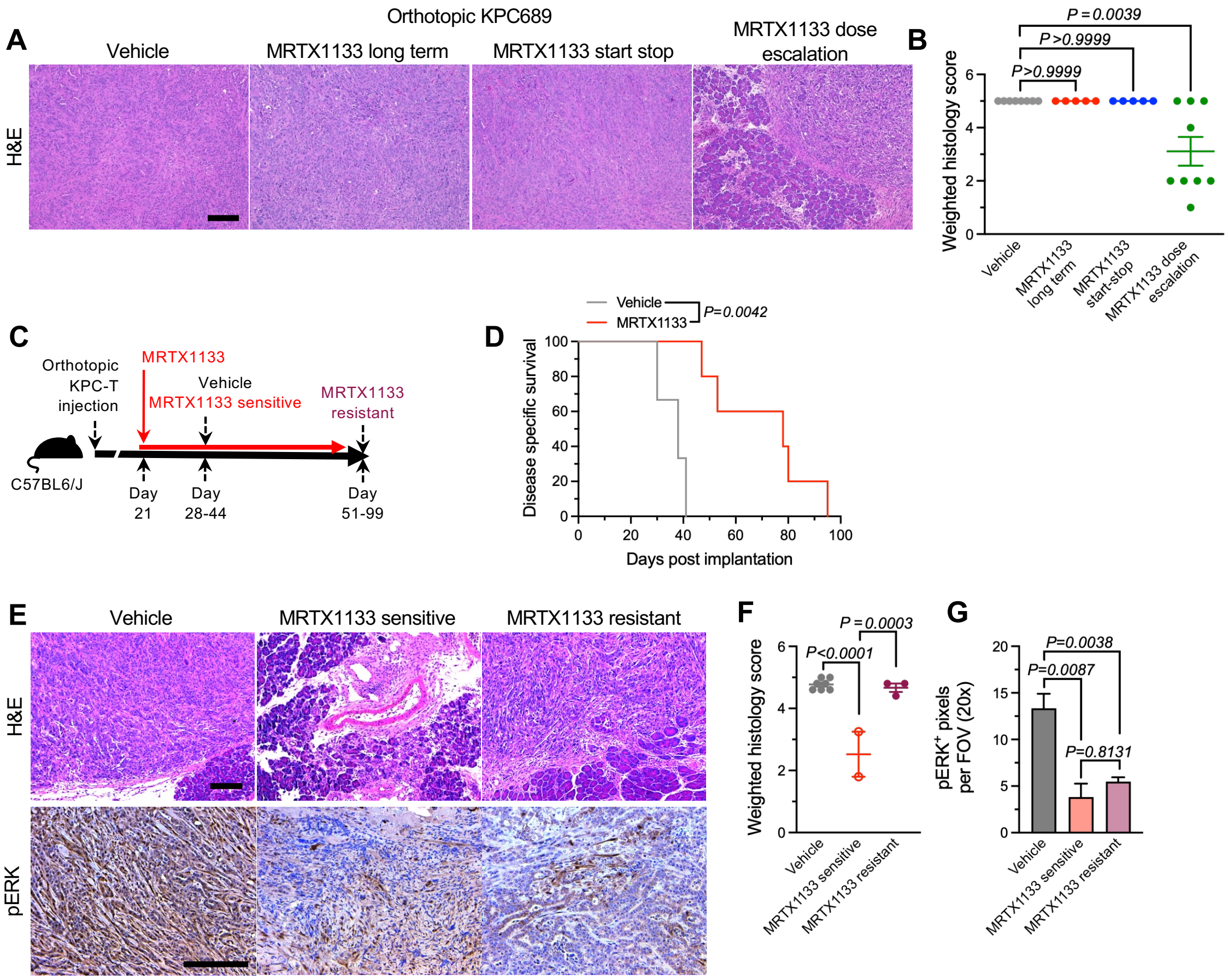

Appendix Figure S1: Response of orthotopic tumors to MRTX1133. (**A-B**) Representative H&E images (**A**) and quantification (**B**) of orthotopic KPC689 tumors. Vehicle: n=8, MRTX1133 long term: n=5, MRTX1133 start-stop: n=5, MRTX1133 dose escalation: n=9. (**C**) Schematic of orthotopic KPC-T tumors treated with MRTX1133. (**D**) Survival curve of orthotopic KPC-T mice. Vehicle, n=3, MRTX1133, n=5. Note: Same mice from Fig. 5E are repeated here to allow for direct comparisons. (**E-G**) Representative H&E images (**E**) and quantification (**F**) of orthotopic KPC-T tumors. Vehicle, n=7; MRTX1133 sensitive, n=2; MRTX1133 resistant, n=3. Representative pERK staining (**E**) and quantification (**G**) of orthotopic KPC-T tumors. Vehicle, n=7, MRTX1133 sensitive, n=2, MRTX1133 resistant, n=5. Kruskal-Wallis ANOVA with Dunn's multiple comparison test

performed in (B). Log-rank test performed in (D). One-way ANOVA with Tukey's multiple comparisons test performed in (F) and (G). Scale bars, 100 μm. Data are presented as mean ± s.e.m. Exact p-values reported.

Appendix Figure S2

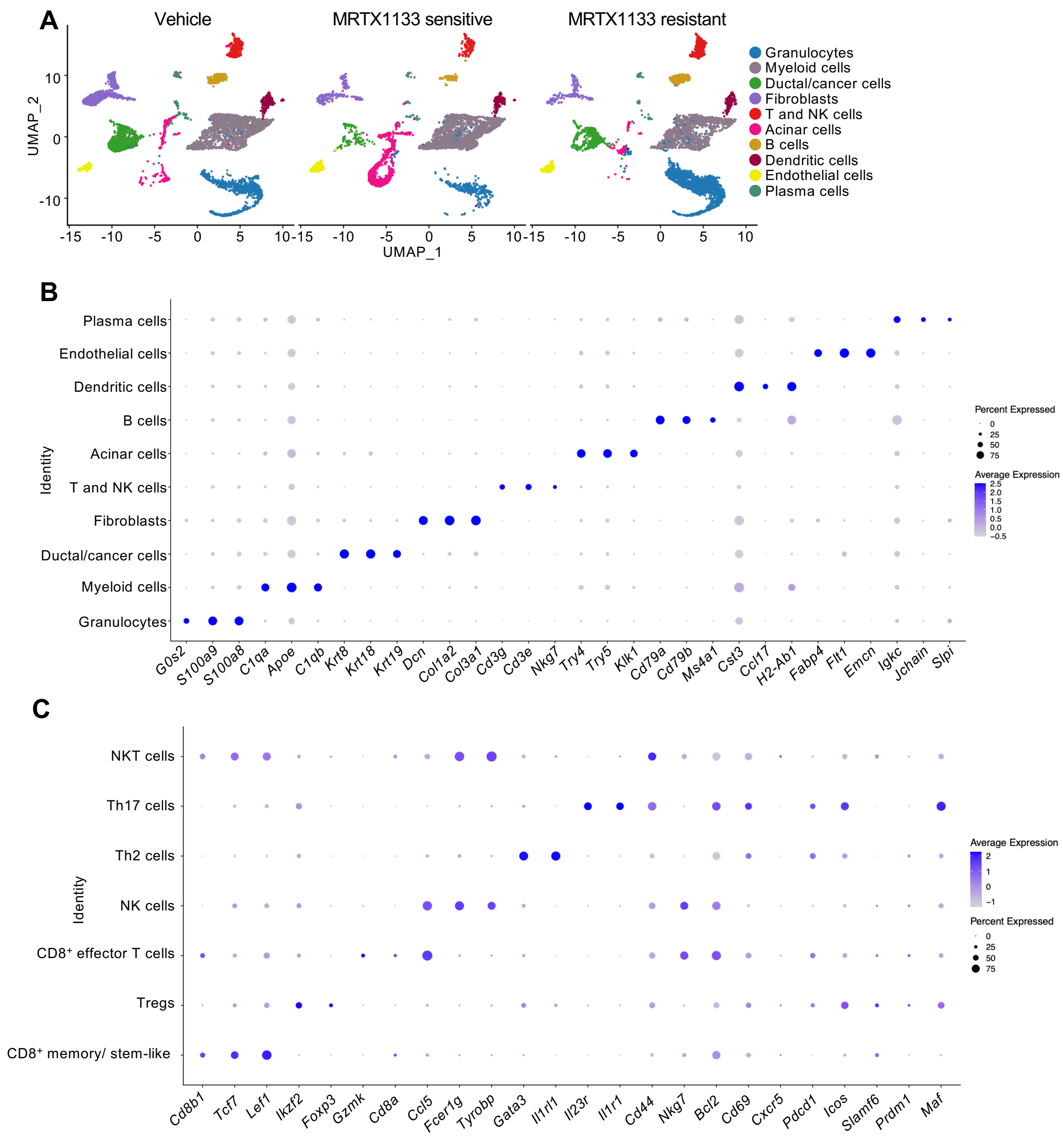

Appendix Figure S2: Cell type annotations based on scRNA-seq of orthotopic PKC-HY19636 tumors. (**A-C**) UMAPs (**A**) of orthotopic PKC-HY19636 tumors determined by

scRNA-seq. Dot plot of genes used to define general cell types (**B**), T and NK cell subtypes (**C**) of orthotopic PKC-HY19636 tumors determined by scRNA-seq.

Appendix Figure S3

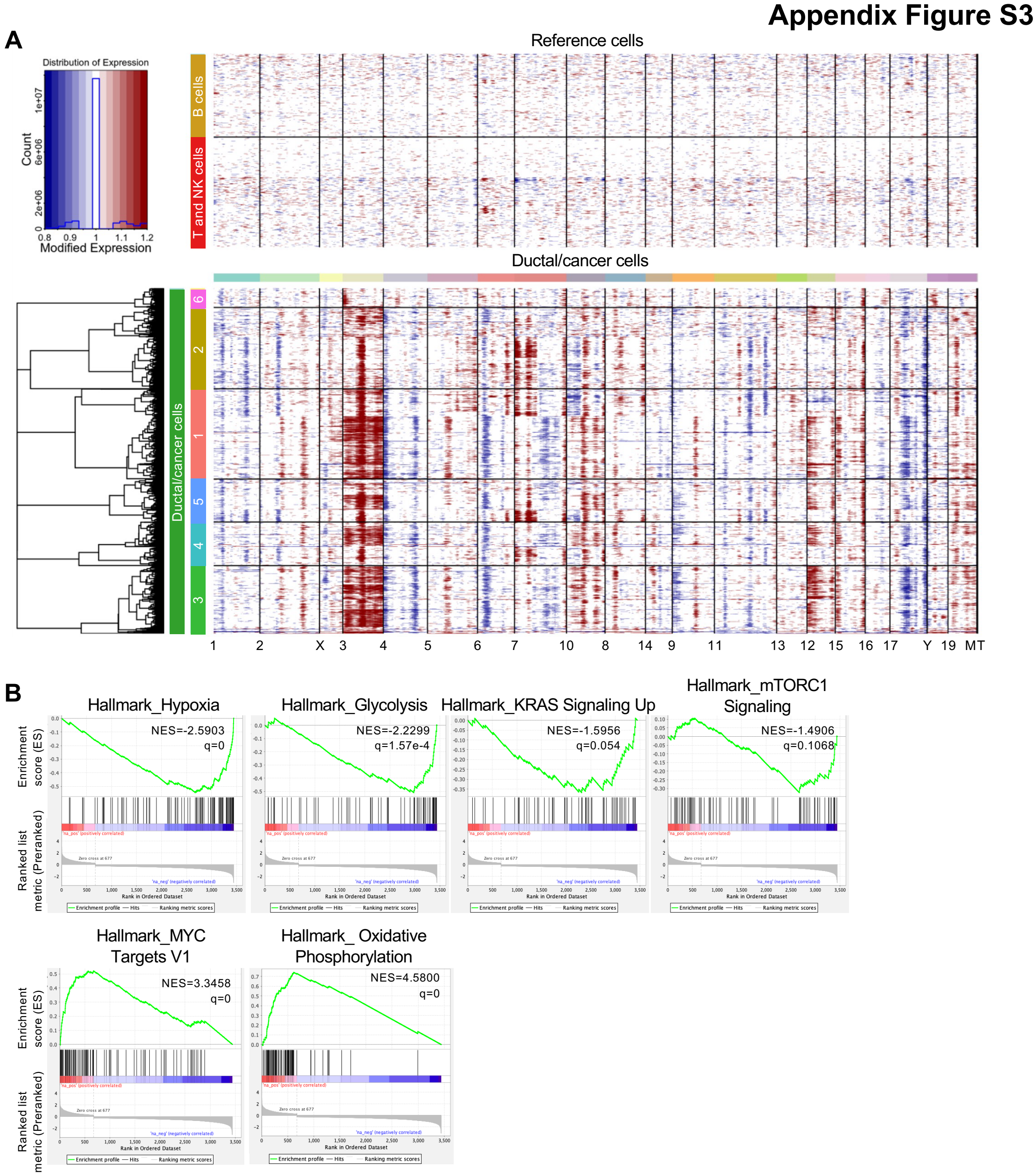

Appendix Figure S3: InferCNV analysis and enriched pathways of ductal/cancer cells based on scRNA-seq of orthotopic PKC-HY19636 tumors. (**A**) InferCNV

analysis of the ductal/cancer cell cluster of PKC-HY19636 tumors defined in Fig. 3A-B and Supplemental Fig. 2A-B. The T cell and B cell clusters were used as a reference. Ductal/cancer cell clusters match the clusters defined in Fig. 3B. (**B**) GSEA of pathways enriched in ductal/cancer cells of MRTX1133 resistant orthotopic PKC-HY19636 tumors.

Appendix Figure S4

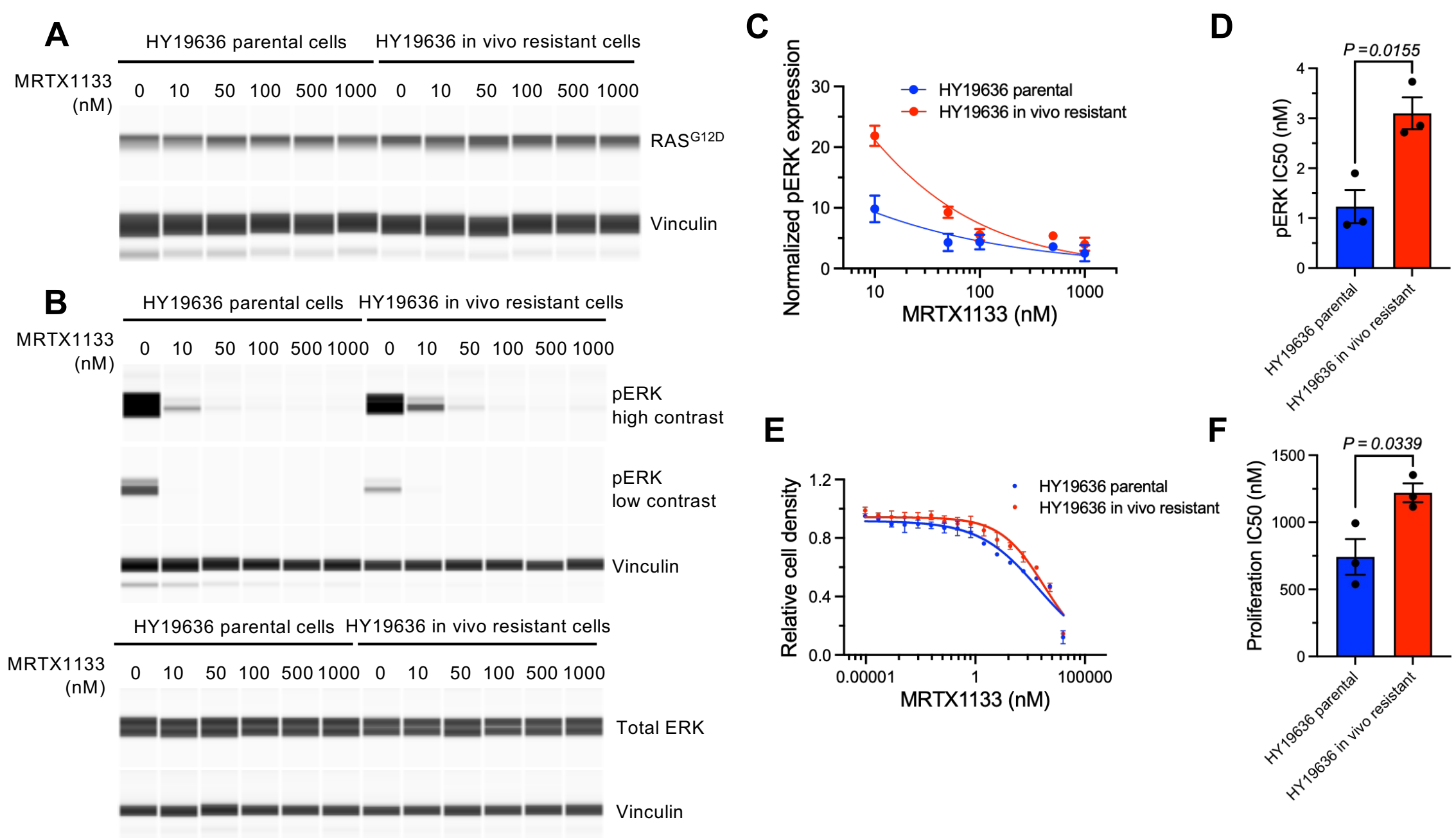

Appendix Figure S4: MRTX1133 resistant cells isolated from tumors demonstrate increased pERK and proliferation in response to MRTX1133 in vitro. (**A**) Simple Western immunoassay for RAS^{G12D} and vinculin of HY19636 parental and HY19636 in vivo resistant cells treated with the indicated concentrations of MRTX1133 for 3 hours. (**B**) Simple Western immunoassay for pERK, total ERK, and vinculin of HY19636 parental and HY19636 in vivo resistant cells treated with the indicated concentrations of MRTX1133 for 3 hours. A low contrast and high contrast image of the same gel are shown to better visualize samples with low pERK expression. (**C-D**) pERK expression

normalized to vinculin and the vehicle treated (0 nM) control for each cell line (**C**) and quantification of pERK IC50 (**D**). n=3 independent experiments. (**E-F**) Relative cell density of HY19636 parental and HY19636 in vivo resistant cells treated with the indicated concentrations of MRTX1133 for 3 days (**E**) and quantification of proliferation IC50 (**F**). n=3 independent experiments. Unpaired two-tailed t-test performed in (D) and (F). Data are presented as mean ± s.e.m. Exact p-values reported.

Appendix Figure S5

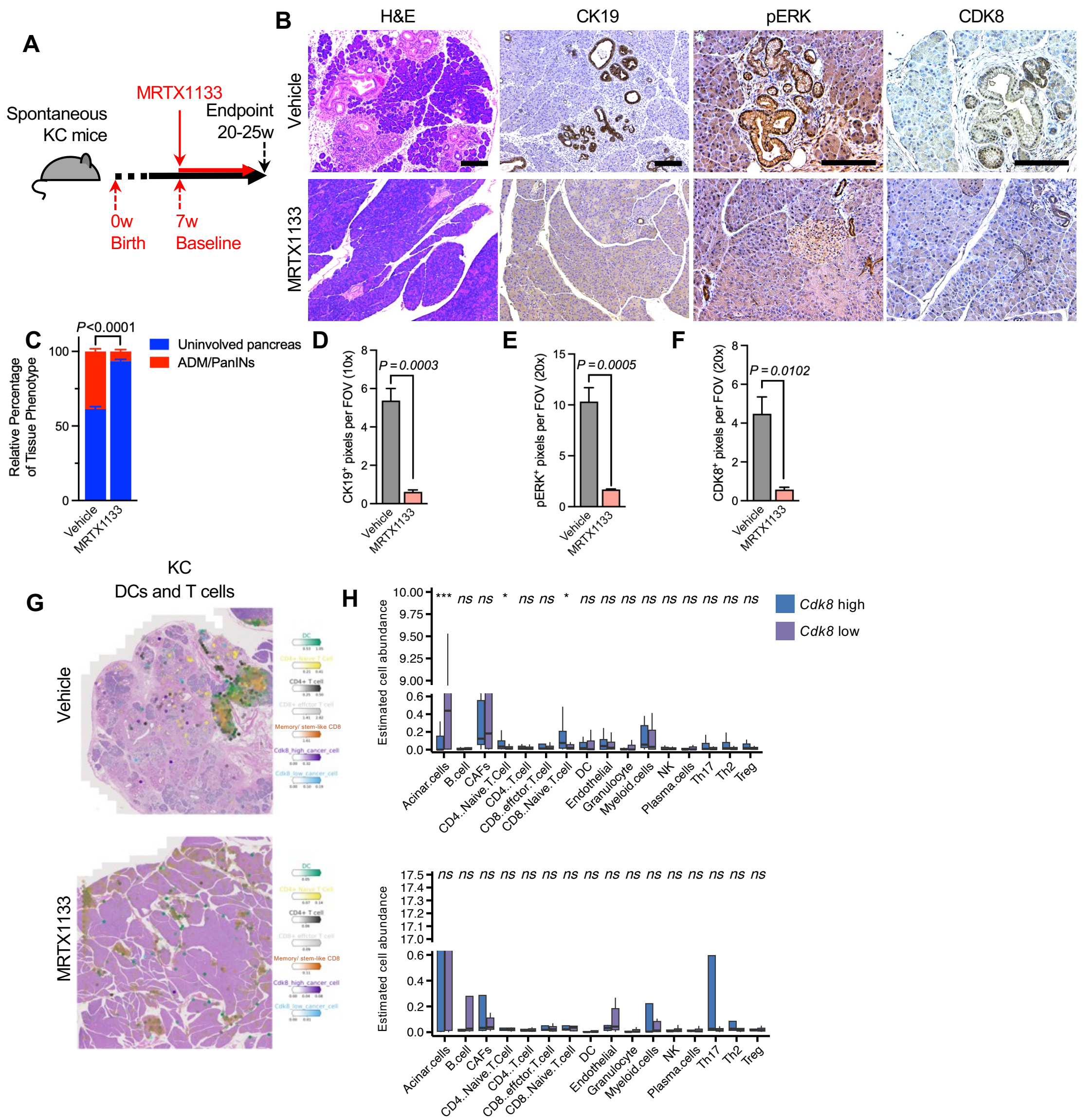

Appendix Figure S5: Response of KC pancreata to MRTX1133. (**A**) Schematic of spontaneous KC mice treated with MRTX1133. (**B-F**) Representative H&E, CK19, pERK, and CDK8 images (**B**) and quantification (**C-F**) of KC pancreata. C: Vehicle, n=2; MRTX1133, n=4. D: Vehicle, n=2; MRTX1133, n=4. E: Vehicle, n=2; MRTX1133, n=4. F: Vehicle, n=5; MRTX1133, n=5. (**G-H**) Spatial transcriptomics representative images (**G**) and quantification (**H**) of KC pancreata. Spots were stratified by *Cdk8* high and low areas.

Scale bar, 100 µm. Two-sided Mann-Whitney test was performed in (H). Two-way ANOVA with Sidak's multiple comparisons test performed in (C). Unpaired two-tailed t-test performed in (D) and (E). Unpaired two-tailed t-test with Welch's correction performed in (F). Data are presented as mean ± s.e.m. except for in (H) where it is presented as median and range. * $P < 0.05$, *** $P < 0.001$, *ns*: not significant, or exact p-values reported.

Appendix Figure S6

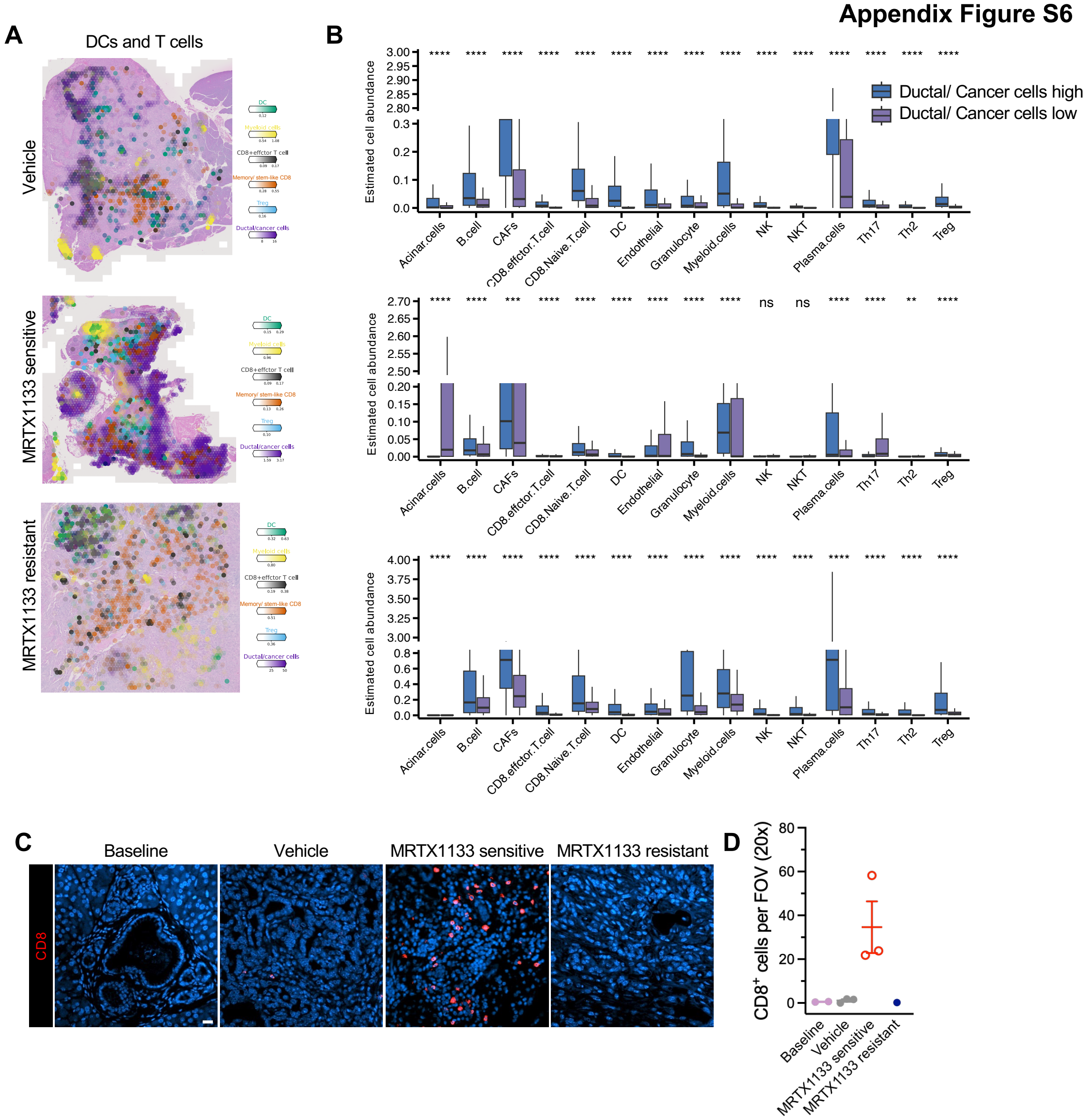

Appendix Figure S6: MRTX1133 resistance alters the tumor microenvironment. (**A-B**) Spatial transcriptomics of orthotopic PKC-HY19636 tumors. Representative images (**A**) and quantification of cell abundance (**B**). Spots were stratified by ductal/cancer cell high and low areas. Vehicle, n=3; MRTX1133 sensitive, n=3; MRTX1133 resistant, n=3. (**C-D**) Representative images (**C**) and quantification (**D**) of CD8 immunostaining (red) of KPCY tumors. Baseline, n=2; vehicle, n=3; MRTX1133 sensitive, n=3; MRTX1133

resistant, n=1. Scale bar, 20 μm. Two-sided Mann-Whitney test was performed in (B). Data are presented as mean ± s.e.m. except for in (B) where it is presented as median and range. * $P < 0.05$, ** $P < 0.01$, **** $P < 0.0001$, *ns*: not significant.

Appendix Figure S7

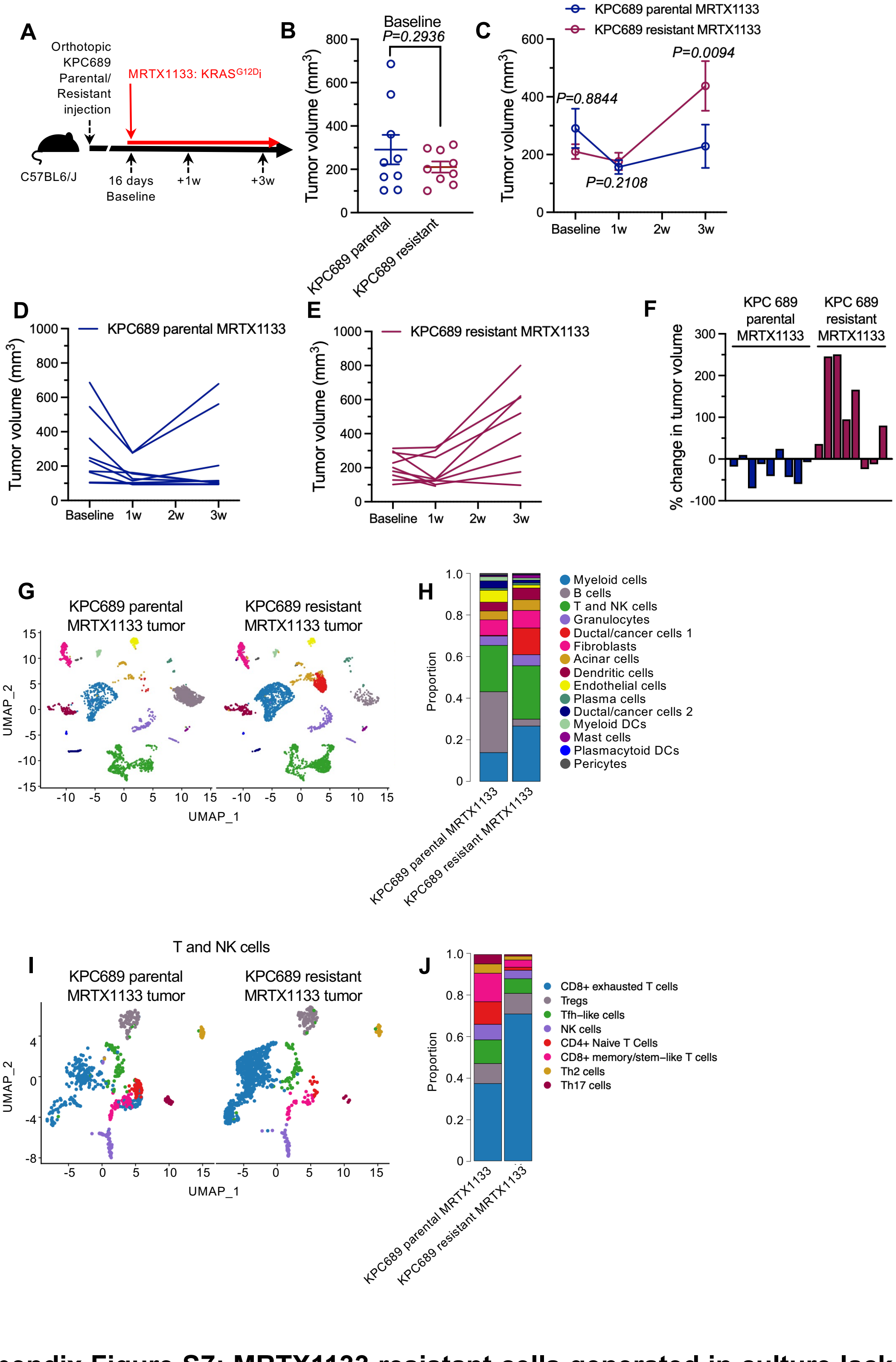

Appendix Figure S7: MRTX1133 resistant cells generated in culture lack response

to MRTX1133 in vivo and remodel the TME. (**A**) Schematic of orthotopic KPC689 parental and KPC689 resistant tumors treated with MRTX1133. (**B-F**) Quantified MRI tumor volume at baseline (**B**) and following MRTX1133 treatment (**C**). Individual tumor volume curves for KPC689 parental (**D**), KPC689 resistant (**E**), and percent change in tumor volume at 3 weeks compared to baseline (**F**). KPC689 parental MRTX1133, n=9; KPC689 resistant MRTX1133, n=9. (**G-H**) UMAP (**G**) and relative proportions of all cells (**H**) of orthotopic KPC689 parental and resistant tumors treated with MRTX1133 determined by scRNA-seq**.** (**I-J**) UMAP (**I**) and relative proportions of T and NK cells (**J**) of orthotopic KPC689 parental and resistant tumors treated with MRTX1133 determined by scRNA-seq**.** Pancreata/tumors from 3 mice pooled per group. *ns*: not significant. Mixed-effects analysis with Bonferroni's multiple comparisons test performed in (C) and unpaired two-tailed t-test with Welch's correction performed in (B). Exact p-values reported.

Appendix Figure S8

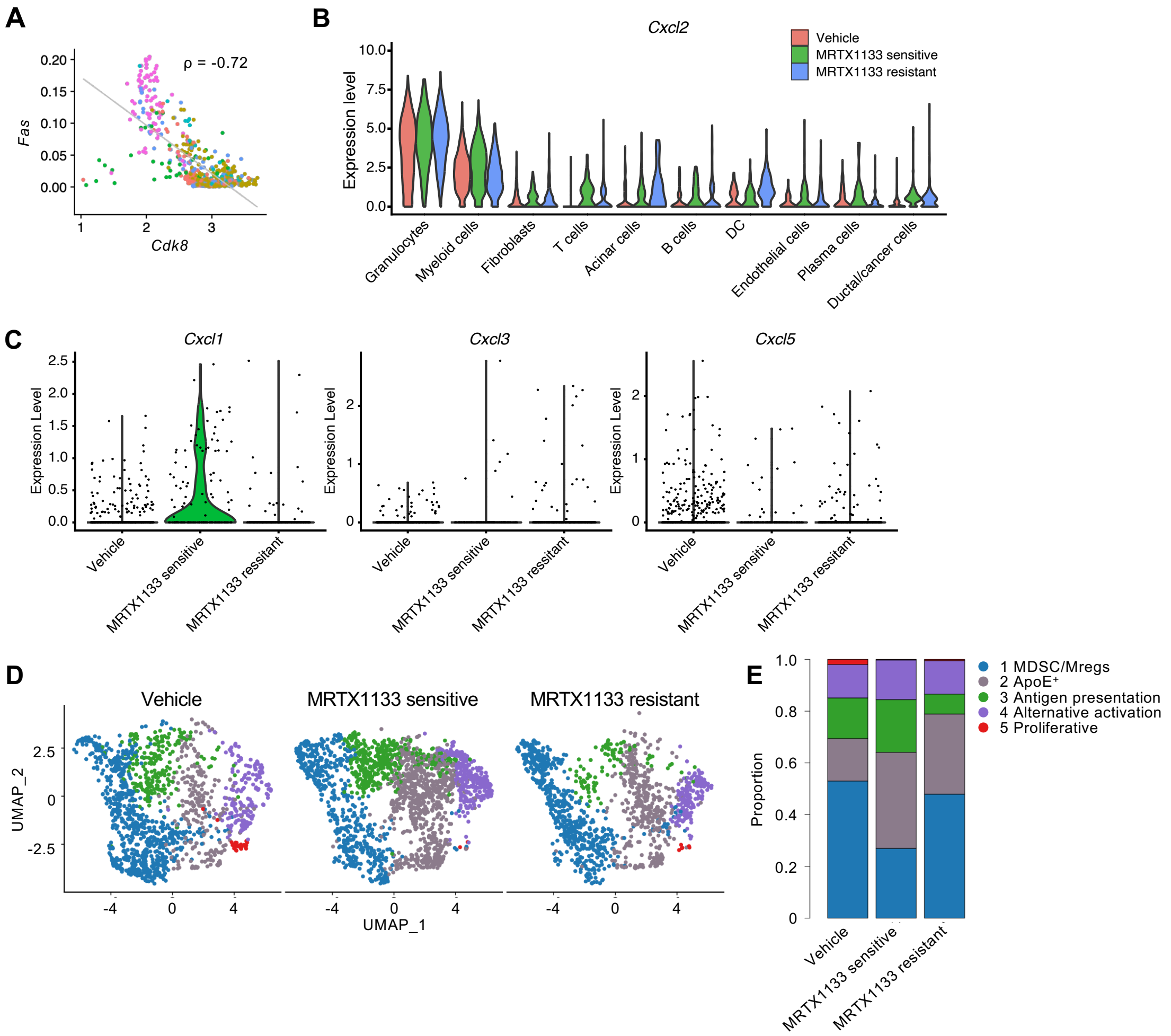

Appendix Figure S8: Myeloid infiltrate of MRTX1133 resistant tumors. (**A**) Correlation between *Fas* and *Cdk8* expression based on scRNA-seq analysis. (**B**) *Cxcl2* expression in the expression of the indicated cell clusters of orthotopic PKC-HY19636 tumors determined by scRNA-seq. (**C**) Violin plots of *Cxcl1, Cxcl3, and Cxcl5* in ductal/cancer cells of orthotopic PKC-HY19636 tumors determined by scRNA-seq. (**D-E**) UMAP (**D**) and relative proportions of myeloid cells (**E**) of orthotopic PKC-HY19636 tumors determined by scRNA-seq. Data are reported as exact values except for in (B) where they are reported as violin plots.

Appendix Figure S9

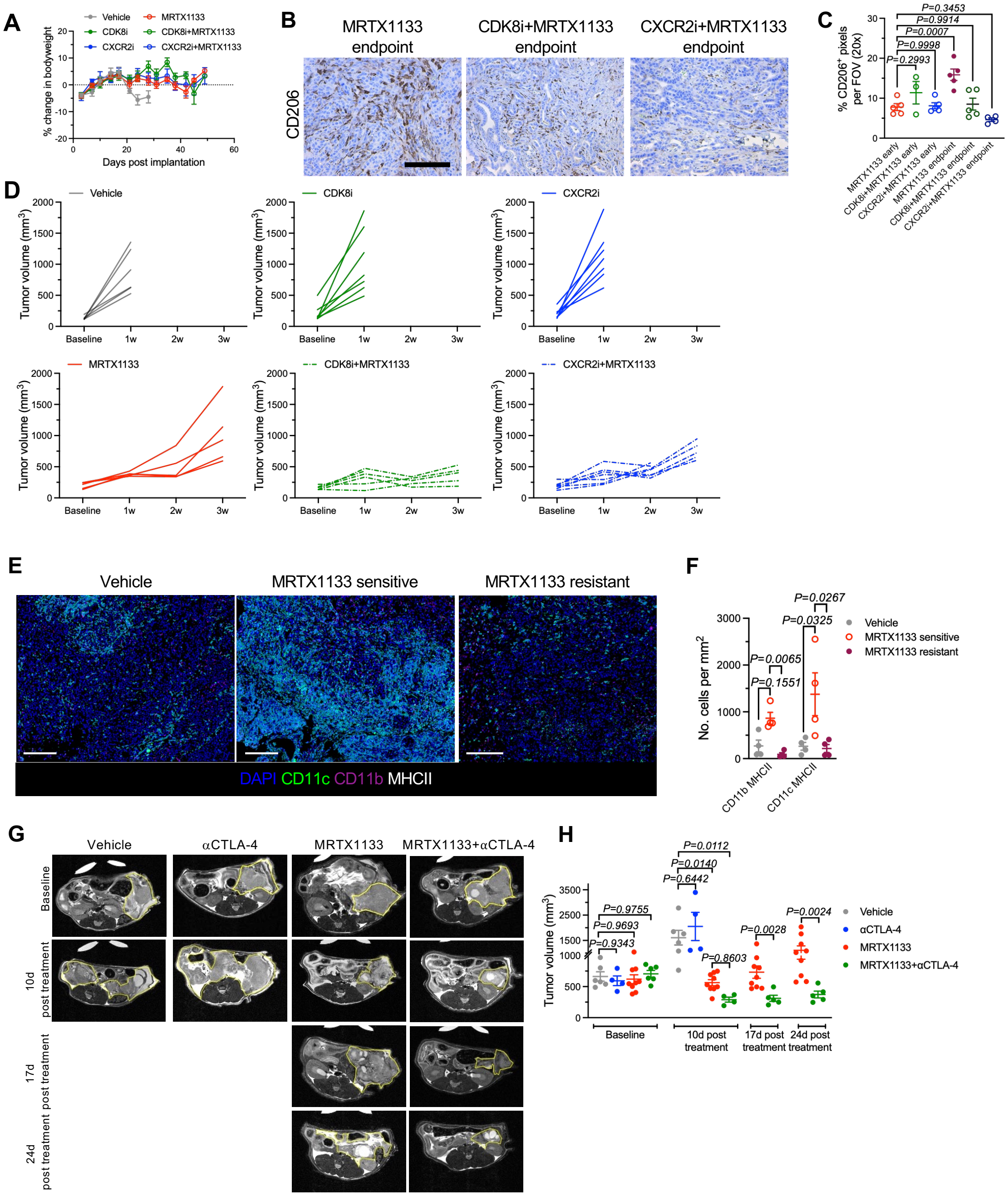

Appendix Figure S9: Analysis of immune infiltration and MRI analysis of PDAC mice. (**A**) Bodyweight of mice bearing orthotopic PKC-HY19636 tumors post tumor-implantation. Vehicle, n=12; CDK8i, n=12; CXCR2i, n=12; MRTX1133, n=13; CDK8i+MRTX1133, n=12; CXCR2i+MRTX1133, n=13. (**B-C**) Representative CD206

staining (**B**) and quantification of orthotopic PKC-HY19636 tumors at endpoint (**C**). MRTX1133 early, n=5; CDK8i+MRTX1133 early, n=3; CXCR2i+MRTX1133 early, n=5; MRTX1133 endpoint, n=5; CDK8i+MRTX1133 endpoint, n=5; CXCR2i+MRTX1133 endpoint, n=4. Scale bar, 100 μm. Data from Fig. 5H are presented again here to directly compare early vs. endpoint tumors. (**D**) Tumor volumes measured by MRI of orthotopic PKC-HY19636 tumors treated with MRTX1133, CDK8i, and CXCR2i. Vehicle, n=6; CDK8i, n=7; CXCR2i, n=7; MRTX1133, n=5; CDK8i+MRTX1133, n=6; CXCR2i+MRTX1133, n=7. (**E-F**) Representative CODEX images of CD11c, CD11b, and MHCII (**E**) and quantification of cell abundance per tissue area of PKC-HY19636 orthotopic tumors (**F**). Vehicle, n=4; MRTX1133 sensitive, n=4; MRTX1133 resistant, n=4. (**G-H**) Representative images (**G**) and quantified tumor volume measured by MRI relative to tumor volume at 2 weeks following MRTX1133 treatment initiation (**H**). Yellow lines demarcate tumor regions. Vehicle, n=6; αCTLA-4, n=4; MRTX1133, n=9; MRTX1133+αCTLA-4, n=6. Kruskal-Wallis with Dunn's multiple comparisons test performed for (C), CD11b MHCII in (F), one-way ANOVA with Dunnett's multiple comparisons test performed for CD11c MHCII in (F), one-way ANOVA with Tukey's multiple comparisons for baseline and 10d post-treatment comparisons in (H), and unpaired t-test with Welch's correction performed for other comparisons in (H). Data are presented as mean ± s.e.m. except in (D) where individual mice are plotted. Exact p-values reported.

Appendix Figure S10

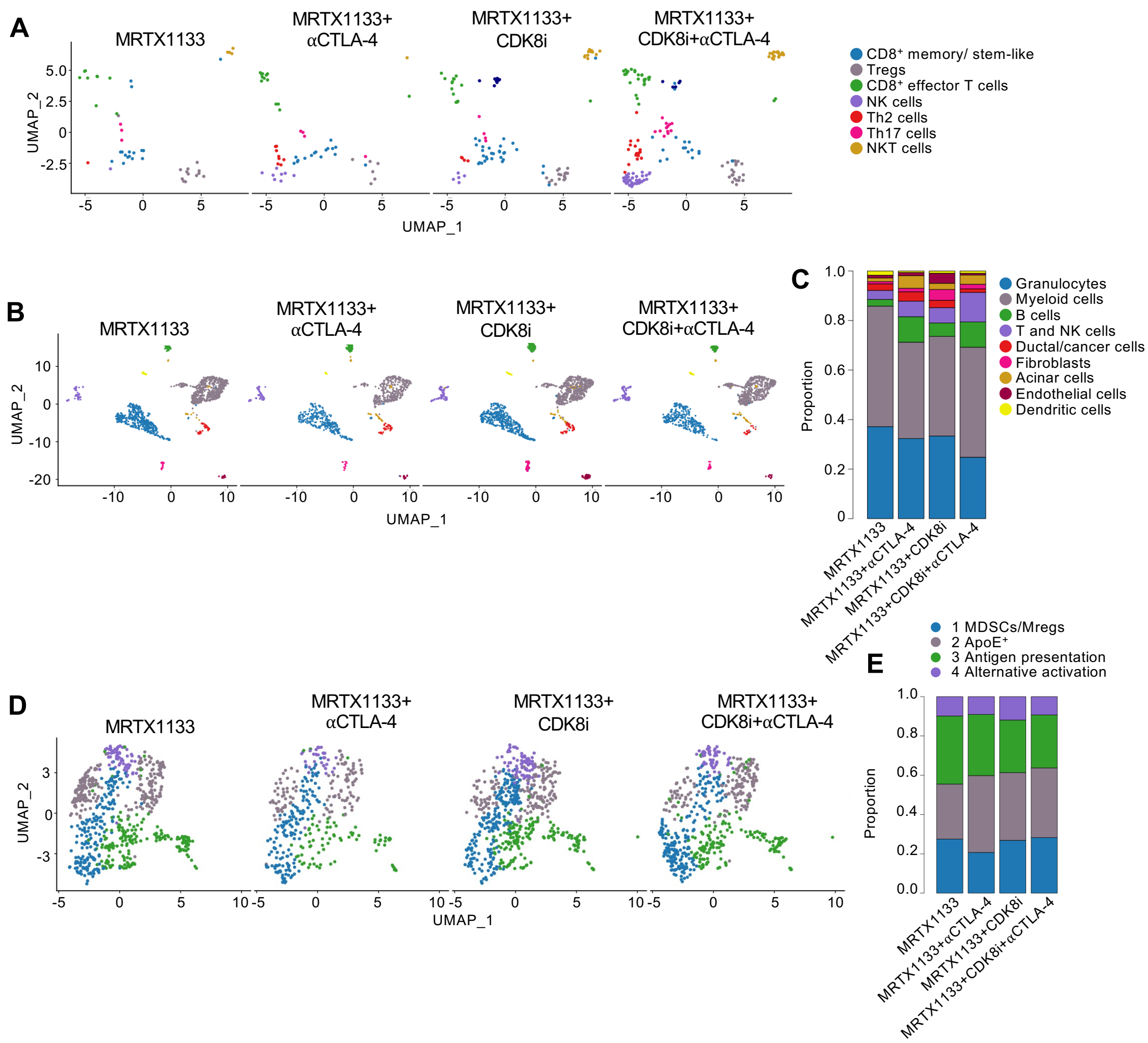

Appendix Figure S10: T cell and myeloid cell infiltration in MRTX1133 resistant tumors treated with CDK8i with αCTLA-4. (**A**) UMAP of T cell subsets of PKC-HY19636 tumors analyzed by scRNA-seq. (**B-C**) UMAP (**B**) and relative proportions of cells (**C**) of orthotopic PKC-HY19636 tumors treated with MRTX1133, CDK8i, and αCTLA-4 determined by scRNA-seq. (**D-E**) UMAP (**D**) and relative proportions (**E**) of myeloid subsets in orthotopic PKC-HY19636 tumors analyzed by scRNA-seq. Data are reported as exact values (C) and (E).
