# Supplementary material for: CDK8 remodels the tumor microenvironment and promotes resistance to KRASG12D inhibitors and daraxonrasib in PDAC
Source: EMBO J. 2026 Jul 11;45(16):5727–58. doi: 10.1038/s44318-026-00854-5 (PMC13483915; doi:10.1038/s44318-026-00854-5)

Uncropped blots

Appendix Figure S4A

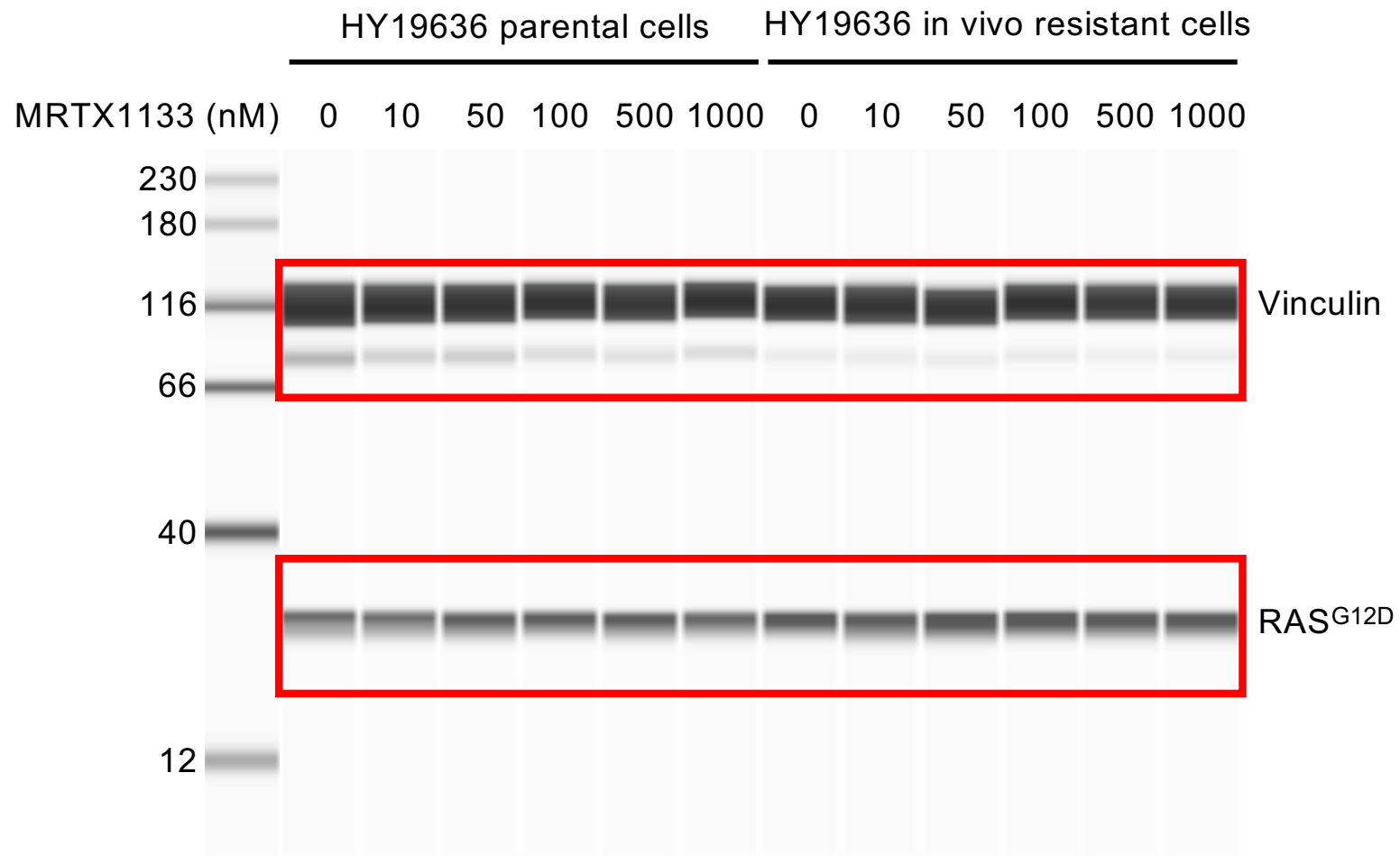

Appendix Figure S4B

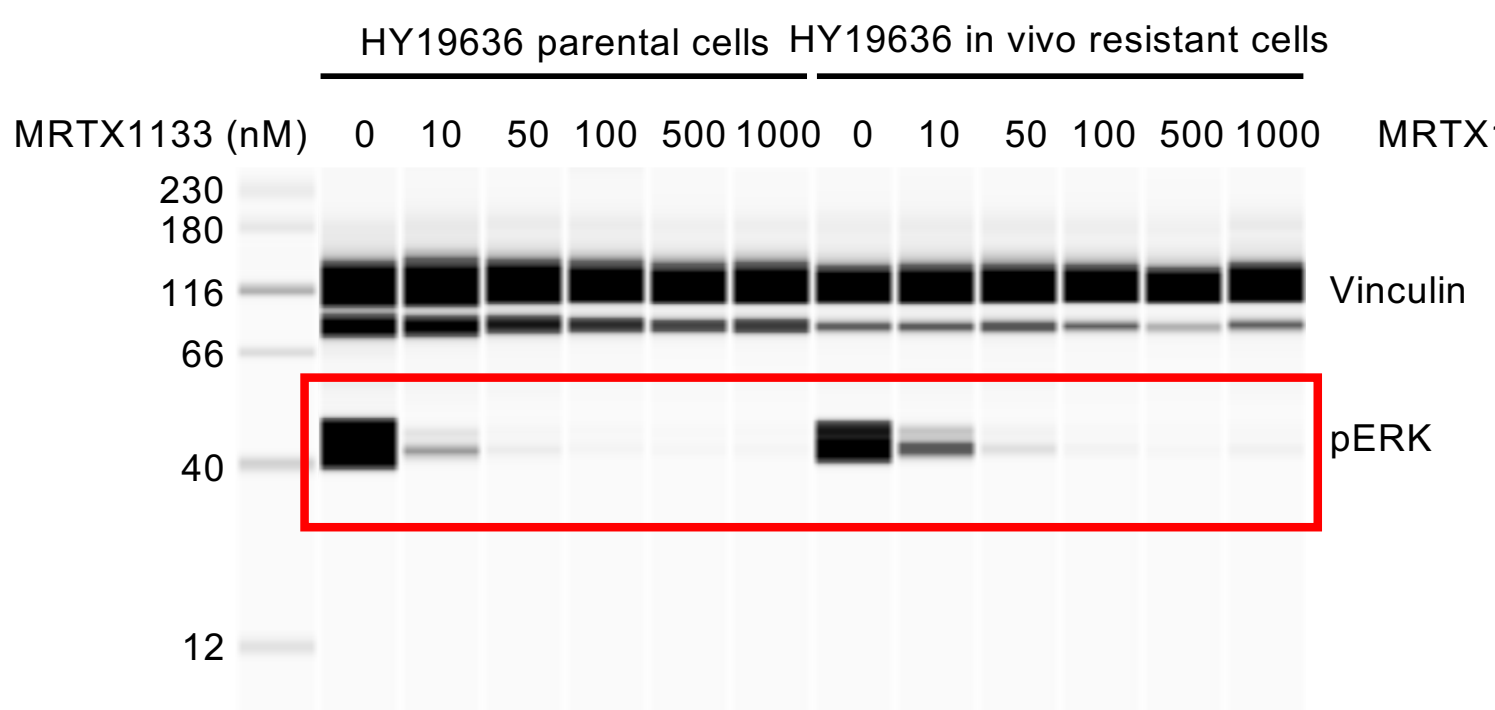

High contrast

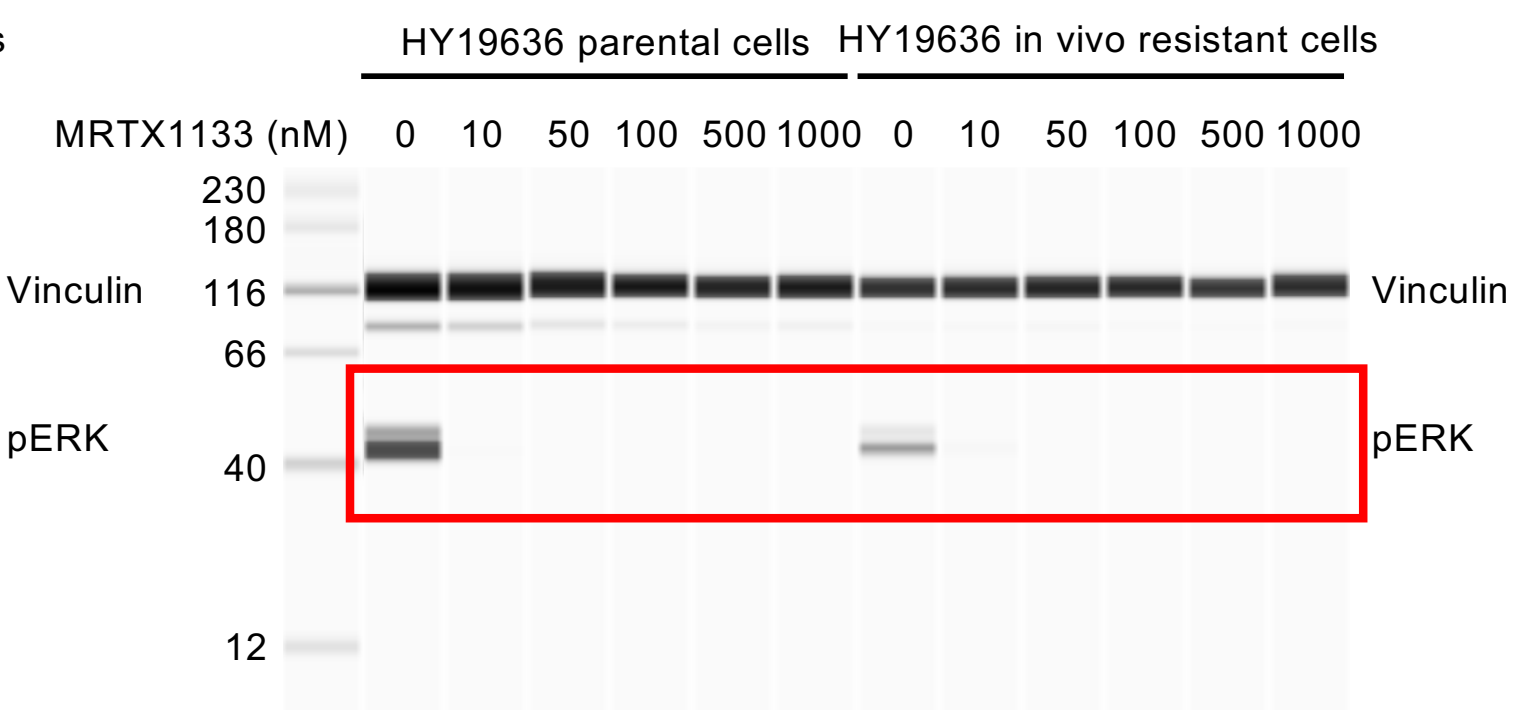

Low contrast

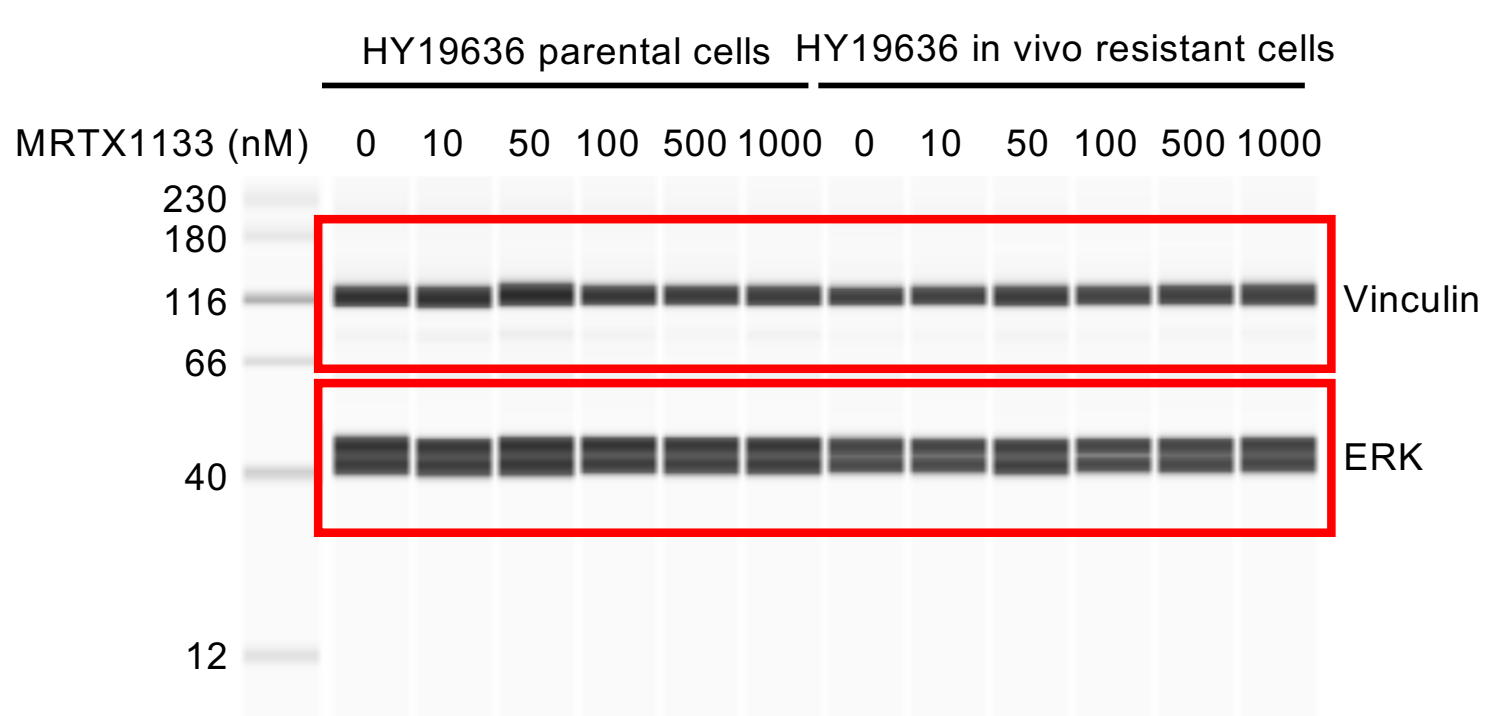

Supplement: Supplementary file 4 [file 44318_2026_854_MOESM5_ESM.pdf]
