# Supplementary material for: CDK8 remodels the tumor microenvironment and promotes resistance to KRASG12D inhibitors and daraxonrasib in PDAC
Source: EMBO J. 2026 Jul 11;45(16):5727–58. doi: 10.1038/s44318-026-00854-5 (PMC13483915; doi:10.1038/s44318-026-00854-5)
Supplement: Supplementary file 5 [file 44318_2026_854_MOESM6_ESM.pdf]

Expanded View Figures

Figure EV1. Anti-PDAC responses of spontaneous and orthotopic KPC models to MRTX1133.

(**A**, **B**) Representative immunostaining images (**A**) and quantification of $CK19^+$, $CK19^+pERK^+$, and $CK19^-pERK^+$ cells in orthotopic PKC-HY19636 tumors (**B**). Vehicle, $n = 4$; MRTX1133 sensitive, $n = 3$; MRTX1133 resistant, $n = 4$. Scale bar, 20 µm. (**C**) MRI of spontaneous KPCY models. Yellow lines demarcate tumor regions. (**D**) Bodyweight of KPCY mice during treatment. Vehicle, $n = 3$; MRTX1133 sensitive, $n = 4$; MRTX1133 resistant, $n = 1$. (**E**) Representative images of EYFP lineage tracer stains of spontaneous KPCY tumors. Scale bar, 100 µm. (**F**, **G**) Representative bioluminescence images (**F**) and individual bioluminescence curves (**G**) of KPC689 tumors. Vehicle: $n = 8$, MRTX1133 long term: $n = 9$, MRTX1133 start-stop: $n = 8$, MRTX1133 dose escalation: $n = 9$. (**H**) Bodyweight of mice bearing orthotopic KPC689 tumors post tumor-implantation. Vehicle: $n = 8$, MRTX1133 long term: $n = 9$, MRTX1133 start-stop: $n = 8$, MRTX1133 dose escalation: $n = 9$. Data are presented as mean ± s.e.m. except in (**D**, **G**) where individual mice are plotted. One-way ANOVA with Sidak's multiple comparisons test performed in (**B**). Exact *P* values reported.

© The Author(s)

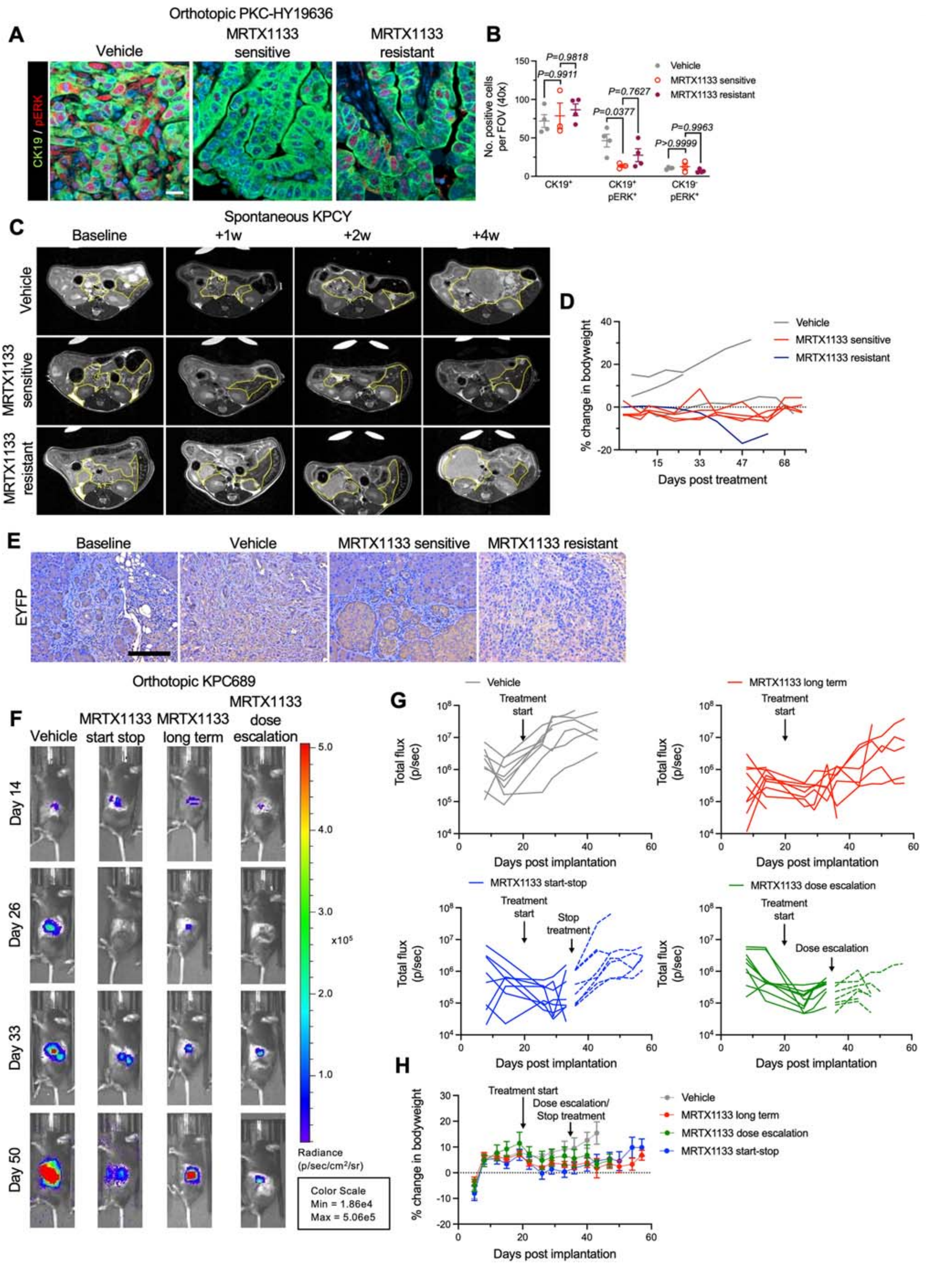
Orthotopic PKC-HY19636
A
Vehicle
MRTX1133 sensitive
MRTX1133 resistant
CK19 / pERK
B
No. positive cells per FOV (40x)
Vehicle
MRTX1133 sensitive
MRTX1133 resistant
P=0.9818
P=0.9911
P=0.7627
P=0.0377
P=0.9963
P>0.9999
CK19+
CK19+ pERK+
CK19- pERK+
Spontaneous KPCY
C
Baseline
+1w
+2w
+4w
Vehicle
MRTX1133 sensitive
MRTX1133 resistant
D
% change in bodyweight
Days post treatment
E
EYFP
Orthotopic KPC689
F
Vehicle
MRTX1133 start stop
MRTX1133 long term
MRTX1133 dose escalation
Day 14
Day 26
Day 33
Day 50
Radiance (p/sec/cm²/sr)
x10⁵
Color Scale
Min = 1.86e4
Max = 5.06e5
G
MRTX1133 long term
MRTX1133 start-stop
MRTX1133 dose escalation
Treatment start
Stop treatment
Dose escalation
Total flux (p/sec)
Days post implantation
H
Treatment start
Dose escalation/ Stop treatment
% change in bodyweight
Days post implantation

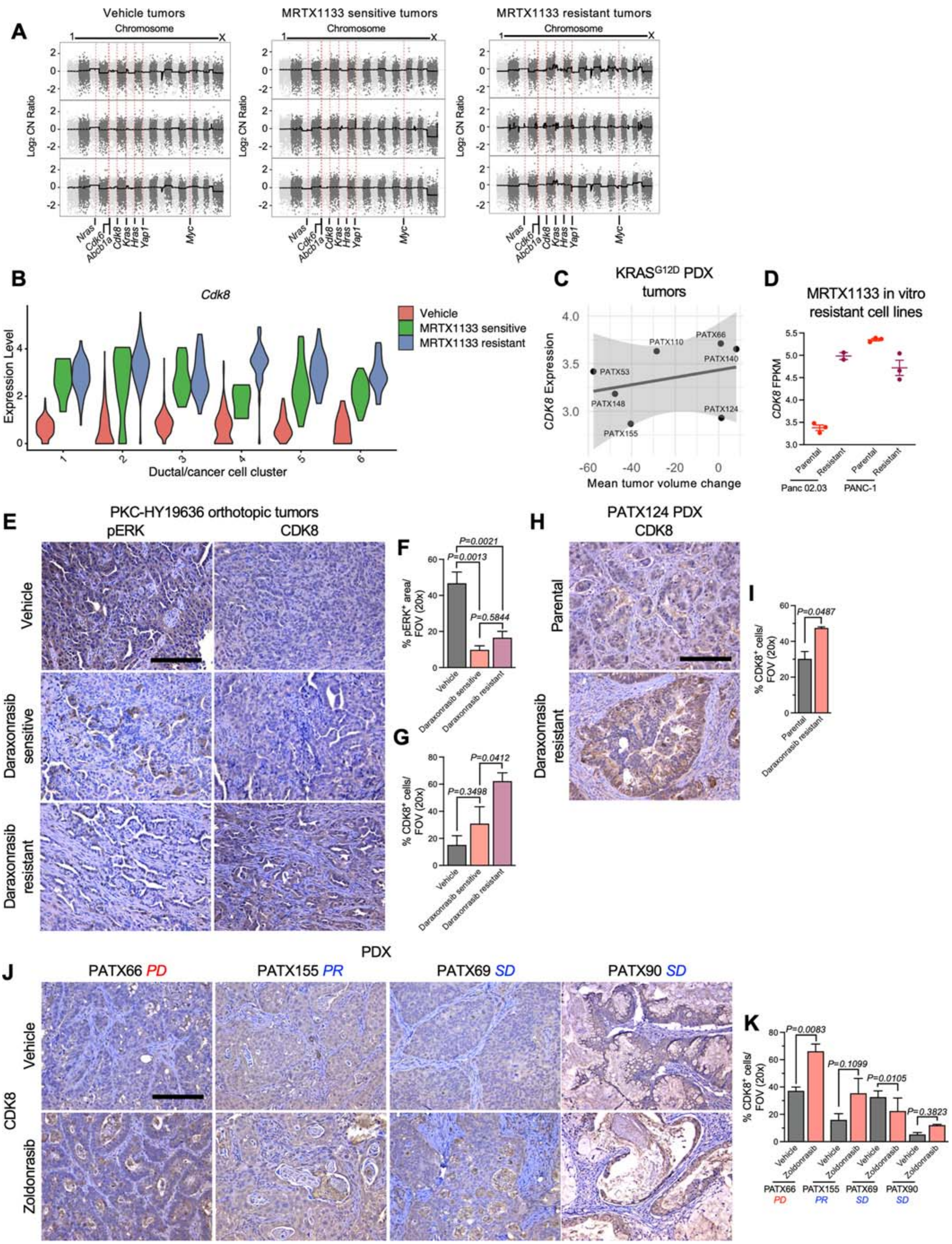
A
Vehicle tumors
MRTX1133 sensitive tumors
MRTX1133 resistant tumors
Chromosome
Log2 CN Ratio
Nras
Cdk6
Abcb1a
Cdk8
Kras
Hras
Yap1
Myc
B
Cdk8
Vehicle
MRTX1133 sensitive
MRTX1133 resistant
Expression Level
Ductal/cancer cell cluster
C
KRASG12D PDX tumors
CDK8 Expression
Mean tumor volume change
D
MRTX1133 in vitro resistant cell lines
CDK8 FPKM
Parental
Resistant
Panc 02.03
PANC-1
E
PKC-HY19636 orthotopic tumors
pERK
CDK8
Vehicle
Daraxonrasib sensitive
Daraxonrasib resistant
F
% pERK+ area/ FOV (20x)
G
% CDK8+ cells/ FOV (20x)
H
PATX124 PDX
CDK8
Parental
Daraxonrasib resistant
I
J
PDX
PATX66 PD
PATX155 PR
PATX69 SD
PATX90 SD
Vehicle
Zoldonrasib
CDK8
K

Figure EV2. CDK8 is upregulated in PDAC resistant to MRTX1133, Daraxonrasib, and Zoldonrasib.

(**A**) Genome-wide copy number plots of vehicle, MRTX1133 sensitive, and MRTX1133 resistant orthotopic PKC-HY19636 tumors with tail gDNA used as a reference. $n = 3$ tumors per group. (**B**) Violin plot of *Cdk8* expression in ductal/cancer cell clusters of vehicle, MRTX1133 sensitive, and MRTX1133 resistant orthotopic PKC-HY19636 tumors analyzed by scRNA-seq. (**C**) *CDK8* expression and mean tumor volume change of the indicated PDXs treated with MRTX1133. (**D**) *CDK8* expression in parental and MRTX1133 resistant cell lines. Data derived from GSE269985. (**E–G**) Representative images (**E**) and quantification of pERK (**F**) and CDK8 (**G**) immunostaining of orthotopic PKC-HY19636 tumors treated with daraxonrasib. Daraxonrasib treatment was started on day 12 post-orthotopic tumor implantation. Vehicle, $n = 4$; Daraxonrasib sensitive (10 days post-treatment start), $n = 3$; Daraxonrasib resistant (mice reached end point, day 22–32 post-orthotopic tumor implantation), $n = 5$. (**H**, **I**) Representative CDK8 immunostaining (**H**) and quantification (**I**) of orthotopic PATX124 parental and Daraxonrasib resistant PDXs. Parental, n = 4; Daraxonrasib resistant, $n = 2$. (**J**, **K**) Representative CDK8 immunostaining (**J**) and quantification (**K**) of PDXs treated with Zoldonrasib. PD progressive disease, PR partial response, SD stable disease. Responses reported are best responses. PATX66, $n = 3$ per group; PATX155 vehicle, $n = 4$; PATX155 Zoldonrasib, $n = 2$; PATX69 vehicle, $n = 4$; PATX69 Zoldonrasib, $n = 2$; PATX90, $n = 3$ per group. Scale bars, 100 µm. One-way ANOVA with Tukey's multiple comparisons test performed in (**F**), one-way ANOVA with Dunnett's multiple comparisons test performed in (**G**), unpaired two-tailed *t* test performed in (**I**, **K**). Exact *P* values reported.

© The Author(s)

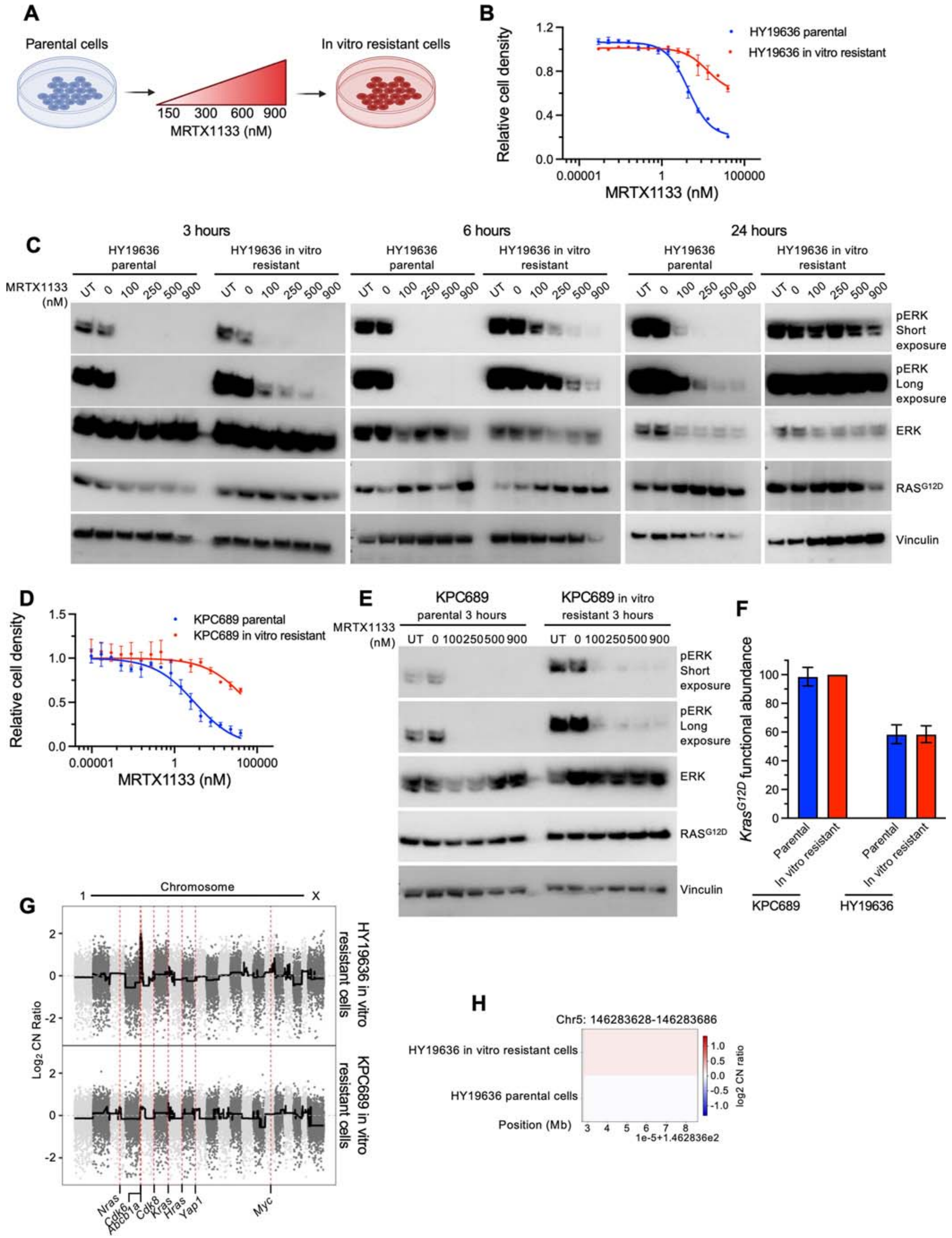
A
Parental cells
In vitro resistant cells
150 300 600 900
MRTX1133 (nM)
B
HY19636 parental
HY19636 in vitro resistant
Relative cell density
MRTX1133 (nM)
C
3 hours
6 hours
24 hours
HY19636 parental
HY19636 in vitro resistant
MRTX1133 (nM)
UT 0 100 250 500 900
pERK Short exposure
pERK Long exposure
ERK
RAS G12D
Vinculin
D
KPC689 parental
KPC689 in vitro resistant
Relative cell density
MRTX1133 (nM)
E
KPC689 parental 3 hours
KPC689 in vitro resistant 3 hours
F
Kras G12D functional abundance
Parental
In vitro resistant
KPC689
HY19636
G
Chromosome
Log2 CN Ratio
HY19636 in vitro resistant cells
KPC689 in vitro resistant cells
Nras Cdk6 Abcb1a Cdk8 Kras Hras Yap1 Myc
H
Chr5: 146283628-146283686
HY19636 in vitro resistant cells
HY19636 parental cells
Position (Mb)
log2 CN ratio

 © The Author(s)

Figure EV3. Characterization of in vitro MRTX1133 resistant cells.

(**A**) Schematic of establishment of in vitro resistant cell lines, which were generated by exposing cells to increasing concentrations of MRTX1133 over the course of 2–3 months. (**B**) Relative cell density of HY19636 parental and HY19636 in vitro resistant cells treated with the indicated concentrations of MRTX1133 for 3 days. (**C**) Representative western blots of pERK, ERK, RAS^{G12D}, and vinculin of HY19636 parental and HY19636 in vitro resistant cells treated with the indicated concentrations of MRTX1133 for 3 h, 6 h, or 24 h. (**D**) Relative cell density of KPC689 parental and KPC689 in vitro resistant cells treated with the indicated concentrations of MRTX1133 for 3 days. (**E**) Representative western blots of pERK, ERK, RAS^{G12D}, and vinculin of KPC689 parental and KPC689 in vitro resistant cells treated with the indicated concentrations of MRTX1133 for 3 h. (**F**) ddPCR for *Kras*^{G12D} functional abundance in KPC689 and HY19636 parental and in vitro resistant cells. (**G**) Genome-wide copy number plots of HY19636 and KPC689 in vitro resistant with parental cell lines used as a reference. (**H**) WES data of the *Cdk8* gene of HY19636 parental and HY19636 in vitro resistant cells. Data are presented as mean ± s.d. Source data are available online for this figure.

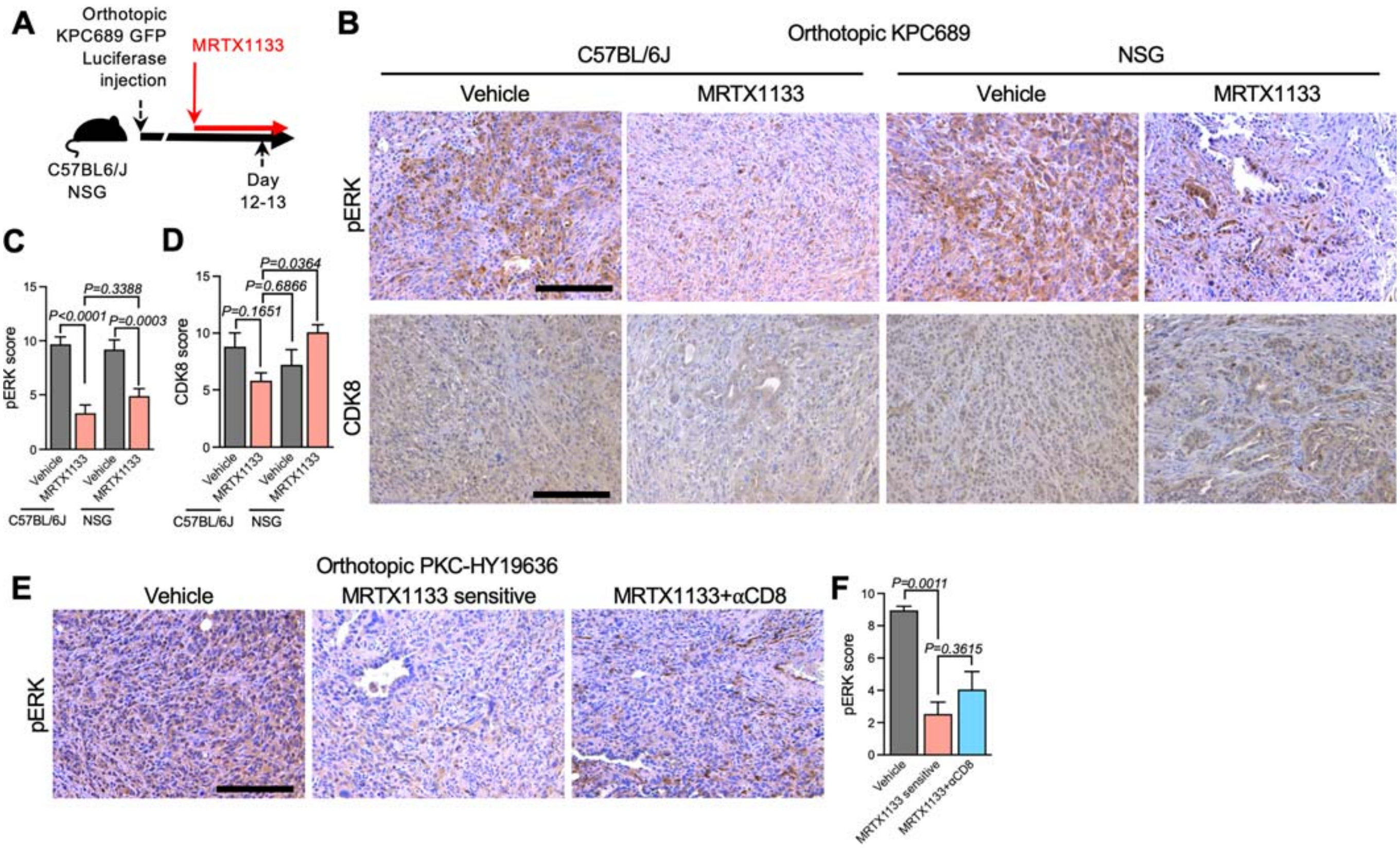

Figure EV4. T cells control the emergence of CDK8^{+} resistant cancer cells.

(**A**) Schematic of orthotopic KPC689 GFP-luciferase tumors in C57BL6/J and NSG mice treated with MRTX1133. (**B**) Representative pERK and CDK8 staining at 12–13 days post-treatment. (**C**, **D**) Quantification of pERK (**C**) and CDK8 (**D**) staining. pERK: C57BL6/J MRTX1133 and NSG vehicle, $n = 4$ mice; $n = 5$ mice per group for all other groups. CDK8: C57BL6/J MRTX1133, $n = 4$ mice; $n = 5$ mice per group for all other groups. (**E**, **F**) Representative pERK staining (**E**) and quantification (**F**) of orthotopic PKC-HY19636 tumors at 12–13 days post-treatment. Vehicle, $n = 4$; MRTX1133 sensitive, $n = 3$; MRTX1133 + αCD8, $n = 4$. Scale bars, 100 µm. One-way ANOVA with Dunnett's multiple comparisons test performed in (**C**, **D**, **F**). Exact *P* values reported.

 © The Author(s)

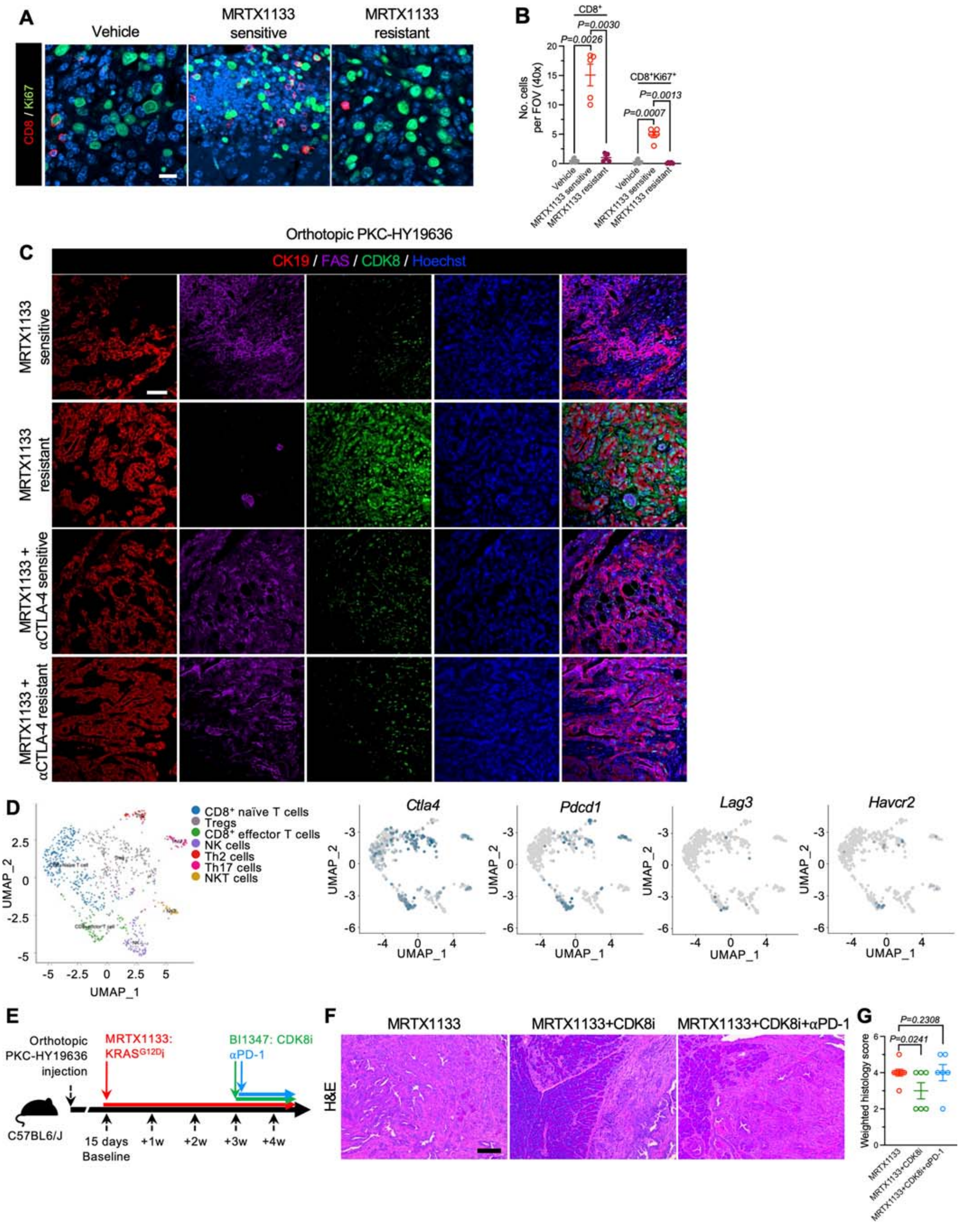

A
Vehicle
MRTX1133 sensitive
MRTX1133 resistant
CD8 / Ki67
B
CD8+
P=0.0026
P=0.0030
CD8+Ki67+
P=0.0007
P=0.0013
No. cells per FOV (40x)
Vehicle
MRTX1133 sensitive
MRTX1133 resistant
Orthotopic PKC-HY19636
C
CK19 / FAS / CDK8 / Hoechst
MRTX1133 sensitive
MRTX1133 resistant
MRTX1133 + αCTLA-4 sensitive
MRTX1133 + αCTLA-4 resistant
D
CD8+ naïve T cells
Tregs
CD8+ effector T cells
NK cells
Th2 cells
Th17 cells
NKT cells
UMAP_1
UMAP_2
Ctla4
Pdcd1
Lag3
Havcr2
E
Orthotopic PKC-HY19636 injection
MRTX1133: KRASG12Di
BI1347: CDK8i
αPD-1
C57BL6/J
15 days Baseline
+1w
+2w
+3w
+4w
F
MRTX1133
MRTX1133+CDK8i
MRTX1133+CDK8i+αPD-1
H&E
G
P=0.2308
P=0.0241
Weighted histology score
MRTX1133
MRTX1133+CDK8i
MRTX1133+CDK8i+αPD-1

Figure EV5. T cell responses in resistant tumors.

(**A**, **B**) Representative images (**A**) and quantification of $CD8^+$ and $CD8^+Ki67^+$ cells (**B**). Vehicle, $n = 5$, MRTX1133 sensitive, $n = 5$, MRTX1133 resistant, $n = 5$. Scale bar, 20 µm. (**C**) Single channel representative images of CK19 (red), FAS (purple), and CDK8 (green) stained orthotopic PKC-HY19636 tumors. Images from Fig. 5F are presented again here with each channel included individually. Scale bar, 50 µm. (**D**) UMAP of T cells of orthotopic PKC-HY19636 tumors and expression of checkpoint molecules determined by scRNA-seq. (**E**) Schematic of orthotopic PKC-HY19636 tumors treated with MRTX1133, CDK8i (BI1347), and αPD-1. (**F**, **G**) Representative H&E images (**F**) and quantification (**G**) of orthotopic PKC-HY19636 tumors. MRTX1133, $n = 8$; MRTX1133 + CDK8i, $n = 6$; MRTX1133 + CDK8i + αPD-1, $n = 6$. Scale bar, 100 µm. Brown–Forsythe and Welch ANOVA with Dunnett T3's multiple comparisons test performed in (**B**). Chi-square test performed comparing responsive (score≤2) to non-responsive (score>2) in (**G**). Data are presented as mean ± s.e.m. Exact *P* values reported.

 © The Author(s)
